# Beyond Conventional Cooling: Advanced Micro/Nanostructures for Managing Extreme Heat Flux

**DOI:** 10.1002/adma.202504706

**Published:** 2025-11-17

**Authors:** Yuankun Zhang, Huajie Li, Yuhang Zhou, Jun Ma, Keng‐Te Lin, Han Lin, Chunsheng Guo, Baohua Jia

**Affiliations:** ^1^ Centre for Omniscale Thermal Management and Comprehensive Energy Utilization (OTM‐EU) School of Airspace Science and Engineering Shandong University Weihai 264209 China; ^2^ School of Science, Computing and Engineering Technologies Swinburne University of Technology Hawthorn Victoria 3122 Australia; ^3^ Center for Atomaterials and Nanomanufacturing (CAN) School of Science RMIT University Melbourne Victoria 3000 Australia; ^4^ Shenzhen Research Institute of Shandong University Shenzhen 518057 China; ^5^ ARC Training Centre for Surface Engineering for Advanced Materials (SEAM) RMIT University Melbourne Victoria 3000 Australia

**Keywords:** advanced design and manufacturing, heat transfer mechanism, metamaterial, micro/nanostructures, targeted thermal management

## Abstract

The surge in device integration has escalated thermal losses, compromising performance and safety, necessitating advanced thermal management solutions with extraordinary heat flux capabilities to address power and heat dissipation challenges in high heat‐flux electronics. Micro/nanostructures have emerged as promising solutions for targeted heat dissipation in electronics due to their outstanding performance, miniature footprint, and design flexibility. However, a comprehensive review of recent advancements in heat transfer control via micro/nanostructures and their current and potential applications for high heat‐flux thermal management, particularly for electronic devices, remains lacking. This review systematically examines the fundamental heat transfer mechanisms enabled by micro/nanostructures at multiscales. A wide range of bio‐inspired or engineered designs are highlighted as formidable candidates for highly efficient thermal and hydrodynamic metamaterials. Novel micro/nano‐patterns that significantly contribute to the modulation of coupled heat transfer processes in practical applications for electronic thermal management are elaborated. Furthermore, the strengths and limitations of existing design and manufacturing methods for micro/nanostructures are comparatively summarized. Finally, key challenges and prospects of micro/nanostructure‐based thermal management techniques are discussed, drawing insights from previous applications. This review underscores the transformative potential of micro/nanostructures in achieving reliable, sustainable, and targeted thermal management for high‐performance electronic devices in the near future.

## Graphical abstract

This review examines the design, application, and manufacturing of biomimetic or engineered micro/nanostructures for managing high heat‐flux in multi‐level electronics by enhancing conductive, convective, phase‐changing, and radiative heat transfer mechanisms, highlighting their potential for efficient, targeted thermal management, and future prospects.

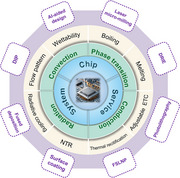

## 1 Introduction

With the rapid growth of societal demand for computing, the requirements for electronic devices across applications such as communications, personal electronics, and aerospace are becoming imminent. In high‐performance electronic devices (e.g., supercomputers, AI desktop computers, and edge computing devices) and compact mobile devices (e.g., smartphones, tablet computers, and extended reality devices), power and heat dissipation constraints have become essential considerations in their design processes.^[^ ^1^, ^2^, ^3^, ^4^ ^]^ Recent advancements in electronics are characterized by two major trends, including high‐integration and AI hardware surge, significantly escalating their heat‐flux densities (500–1000 Wcm^−2^).^[^ ^5^ ^]^ Consequently, electronic thermal management has been of considerable significance due to the following aspects, including maintaining the long‐term performance and lifespan of devices,^[^ ^6^, ^7^, ^8^ ^]^ optimizing energy utilization efficiency,^[^ ^9^, ^10^ ^]^ promoting the miniaturization and integration of equipment,^[^ ^11^ ^]^ and supporting diverse workloads and extreme conditions.^[^ ^12^ ^]^

Thermal management refers to controlling and optimizing heat generation, transfer, and dissipation in electronic devices through various techniques and strategies to maintain stable operation within the normal operating temperature range, thereby guaranteeing performance, reliability, and longevity.^[^ ^13^ ^]^ The primary goal of thermal management systems is to balance the heat dissipation requirements and heat generation status of the central components, preventing performance degradation, hardware damage, and energy waste caused by overheating. Depending on the scale of the devices and heat dissipation requirements, it can be divided into chip level, board level, and system level.^[^ ^8^ ^]^ At the chip level, where heat is initially generated, the focus is on efficient heat transfer from the processor to the package and subsequently to the heat sink. The miniaturization of the electronic system poses significant challenges in addressing thermal management issues related to localized hot spots and size effect,^[^ ^14^, ^15^, ^16^, ^17^ ^]^ which necessitate careful consideration across the design process, product reliability, computational performance, and manufacturing cost. At the board level, the process entails the conduction and diffusion of heat through printed circuit boards (PCBs), along with the convection and diffusion of heat into the ambient air, highlighting reasonable arrangements of electronic components with large heat generation and their cooling devices. Thermal management at the system level involves optimal design of equipment layout to facilitate the flow efficiency of the coolant (gas/liquid) while minimizing volume and/or mass utilization, development of an intelligent temperature control system to control the cooling modules dynamically, and the design of environmental adaptability for reliable operation under extreme temperature conditions.^[^ ^9^, ^18^, ^19^ ^]^ This scale emphasizes heat transfer across large spatial scales and the overall performance of the thermal system, ensuring that the environmental parameters of the system remain within established safety limits. It is important to note that conventional cooling strategies are fundamentally constrained by physical limitations. For example, air cooling can hardly exceed 500 W per chip due to the inherently low thermal conductivity and heat capacity of air.^[^ ^20^ ^]^ In contrast, water‐based cooling systems are capable of dissipating heat at the kilowatt scale. However, this requires prohibitively high flow rates (15–20 Lmin^−1^ for NVIDIA HGX system).^[^ ^21^ ^]^ The commonly used phase change materials can achieve heat absorption capacities of up to 300 kJkg^−1^ with limited cycles.^[^ ^22^, ^23^ ^]^

Introducing micro/nanostructures has significantly improved the cooling systems for electronics, particularly addressing the challenges posed by increasing power densities and miniaturization. Specifically, micro/nanostructures permit high integration within the confined space of integrated circuits (ICs), reducing the heat transfer pathway and facilitating their miniaturization without excess energy consumption. By tailoring their shapes, sizes, and arrangements, micro/nanostructures can be customized to meet specific heat dissipation requirements and integrate with other functions.^[^ ^24^ ^]^ In terms of thermal conduction, nanostructured materials with exceptional thermal conductivity enhance heat transfer efficiency, while anisotropic thermal conductivity in metamaterials allows for precise control of heat flow patterns. On the other hand, the unique surface morphology of micro/nanostructures can modify fluid flow patterns, thereby strengthening convective heat transfer by thinning the boundary layer of liquid flow, or guiding airflow and even enabling self‐driven fluid flow for continuous heat dissipation. Furthermore, specially designed micro/nanostructures enable altering surface optical properties to enhance thermal radiation efficiency by adjusting the wavelength and direction of thermal emission and reflection. Previous studies have demonstrated that state‐of‐the‐art micro/nanostructured thermal management systems offer improved heat dissipation by leveraging the unique properties of materials and surface geometries at micro/nanoscales.^[^ ^1^, ^2^, ^7^, ^10^, ^24^, ^25^, ^26^, ^27^ ^]^ However, the existing literature remains fragmented, lacking a comprehensive review on the role of micro/nanostructures in improving targeted thermal management at each level rather than taking measures after the heat spreads throughout, particularly in understanding heat transfer mechanisms within electronic devices at different scales and thermal flow manipulation via specially designed micro/nanostructured configurations.

**Figure 1:**
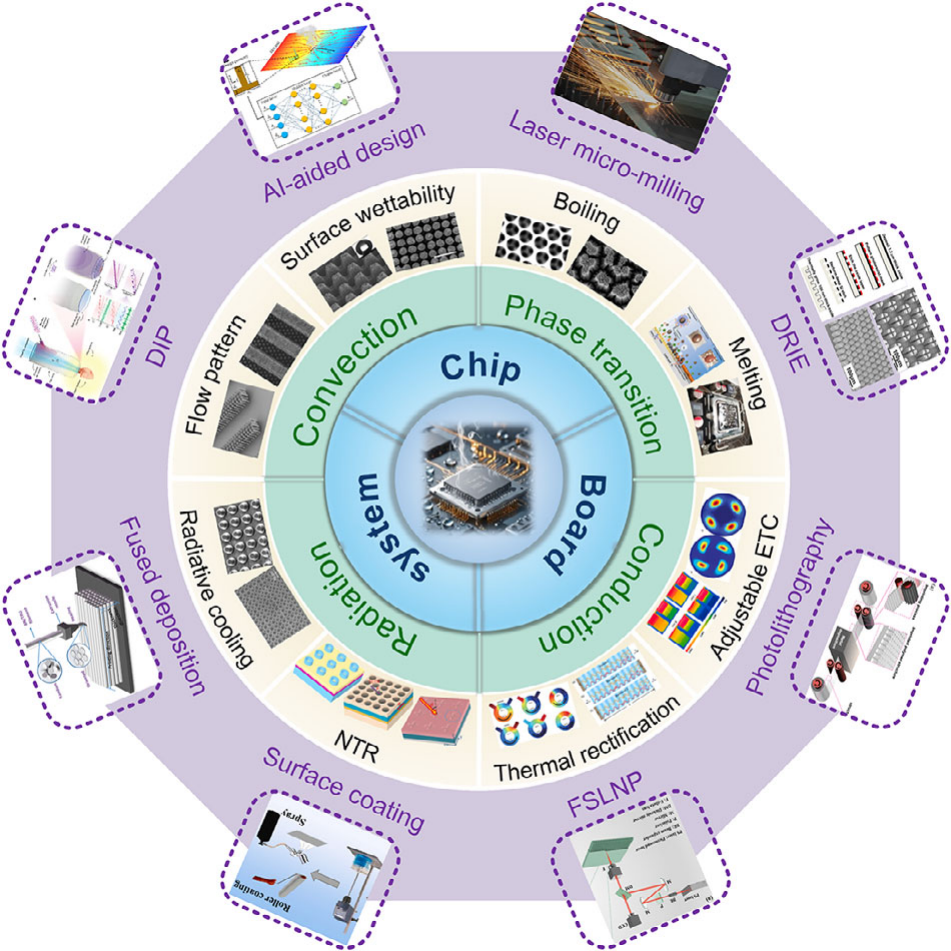
Schematic representation of the critical sections of micro/nanostructures used for thermal management of electronics, including fundamental heat transfer principles (conduction, convection, and radiation) associated with multiple electronic levels (chip, board, and system), typical micro/nanostructures for corresponding cooling strategies, as well the design and manufacturing methods used for the fabrication of previous micro/nanostructure‐based cooling systems. ETC, NTR, DIP, DRIE, and FSLNP denote effective thermal conductivity, non‐reciprocal thermal radiation, dynamic interface printing, deep reactive ion etching, and femtosecond laser nanoprinting, respectively.

As shown in **Figure** **1**, this review focuses on advanced thermal management techniques for high heat‐flux removal using specially designed micro/nanostructures in electronics with high integration and power densities. Section 1 introduces the necessity of thermal management for electronics on multiple scales, the basic forms of thermal management, and the advantages of micro/nanostructures compared with traditional cooling strategies. The underlying physics of heat management via different heat transfer principles, i.e., conduction, convection, phase transition, and radiation, are consequently discussed under multiple‐level electronic cases (Section 2). The key factors influencing the heat transfer efficiency of the three mechanisms and their applications of representative micro/nanostructures in thermal management are explored in Sections 3, 4, 5, 6, respectively. The characteristics of the latest micro/nanoscaled design and manufacturing technologies are summarized in Section 7. Section 8 outlines the existing practice of micro/nanostructures employed in thermal interface materials (TIMs) and heat sinks for electronic cooling, emphasizing their achievement results and limitations. Finally, potential strategies that can be extrapolated to multi‐scale electronic systems to facilitate targeted thermal management are suggested in Section 9.

## 2 Thermal Management Mechanisms in Electronic Systems

### 2.1 Thermal Management Process of Electronic Systems

**Figure 2:**
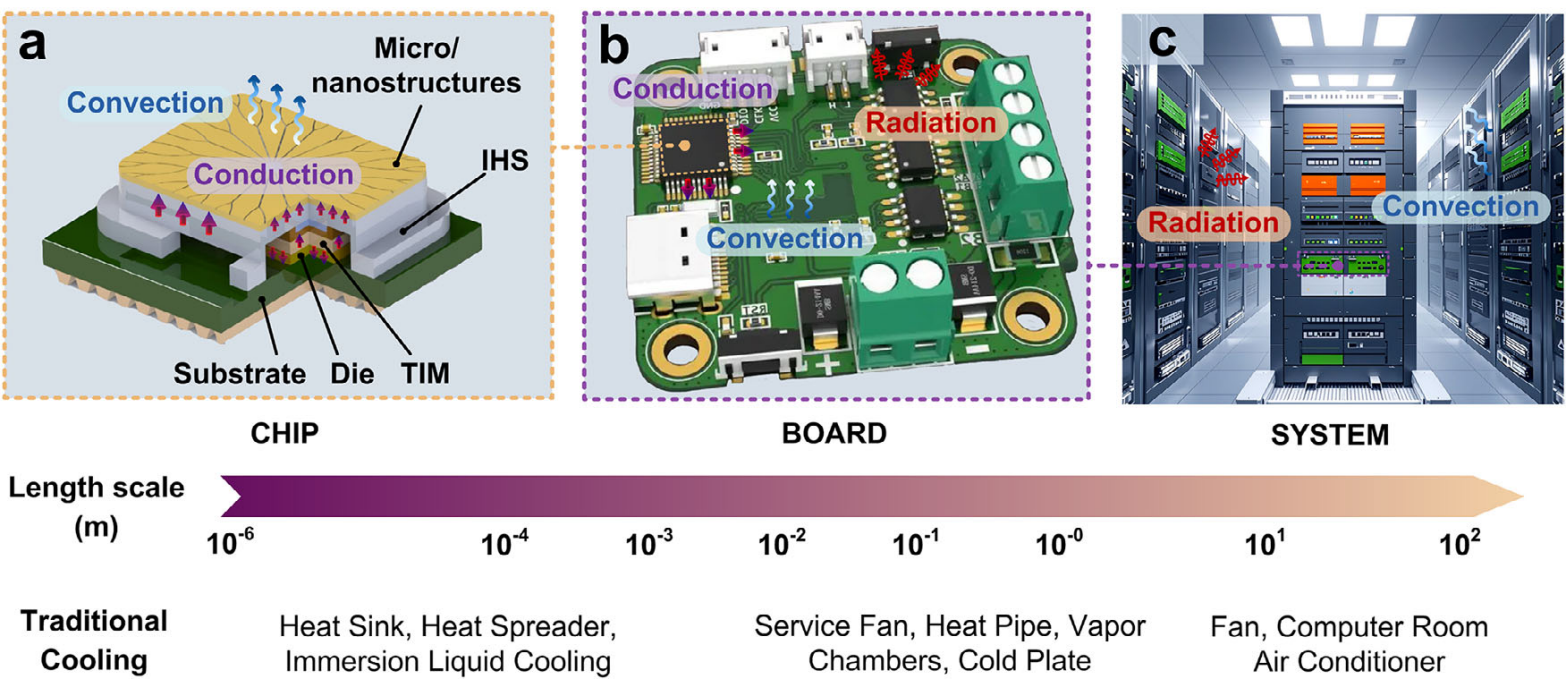
Schematic of electronic cooling at various spatial scales and heat transfer patterns, including a) the chip, b) the board, and c) the system levels.

Thermal management in electronic devices is a crucial part that guarantees their reliable operation. It begins with heat generation within chips or other heat source modules, as shown in **Figure** **2**a, which produces substantial heat during transistor switching operations for instruction execution. The resistance in transistors and other circuit elements causes electrical energy to convert into thermal energy, resulting in heat accumulation. The heat is then transferred to the chip packaging material via conduction, which diffuses from the packaging material to the PCB (Figure 2b). The thermal conductivity of the PCB varies due to its complex structure, which includes multiple layers of copper foil and insulating materials. Special PCB designs in high‐performance devices, such as thicker copper layers or better insulating materials, enhance heat transport within the board to diffuse along the temperature gradient.^[^ ^25^ ^]^

If a heat sink is present, the heat is conducted from the electronic board, typically constructed from highly thermally conductive metals with large surface areas to maximize heat spreading efficiency. Heat is transferred to the surrounding air or cooling medium via thermal convection as the heat sink warms up. Simultaneously, the heat sink radiates heat to the colder environment (Figure 2c). In the case of liquid‐immersed cooling and two‐phase systems, the heat is directly carried away by the flowing liquid or coolant evaporation. Finally, through convection and radiation, the heat dissipates from the heat sink into the surroundings, where heat is continuously transported until fully dispersed. This entire heat dissipation process, from the initial heat generation in chips to the final dissipation, is essential for ensuring optimal performance and longevity of electronics.

### 2.2 Heat Transfer Principles within Electronic Cooling

#### 2.2.1 Conduction

Thermal conduction refers to the process by which heat is transferred along with a temperature gradient through the thermal vibration of molecules, atoms, or electrons in a substance from a microscopic perspective.^[^ ^28^ ^]^ In electronic devices, heat is conducted between solid components such as heating modules, TIMs, circuit boards, and casings. Thermal conduction complies with Fourier's law microscopically, as shown below,

(1) $$\frac{d\phi }{dt}=-\lambda A\frac{dT}{dx}$$

where d*ϕ*/d*t* represents the heat conduction rate, W. $\frac{dT}{dx}$ is the temperature gradient along the *x* direction, Km^−1^. *A* is the heat transfer area, m^2^, and *λ* is the thermal conductivity, W(m·K)^−1^.

The rate of heat dissipation depends on the thermal conductivity of the material. In addition, the path of conduction also affects the heat dissipation effect. A specially designed heat conduction path that aligns with the route of low thermal resistance can improve the heat transfer efficiency.

**Figure 3:**
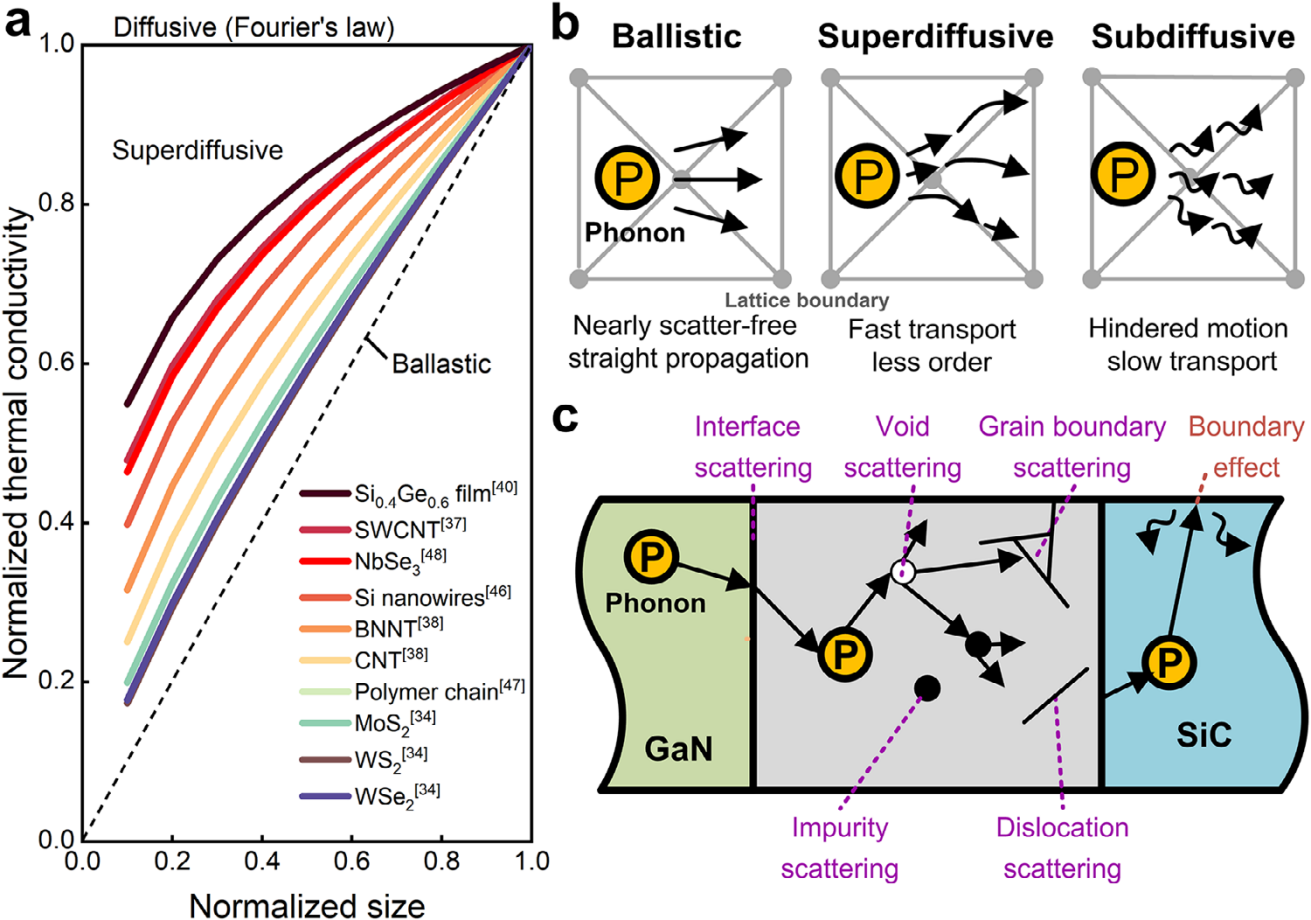
a) Numerical and experimental results of size‐dependent nanoscaled thermal conduction of various materials.^[^ ^34^, ^37^, ^38^, ^40^, ^47^, ^48^ ^]^ b) Schematics of three typical phonon propagation modes. c) A schematic diagram of phonon transport within nanostructured materials illustrating different scattering and boundary effects.

However, when addressing the heat transfer process at micro/nano scales in integrated electronic devices, e.g., third‐generation semiconductor chips containing more than 100 layers with a total thickness of 10.2 µm,^[^ ^29^ ^]^ heat flows along the vertical direction (1D) and in‐plane direction (2D), both of which involve micro/nanoscaled thermal conduction processes.^[^ ^30^ ^]^ Since Rieder et al.^[^ ^31^ ^]^ revealed the failure of Fourier's law in heat conduction of 1D harmonic lattices in 1967. Numerous related studies have shown that phonon transport at micro/nanoscales transitions from a diffusive regime (described by classic Fourier's law) to a ballistic or super‐diffusive regime governed by size effects, boundary scattering, and long phonon mean free paths.^[^ ^32^, ^33^, ^34^, ^35^, ^36^, ^37^, ^38^, ^39^, ^40^, ^41^ ^]^ Some classic reviews have provided in‐depth analyses of the anomalous thermal transport phenomena of materials. For example, Yang et al.^[^ ^42^ ^]^ introduced the anomalous diffusion in low‐dimensional materials, and Zhang et al.^[^ ^40^ ^]^ summarized the size effects of low‐dimensional nanomaterials in 2020. Besides, the theories and regulation methods of phonon heat conduction in 1D, 2D systems, and at interfaces were reviewed by Luo et al.^[^ ^30^ ^]^ and Chen et al.^[^ ^43^ ^]^ recently. Due to the size confinement and structural constraints, thermal conductivity of materials (*λ*) becomes size‐dependent, i.e., length (*L*) of chains or thickness (*δ*) for films, with an exponential relationship (*β*), diverging as a power law (*λ*∝*L^β^*) in the 1D system, directly violating the fundamental assumption of Fourier's law that *λ* is a size‐independent material property.^[^ ^44^, ^45^, ^46^ ^]^ Theoretically, non‐equilibrium molecular dynamics (NEMD) simulations consistently showed size‐dependent thermal conductivity in ideal models (e.g., Fermi‐Pasta‐Ulam, FPU,^[^ ^36^ ^]^ and Frenkel‐Kontorova, FK^[^ ^44^ ^]^) and real materials (Single‐walled carbon nanotubes, SWCNTs,^[^ ^37^ ^]^ Si nanowires,^[^ ^46^ ^]^ polymer chains^[^ ^47^ ^]^) with varying *β*. Experimental evidence on the length (*L*)/thickness (*δ*)‐dependent of *λ* includes carbon nanotubes (CNTs), boron nitride nanotubes (BNNTs),^[^ ^38^ ^]^ NbSe_3_ nanowires,^[^ ^48^ ^]^ and Si_0_._4_Ge_0_._6_ thin films (*δ*).^[^ ^40^ ^]^ Besides, 2D materials, including suspended monolayer graphene, MoS_2_, WS_2_, and WSe_2,_^[^ ^34^ ^]^ exhibited logarithmic divergence of *λ* along the heat transfer pathway. **Figure** **3**a illustrates the size dependence across distinct phonon transport regimes. As *β* approaches 1, the behavior converges to the ballistic limit (Figure 3b), whereas *β* = 0 corresponds to the diffusive motion governed by Fourier's law. The crossover between these propagation modes arises from boundary and intrinsic scattering limitations.

The connection to the anomalous diffusion mechanism provides a fundamental physical explanation for this breakdown.^[^ ^32^, ^49^, ^50^ ^]^ As shown in Equation (2), it bridges the microscopic dynamics of phonon motion with the macroscopic thermal conduction behavior based on the quantitative relationships between the heat conduction divergence exponent (*β*) and the anomalous diffusion exponent (*a*), which not only enriches the phenomenological theoretical system of thermal transport but also has profound implications for electronic thermal management applications.

(2) $$\begin{array}{cc} \mathrm{Diffusion} & \mathrm{Thermal}\;\mathrm{conduction}\\ \Gamma^{2}=2Dt^{a} & \lambda ∼L^{\beta }\\ a<1,\;\mathrm{Subdiffusion} & \beta <0,\;\mathrm{Convergent}\;\lambda \\ a=1,\;\mathrm{Normal}\;\mathrm{diffusion} & \;\;\;\beta =0,\;\mathrm{Fourier}\;\;\mathrm{law},\;\;\lambda =\mathrm{constant}\\ a>1,\;\mathrm{Superdiffusion} & 0<\beta <1,\;\mathrm{Divergent}\;\kappa \\ a=2,\;\mathrm{Ballistic}\;\mathrm{motion} & \beta =1,\;\lambda \propto L \end{array}$$

where *D* represents the diffusion coefficient. *t* is time, s. ‹*Γ* ^2^› is the mean value of the squared displacement of system diffusion at time *t*.

Phonon propagation and scattering are essential for the foundation of the theoretical framework when heat flows in a nanoscale structure,^[^ ^51^, ^52^, ^53^, ^54^, ^55^ ^]^ which is governed by the interplay of dispersion relations, phonon scattering mechanisms, and boundary effects (Figure 3c).^[^ ^56^, ^57^ ^]^ Based on calculations of phonon lifetimes and collision kernels, the phonon Boltzmann transport equation (BTE) method provides a rigorous kinetic description of phonon propagation paths,^[^ ^58^ ^]^ as shown below.

(3) $$\frac{\partial n\left(\vec{k}j\right)}{\partial t}={\left.\frac{\partial n\left(\vec{k}j\right)}{\partial t}\right|}_{coll}-\vec{v}\left(\vec{k}j\right)\cdot \nabla n\left(\vec{k}j\right)$$

where ∂*n*($\vec{k}$ *j*)/∂*t*|*_coll_* represents the collision term, indicating the rate at which the phonon distribution *n*($\vec{k}$ *j*) changes caused by collisions. $\vec{k}$ is the wave vector. *j* is the polarization index, and $\vec{v}$($\vec{k}$ *j*) is the group velocity.

To solve the linearized form of the previous equation for small thermal gradients (time‐independent), one therefore requires detailed knowledge of phonon dispersion (∂*ω*($\vec{k}$ *j*)), the group velocity ($\vec{v}$($\vec{k}$ *j*)), and scattering processes (captured by lifetimes *τ*($\vec{k}$ *j*) or the full collision kernel). Phonon scattering arises from anharmonic interactions, point defects, and grain boundaries,^[^ ^52^ ^]^ with Umklapp processes being critical for thermal resistance.^[^ ^59^ ^]^ In nanostructures, boundary scattering dominates when dimensions are smaller than phonon mean free paths, requiring modified BTE solutions incorporating geometry‐dependent phonon trajectories.^[^ ^60^ ^]^ For superlattices and rough interfaces, phonon dispersion and scattering rates may deviate significantly from bulk behavior, necessitating atomistic or wave interference models.^[^ ^61^ ^]^

#### 2.2.2 Convection

Single‐phase convection (gas/liquid) in electronic thermal management indicates the process where heat generated by the electronics is transferred away through the movement of a fluid, typically air or liquid. As the chip operates, the generated heat causes the surrounding fluid to warm up, which exchanges the heat with cooler fluid. This continuous cycle of fluid movement effectively transfers heat away from chips, boards, and systems, preventing overheating and maintaining their performance. Convection cooling can be natural (relying on buoyancy forces) or forced (using fans or micro‐pumps). In natural convection, the heated, less dense fluid naturally rises away from the heat source due to buoyancy forces, while cooler fluid from the surroundings flows in as an alternative.^[^ ^62^ ^]^ On the other hand, a fan or pump actively moves the coolant over the heat source surface in forced convection, pushing away the heated fluid and drawing in cooler fluid to maintain an intense heat exchange. According to Newton's law of cooling Equation (4), the convection mode determines the convective heat transfer coefficient (*h*), thereby significantly affecting the efficiency of convective heat transfer. Increasing the flow rate of the fluid can enhance the effect of heat convection, while it will also increase energy consumption.

(4) $$\frac{d\phi }{dt}=hA\left(T_{w}-T_{\infty }\right)$$

where d*ϕ*/d*t* represents the heat transfer rate (the unit is W). *A* is the heat transfer area (the unit is m^2^). *h* is the heat transfer coefficient, W(m^2^·K)^−1^. *T*_w_ and *T*_∞_ are the temperatures of the solid and the coolant, respectively, (the unit is K).

#### 2.2.3 Phase Transition

The heat transfer principle related to melting and vaporization used for thermal management is separated from single‐phase convection in this review, considering their superior heat transfer abilities. Heat absorption via melting essentially refers to the process where a material absorbs latent heat to overcome the binding forces between molecules/atoms during the phase transition (solid to liquid).^[^ ^63^ ^]^ Classical models primarily describe this process from the perspectives of macro thermodynamics and dynamics, including the equilibrium model and the heat conduction‐phase transition coupling model (i.e., Stefan model).^[^ ^64^, ^65^ ^]^ The former focused on evaluating the energy essence of melting heat absorption, while the latter quantified the dynamic process characterizing the movement of the phase transition interface. In melting heat absorption models, micro/nanostructure‐induced modulation of capillary forces and surface wettability may further complicate the energy balance at the moving phase interface in the Stefan problem.

(5) $$\lambda_{l}\frac{\partial T_{l}}{\partial x}-\lambda_{s}\frac{\partial T_{s}}{\partial x}=\rho \dot{L}v_{n}$$

where the $\dot{L}$ represents the latent heat, J/kg. λ*_l_* (∂*T_l_*/∂*x*) and λ*_s_* (∂*T_s_*/∂*x*) are the heat flux difference between the liquid and solid sides (the unit is Wm^−2^). *v*_n_ is the interface movement velocity, (the unit is ms^−1^). Limitations of this model include neglecting effects such as supercooling or superheating,^[^ ^66^ ^]^ and constant thermophysical properties, which thereby pose challenges in solving problems with complex geometries.^[^ ^65^ ^]^ When considering the influence of micro‐/nanostructures on the energy balance at the interface, combining computational fluid dynamics (CFD) modeling and the equations can enhance compatibility with complex structures.

The heat transfer mechanism of vaporization in micro/nanostructures involves complex interactions between the solid surface and the two‐phase fluid. Evaporation is the process by which molecules at the surface of a liquid gain sufficient kinetic energy to transform the liquid phase into the gas phase, fundamentally driven by molecular thermal motion and interfacial interactions.^[^ ^28^ ^]^ Hertz‐Knudsen correlation^[^ ^67^ ^]^ can quantitatively describe the relationship between evaporation flux and saturated vapor pressure.

(6) $$J=\gamma \left(\frac{P_{sat}\left(T\right)-P_{v}}{\sqrt{2\pi MRT}}\right)$$

where the *J* is evaporation flux (the unit is kg·m^−2^·s^−1^). *γ* is the evaporation coefficient, taken as (0,1) to characterize non‐equilibrium effects, *P*_sat_ and *P_v_* are the saturated vapor pressure at *T*, and partial vapor pressure (the unit is Pa). M represents the molar mass of the liquid (the unit is g·mol^−1^). The *R* is the universal gas constant, taken as 8.314 J(mol·K)^−1^. The connection between *P*_sat_ and *T* is given as,^[^ ^63^ ^]^

(7) $$\frac{dP_{sat}}{dT}=\frac{L_{v}}{T\left({\dot{V}}_{g}-{\dot{V}}_{l}\right)}\dot{=}\frac{L_{v}P_{sat}}{RT^{2}}$$

where the *L*_v_ represents the molar heat of vaporization, (the unit is kJ/mol). $\dot{V}$_g_ and $\dot{V}$_l_ are molar volumes of vapor and liquid phases (the unit is Lmol^−1^).

Therefore, key factors that affect the evaporation efficiency include temperature, vapor pressure gradient, surface area and interface properties, liquid properties, and other environmental conditions.^[^ ^68^ ^]^ However, evaporation on micro/nanoscales highlights the anomalous effect on the vapor pressure difference (*P*_sat_‐*P_v_*) and liquid enthalpy of vaporization (*L*_v_) when the liquid is confined at these scales. The former is mainly affected by the Kelvin effect^[^ ^69^, ^70^, ^71^ ^]^ and Knudsen effect,^[^ ^72^, ^73^ ^]^ resulting in modifications of the *P*_sat_ and nonlinear *P*_v,_ respectively. The latter, on the other hand, is attributed to the reduction in enthalpy of vaporization caused by breaking hydrogen bond networks within nanoscale confinement. For boiling, given that the nucleate boiling region is the most effective for surface heat transfer along the well‐known boiling curve,^[^ ^74^ ^]^ it becomes a key process characterized by the formation, growth, and departure of vapor bubbles at nucleation sites.^[^ ^75^, ^76^ ^]^ While beyond this region, the critical heat flux phenomenon causes a sudden drop in heat transfer coefficient (transition and film boiling) and finally the burnout.^[^ ^75^ ^]^ Specifically, micro/nanocavities on the solid surface stabilize vapor nuclei by reducing the free energy required for nucleation, thereby promoting the inception of bubbles. Bubble growth is then driven by evaporation of the superheated microlayer beneath bubbles, until detaching when the force balance between buoyancy and surface adhesion is broken. The forces acting on a typical growing bubble attached to the heated surface include surface tension force (*F*_s_), buoyant force (*F*_b_), lift force (*F*_L_), bubble inertial force (*F*_bi_), and unsteady growth force (*F*_g_).^[^ ^77^, ^78^, ^79^ ^]^ Buoyancy and lifting force promote the bubble to detach from the surface, while others hinder the bubble from growing and leaving. Therefore, the force balance on a bubble can be theoretically given as:

(8) $$\vec{F_{b}}+\vec{F_{l}}=\vec{F_{s}}+\vec{F_{g}}+\vec{F_{bi}}$$

(9) $$F_{b}=V_{b}\left(\rho_{l}-\rho_{v}\right)g$$

where *V*_b_ represents the volume when the bubble leaves (the unit is m^3^). *ρ*_l_ and *ρ*_v_ are the liquid and vapor densities (the unit is kgm^−3^). *g* is gravitational acceleration, taken as 9.81 m^2^s^−1^.

(10) $$F_{L}=\frac{\pi }{2}\rho_{l}{\left(d_{b}v_{b}\right)}^{2}C_{L}$$

where *v*_b_ represents the rise velocity of the bubble, empirically characterized as 2d*r*_b_/d*t* (the unit is ms^−1^).^[^ ^80^, ^81^ ^]^ *C*_L_ is the empirical constant of lift force obtained by fitting the bubble department diameters (the unit is N).^[^ ^78^, ^82^ ^]^

(11) $$F_{s}=\pi d_{c}\sigma \sin\theta$$

where *d*_c_ is the contact diameter of the growing bubble (the units is m). *σ* is the surface tension coefficient (this unit is Nm^−1^). *θ* is the angle between the bubble and the surface, which can be expressed as sin^−1^(*d*_c_/*d*_b_).

(12) $$F_{g}=\rho_{l}\pi r_{b}^{2}\left(\frac{3}{2}C_{s}{\left(\frac{dr_{b}}{dt}\right)}^{2}+r_{b}\frac{d}{dt}\left(\frac{dr_{b}}{dt}\right)\right)$$

where *C*_s_ is an empirical constant.^[^ ^80^ ^]^ Inertial forces result from an upward trend as the bubble grows,^[^ ^82^ ^]^ which can be given as below for linear bubbles:

(13) $$F_{bi}=m_{v}\frac{d}{dt}\left(\frac{dr_{b}}{dt}\right)+\frac{dm}{dr_{b}}{\left(\frac{dr_{b}}{dt}\right)}^{2}$$

where *m*_v_ is vapor mass (the unit is kg). The bubble radius is governed by the balance between surface tension and pressure difference, which can be expressed as

(14) $$r_{b}=\frac{2\sigma }{P_{v}-P_{l}}$$

where *P*_v_ and *P*_l_ are the vapor and the liquid pressures (the unit is Pa).

Enhanced surfaces reduce bubble departure diameter and increase nucleation site density, intensifying latent heat transfer and liquid agitation. Consequently, introducing micro/nanostructures can effectively improve the heat exchange area, bubble departure frequency, latent heat transfer, and fluid replenishment, by tailoring surface properties such as roughness,^[^ ^83^, ^84^, ^85^ ^]^ wettability,^[^ ^86^, ^87^ ^]^ and capillary wickability.^[^ ^88^, ^89^ ^]^

#### 2.2.4 Radiation

Radiative cooling is a passive cooling technique that relies on the natural emission of thermal radiation to cooler objects or environments, especially at night or in dry, clear conditions. The mechanism and process of radiative cooling work on basic thermodynamic principles, specifically the Stefan‐Boltzmann law,^[^ ^90^ ^]^ which states that any object emits radiation proportional to its temperature.^[^ ^91^ ^]^ All objects with a temperature higher than absolute zero emit infrared radiation based on their temperature, as depicted in Equation (15).

(15) $$\frac{d\phi }{dt}=\varepsilon \sigma_{s}A\left(T^{4}-{T_{\infty }}^{4}\right)$$

where $\frac{d\phi }{dt}$ represents the heat transfer rate, W. *A* is the heat transfer area, m^2^, *ε* is the emissivity of the object (0‐1), *σ*_s_ is the Stefan‐Boltzmann constant, taken as 5.67 × 10^−8^ W(m^−2^·K^−4^). *T* and *T*_∞_ are the temperatures of the object and the colder environment respectively, K.

In electronic devices, heat radiation mainly occurs between the surface of heating components and the surrounding environment. Thermal radiation does not require the participation of a medium and can be carried out even in a vacuum, which makes it play a crucial role in the heat dissipation of certain specialized electronic devices (e.g., spacecraft and space stations).^[^ ^92^ ^]^ The contribution of thermal radiation is less than heat conduction and convection under normal temperatures, while its role cannot be ignored in high‐temperature or other special situations.^[^ ^93^, ^94^ ^]^ The incorporation of micro/nanostructured surfaces into thermal radiation has revealed new regimes that deviate from classical theory.^[^ ^95^ ^]^ Traditionally, Kirchhoff's law of thermal radiation states the balance between spectral‐directional emissivity and absorptivity at thermal equilibrium (i.e., *ε=α*), indicating a reciprocal heat transfer between two bodies at different temperatures.^[^ ^96^ ^]^ However, the introduction of periodic micro/nanostructured arrays combined with materials exhibiting magneto‐optical (MO) or time‐modulated properties enables nonreciprocal thermal radiation (NTR), known as asymmetric or directional thermal emission of electromagnetic waves. The asymmetry enhances specific radiation depending on the directional energy flow. A typical case is that MO gratings under an external magnetic field can lift the degeneracy of surface plasmon‐polaritons or phonon‐polaritons, resulting in a net preferential thermal flux.^[^ ^97^ ^]^ Furthermore, selective enhancement of near‐ and far‐fields radiative modes can be achieved by tailoring the periodicity and size of the micro/nanostructures, offering great potential for numerous heat flow control and energy‐harvesting devices.^[^ ^98^, ^99^, ^100^, ^101^, ^102^, ^103^ ^]^

**Figure 4:**
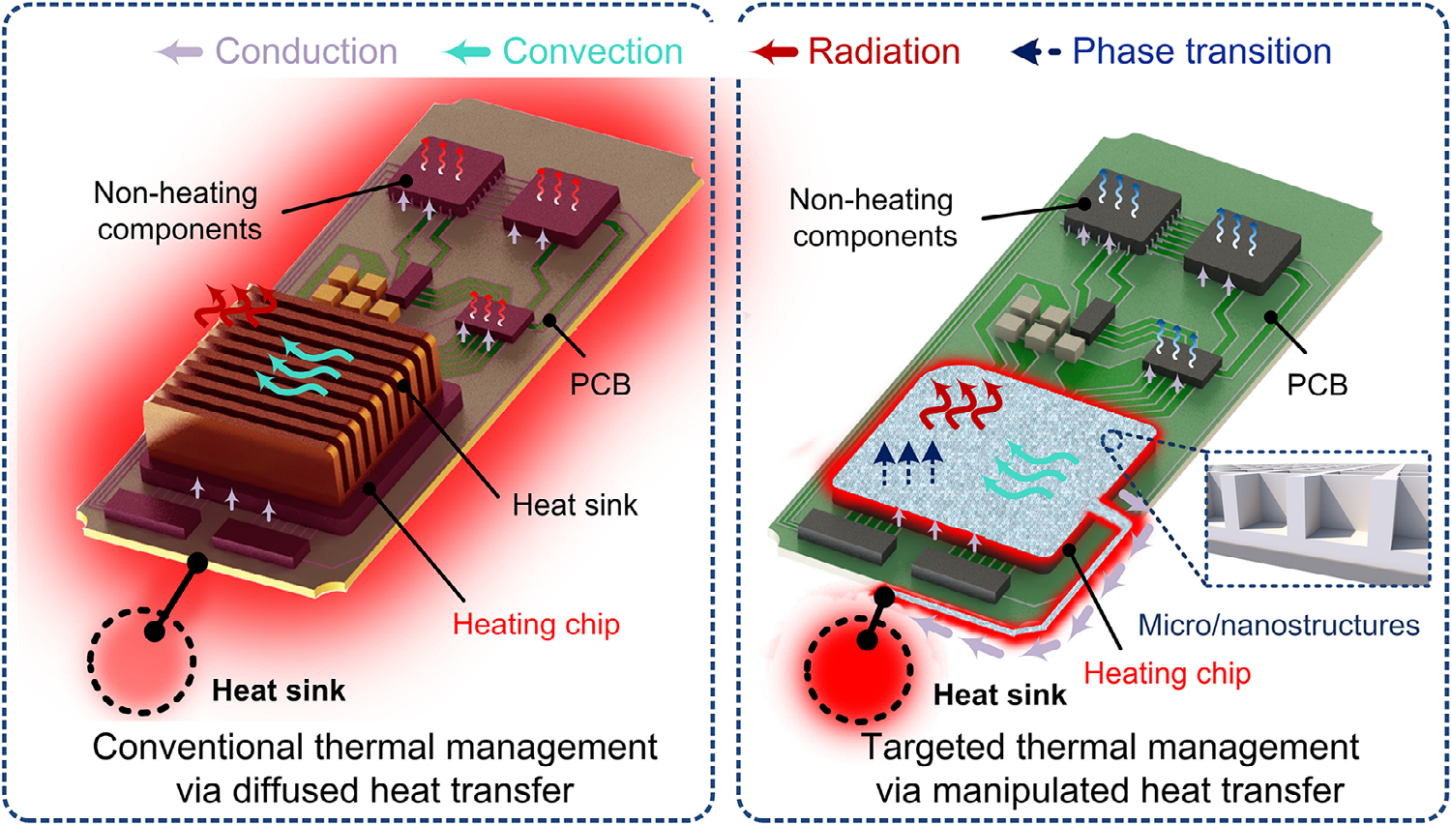
Comparison between conventional thermal management (Left) and targeted thermal management via micro/nanostructures (Right) for a typical electronic system.

Micro/nanostructures can revolutionize heat transfer mechanisms by enabling targeted thermal management (**Figure** **4**). For traditional electronic cooling methods, except for those transferred to PCBs through packaging, heat from heating electronic components is mainly dissipated by the heat sinks, typically constructed from highly thermally conductive metals with large surface areas to maximize heat spreading efficiency. Furthermore, a significant amount of heat will be dispersed throughout the equipment enclosure via radiation and convection, warming up the device structures and other less‐heating components. Then, the device dissipates heat to the external environment through a combination of convection and radiation. However, micro/nanostructured technologies enable targeted heat transfer, which manipulates heat flow pathways directly at the board and system levels, and ultimately to the outside through convection and, particularly, thermal radiation. The unique benefits include 1) efficient heat extraction to prevent overheating, 2) protecting other components by avoiding heat diffusion, 3) enhancing convection and radiation efficiency by increasing the temperature gradient, and 4) adaptive temperature control by specially designed micro/nanostructures rather than only cooling.

### 2.3 Heat Transfer Mechanism of Multi‐Level Devices

Thermal management in electronic devices is a crucial factor that guarantees their reliable operation. It begins with heat generation within chips or other heat source modules, which produces substantial heat during transistor switching operations for instruction execution. Chip‐level cooling remains a major bottleneck since local hot spots exhibit large on‐chip temperature gradients and spatial constraints.^[^ ^18^, ^26^ ^]^ A typical chip package includes an integrated heat spreader (IHS), TIM, chip die, thermoelectric coolers, and substrate, as schematically illustrated in Figure 2a. The heat generated from the chip can be dissipated through TIM to IHS in terms of thermal conduction and further to heat sinks. In addition, a small part of the heat will be conducted to the PCB via the substrate (e.g., Si, sapphire, SiC).^[^ ^25^ ^]^ Therefore, it is urgent to develop highly conductive materials^[^ ^104^ ^]^ and advanced bonding technologies,^[^ ^105^, ^106^, ^107^ ^]^ such as surface‐activated bonding,^[^ ^108^, ^109^, ^110^, ^111^, ^112^ ^]^ ion‐cutting,^[^ ^107^, ^113^, ^114^, ^115^, ^116^ ^]^ chemical bonding,^[^ ^117^, ^118^, ^119^ ^]^ and plasma bonding.^[^ ^120^, ^121^, ^122^, ^123^, ^124^ ^]^

The high power and integration of chips pose severe challenges to their thermal management. The former refers to the ongoing miniaturization of chips and the growing complexity of packaging technologies.^[^ ^125^, ^126^ ^]^ The latter encompasses the soaring peak power of graphics processing units (GPUs) and tensor processing units (TPUs), as well as the substantial energy consumption caused by model training. A prime example is that the miniaturization of chips from 14 to 3 nm has led to a 5–8 times increase in transistor density, resulting in a thermal flux density of up to 300 Wcm^−2^ (Intel 13th Gen Core processors).^[^ ^127^, ^128^ ^]^ In addition, 3D packaging technologies, such as Foveros, have enabled chip stacking with thicknesses ranging from 100‐300µm, obstructing vertical heat transfer and increasing interlayer thermal resistance by 40%–60%.^[^ ^125^ ^]^ The working temperature of the electronic chip will sharply rise to the critical temperature under such high‐power conditions,^[^ ^129^, ^130^ ^]^ resulting in a halving of chip reliability for every 10 °C increase in operating temperature.^[^ ^17^ ^]^ Furthermore, continuous overheating may lead to system crash or freeze, random failure, shortened lifespan, and safety hazards (fire risk or damage to surrounding devices). It should be clarified that central processing units (CPUs) are no longer the only heat source in microelectronic devices. GPU chips and other electronics, such as Insulated‐gate bipolar transistors (IGBTs) and metal‐oxide‐semiconductor field‐effect transistors (MOSFETs), have also been known for their escalating power densities.^[^ ^131^ ^]^ For instance, along with the explosive development of AI hardware, the thermal design power (TDP) of a single NVIDIA H100 GPU reaches 700 W, while the power consumption of the entire DGX H100 system exceeds 10 kW.^[^ ^21^ ^]^ Besides, components like power amplifiers^[^ ^132^, ^133^ ^]^ and power conversion modules^[^ ^134^, ^135^ ^]^ can be the primary heat source rather than transistors in electronic devices with special purposes, such as high‐power communication base station equipment or industrial control equipment, which are grouped into chip‐level thermal management scenarios due to their similar heat dissipation mechanisms. Notably, during the training of large models (e.g., GPT‐4o), the heat generated in a single training session is equivalent to the daily electricity consumption of 200 households.^[^ ^126^ ^]^

At the board level, sophisticated thermal management strategies are implemented to diffuse concentrated chip heat over an extended spatial domain. This process entails the conduction and diffusion of heat through PCBs, along with the convection and diffusion of heat into the ambient air, as depicted in Figure 2b. Activities at this level encompass server layouts, cabinet designs, and other factors that influence heat transfer and distribution. At the system level, the challenges lie in discharging the maximum heat to the natural environment while minimizing volume and/or mass utilization (Figure 2c). A typical device is the data center (DC), which consumes around 2% of today's world power generation, annually expanding at 12%.^[^ ^136^ ^]^ This consumption is expected to grow rapidly with the burst of artificial intelligence (AI). The primary power consumers of DCs are their cooling systems (about 40%).^[^ ^19^, ^137^, ^138^ ^]^ Hence, reducing the energy consumption of DC cooling systems through innovative thermal management strategies should be considered a high priority. On the other hand, quantum computing leverages the unique properties of quantum mechanics to solve problems that surpass the capabilities of even the most powerful classical computers, which are advancing rapidly. As quantum processors advance toward fault‐tolerant machines comprising millions of physical qubits, their power budgets will be dominated by cryogenic cooling rather than computation.^[^ ^139^, ^140^, ^141^ ^]^ For instance, despite the static thermal load at the millikelvin stage remaining on the order of microwatts, sustaining a temperature of 10–20 mK typically draws tens of kilowatts of input electrical power, which implies an annual energy consumption exceeding 100 MWh per year for 100‐qubit systems,^[^ ^142^, ^143^ ^]^ as listed in **Table** **1**.

**Table 1:** Thermal challenges of electronic devices with multi‐level computing power.

Electronic device	TDP	Heat flux density (Wcm^−2^)
Smartphone SoC^a)^	5–10 W^[^ ^144^ ^]^	15–30^[^ ^144^ ^]^
Gaming laptop CPU	45–120 W^[^ ^128^ ^]^	50–80^[^ ^128^ ^]^
Data center GPU	300–700 W^[^ ^21^ ^]^	150–300^[^ ^21^ ^]^
Quantum computer stack	20‐400 µW^[^ ^143^ ^]^ (10–25 kW^b)^ ^[^ ^140^ ^]^)	n/a

^a)^ SoC denotes system on chip.

^b)^ Continuous electrical power required to maintain a 100‐qubit system at 10 mK

The heat dissipation methods for electronic devices in different scenarios have been evaluated, as shown in **Table** **2**. The effectiveness of the three heat transfer forms strongly depends on the specific application scenarios and thermal management conditions. For instance, convection (10^4^–10^6^ Wm^−2^) and conduction (10^3^–10^6^ Wm^−2^) generally play more prominent roles than radiation (10^2^ Wm^−2^) under terrestrial conditions, as shown in the table below. However, for electronic devices in outer space (i.e., highly vacuum), where air convection is ineffective and coolant loop is not essential, releasing heat to deep space via radiation becomes a crucial thermal management method without any extra energy consumption, as recently reviewed by Fan et al.^[^ ^145^ ^]^ Combined systems (conduction, air or liquid cooling and radiation) are highly recommended. As deep space serves as an ideal cold source, radiative cooling is the inevitable pathway by which the heat dissipated from a heat source through conduction and convection is ultimately transferred into deep space. Furthermore, for high‐temperature space equipment subjected to intense solar radiation, the extreme temperatures (≈1300 °C) significantly enhance radiative heat transfer (10^5^ Wm^−2^). By integrating external radiative cooling with internal conduction and convection, redundant heat can be effectively removed, providing thermal protection to the internal components while simultaneously prolonging the service life of the insulation. Details on the calculation can be found in the Supplementary Materials. Overall, energy savings can be partially achieved through conventional thermal management strategies, such as enhancing thermal interface materials, regulating coolant flow, employing specially designed heat exchangers, and integrating thermal conversion devices. However, with the exponential growth of thermal flux demands (Table 1), innovative cooling strategies, including the combination of conductive, convective, and radiative thermal management techniques enabled by micro/nanostructure, are urgently needed at chip, board and system levels.

**Table 2:** Comparison of heat transfer performance among three modes.

Case	Conduction	Convection		Radiation
		Air	Water	
**Case 1** (Enclosed chip‐level electronics, *Computer CPU, Server, DC*)				
Heat flux density^a)^, Wm^−2^	10^6^ (TIM)	10^3^	10^5^	10^2^
Heat transfer coefficient^a)^, W(m^−2^·K^−1^)	10^5^	10^2^	10^4^	10^1^
**Case 2** (Wearable electronic devices, *Smartwatches, hearing aids*)				
Heat flux density^a)^, Wm^−2^	10^3^(Shell)	10^2^	n/a	10^2^
Heat transfer coefficient^a)^, W(m^−2^·K^−1^)	10^2^	10^1^	n/a	10^1^
**Case 3** (Outdoor rooftop/ground systems, *Building‐integrated solar cell*)				
Heat flux density^a)^, Wm^−2^	5 × 10^3^(Glass)	10^2^	10^5^	10^2^
Heat transfer coefficient^a)^, W(m^−2^·K^−1^)	10^2^	10^1^	10^4^	10^1^
**Case 4** (Satellite electronics, *High‐resolution image processors*)				
Heat flux density^a)^, Wm^−2^	10^6^ (Al)	n/a	10^5^	5 × 10^2^
Heat transfer coefficient^a)^, W(m^−2^·K^−1^)	10^4^	n/a	10^4^	10^1^
**Case 5** (High‐temperature space devices, *Parker Solar Probe*)				
Heat flux density^a)^, Wm^−2^	10^4^	n/a	5 × 10^5^	10^5^
Heat transfer coefficient^a)^, W(m^−2^·K^−1^)	10^1^	n/a	10^4^	10^2^

^a)^ The values are approximate to show their order of magnitudes.

## 3 Micro/Nanostructures for Enhanced Conduction

Theoretically, the enhancement of heat conduction relies on increasing the temperature difference, enlarging the heat exchange area, improving the effective thermal conductivity, and providing an optimal heat flow pathway.^[^ ^28^ ^]^ Inspired by the optics transformation theory established by Pendry et al.^[^ ^146^ ^]^ and Leonhardt,^[^ ^146^ ^]^ researchers have been dedicated to exploiting efficient thermal conductive controlling systems.^[^ ^147^, ^148^ ^]^ Recently, with the rapid development of advanced nanofabrication technologies, various metamaterials with micro/nanostructures have been proposed and utilized as formidable candidates for arbitrary manipulation of the distribution of heat source density, contact resistance at interfaces, and heat flow patterns, e.g., thermal cloaks, concentrators, rotators, and spreaders, arousing great interest in the scientific community.^[^ ^149^ ^]^

The specially designed nanostructures enable those metamaterial devices to fulfil threefold functions via physical rather than chemical effects, including 1) Adjustable thermal conductivity.^[^ ^150^, ^151^, ^152^, ^153^, ^154^, ^155^ ^]^ Effective thermal conductivity in specific directions of metamaterials can be manipulated according to thermal management requirements to achieve the best heat exchange effect, instead of being an inherent property for traditional materials, through the change of microstructures. 2) Thermal path regulation, e.g., thermal cloaking and camouflage.^[^ ^149^, ^156^, ^157^, ^158^, ^159^, ^160^, ^161^ ^]^ By designing and regulating thermal absorption, reflection, and transmission through micro‐/nano‐morphology, objects can present different thermal characteristics from the actual situation under thermal imaging equipment, thereby realizing the hiding of their thermal signals. 3) Anisotropic heat conduction.^[^ ^162^, ^163^, ^164^ ^]^ Different from the situation where the direction of heat flow is consistent with the temperature gradient, unconventional heat flow in specific directions can be achieved.

### 3.1 Adjustable Thermal Conductivity

Thermal conductivity modulation by engineering micro/nanostructures, and ultimately by atomic‐scale design, has been demonstrated theoretically over the last two decades. As one of the pioneering studies, Yao et al.^[^ ^165^ ^]^ employed equilibrium molecular dynamics (EMD) to quantify the length dependence of thermal conductivity in CNTs in 2005. Subsequent studies have further shown that nanostructures with geometric or mass asymmetry enable tunable thermal conductivity in low‐dimensional systems, such as nanocones,^[^ ^166^ ^]^ nanowires,^[^ ^167^ ^]^ and graphene nanoribbons (GNRs)^[^ ^39^, ^168^ ^]^ due to their ideal heat conductivity and phonon wave guide properties.^[^ ^169^, ^170^ ^]^ The TR effect of thermal conductivity has been further enhanced in recent years through advances in research methods and materials. Liang and Wei^[^ ^171^ ^]^ theoretically studied the thermal rectification (TR) in double‐layered graphene‐carbon nanotube (DGN‐DWCNT) junctions, which exhibited a significant TR effect under both large and small thermal biases, with a rectification ratio of 300.6%. Inspired by the electronic circuit, Hu et al.^[^ ^172^ ^]^ proposed a series circuit of thermal rectifiers to enhance the temperature‐dependent TR performance. By constructing an asymmetric graphene/graphene phononic crystal (GPnC) structure and performing NEMD simulations, the series effect and size effect of TR were demonstrated. Wang et al.^[^ ^173^ ^]^ experimentally delinated TR in various asymmetric monolayer graphene nanostructures. A high thermal conductivity (up to 26%) was measured in a defect‐engineered monolayer graphene with nanopores on one side compared to the region without pores. Furthermore, the TR effect endorsed by surface functionalization was widely acknowledged since it facilitates length‐insensitive thermal diodes. Li et al.^[^ ^153^ ^]^ theoretically explored the thermal transport of graphene nanoribbons with surface hydrogenation using reverse NEMD (RNEMD) simulations. The results revealed that the interface conductance strongly depends on nanostructure length, chirality, and initial temperature. Remarkable TR was observed in short nanoribbons with a higher thermal conductivity when the heat was transferred conventionally, while gradient hydrogenation can eliminate the dependency on the length. Shavikloo and Kimiagar^[^ ^154^ ^]^ studied TR in the partially hydrogenated graphene sheet with a grain boundary. A temperature gap was found at the grain boundary line due to the boundary and Kapitza resistance, and the TR decreases with the increase of the temperature difference between the hot and cold regions.

**Figure 5:**
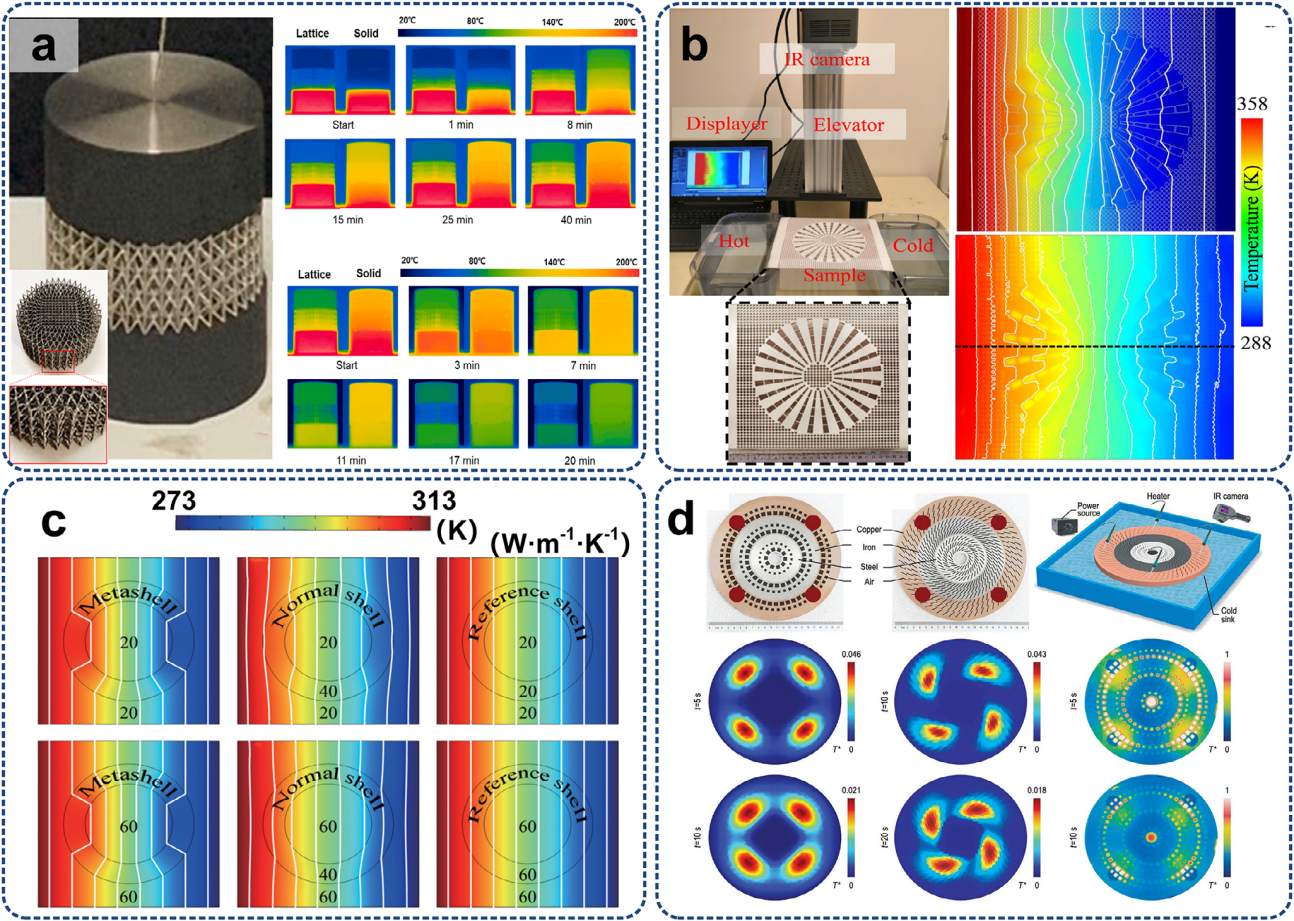
a) Photo of two thermal trapping samples with specially designed microstructures and their validated numerical simulations of temperature profiles. Reproduced with permission.^[^ ^160^ ^]^ Copyright 2022, Elsevier. b) Experimental system of thermal metadevices with simulated and measured temperature distributions. Reproduced with permission.^[^ ^183^ ^]^ Copyright 2021, Elsevier. c) Finite‐element simulations of chameleon‐like thermal metashell, normal shell, and reference shells with different thermal conductivities. Reproduced with permission.^[^ ^159^ ^]^ Copyright 2019, American Physical Society. d) Heat conduction test of the cellular cylinder with sandwich structure: Stepwise temperature distributions during the heating stage. Reproduced with permission.^[^ ^152^ ^]^ Copyright 2022, Oxford University Press.

Various 2D materials have been explored to enhance TR effects in the last decade, such as C_3_N, C_3_B, borophene, and phosphorene.^[^ ^174^, ^175^, ^176^ ^]^ Kiani et al.^[^ ^174^ ^]^ focused on the interfacial phonon transport across gold and CNT composite interface bonded by a carbon chain‐based single‐molecule junction using NEMD. The interfacial thermal conductance depended on the length of the molecules, indicating a pattern of ballistic‐diffusive phonon transport. Chen et al.^[^ ^176^ ^]^ investigated the thermal transport across the carbon/boron nitride hetero‐nanotubes (CBNNTs) interface via NEMD. The heat flows preferentially transfer from the CBNNTs to the CNTs region, demonstrating pronounced TR characteristics. Yang et al.^[^ ^177^ ^]^ investigated the thermal properties of triangle nitrogen‐doped graphene nanoribbons (TNGNs) using NEMD. It is shown that nitrogen atoms at the edge of the defect increase their thermal conductivity, while higher nitrogen‐doped concentrations lead to a sharp decrease. In addition to theoretical and numerical simulation works, some studies provided explicit examples of employing metamaterials in practical cases.^[^ ^148^, ^178^, ^179^ ^]^ Chen et al.^[^ ^178^ ^]^ present a solid‐state thermal metamaterial capable of continuously tuning thermal conductivity anisotropy via mechanically rotatable unit cells based on transformation thermotics. Built from commercially available composites, the device seamlessly integrates thermal cloaking, concentration, and rotation functions within a compact configuration. Experimental validations in battery thermal management underscore its promise for thermal control in electronics and energy systems. Given that traditional approaches are limited by the intrinsic coupling of heat flux and temperature distributions on the material, Liu et al.^[^ ^180^ ^]^ designed and developed a coordinate transformation‐based design framework for independent control of two fields, enabling dual‐functional thermal behaviors. Macroscopically, He et al.^[^ ^181^ ^]^ demonstrated macroscopic thermal rectification in bulk metamaterials using modularly assembled boron nitride microplatelet composites. By magnetically programming unit blocks into tilted orientations and bonding them into convergent/divergent states, heat flow becomes asymmetric. This single‐material, microstructural design enabled scalable thermal diodes without complex fabrication or temperature limitations. You et al.^[^ ^152^ ^]^ manipulated the thermophysical property of micro‐architectures by special lattice structures, as depicted in **Figure** **5**a, demonstrating both high insulation and cooling capability as cooler. Guo et al.^[^ ^182^ ^]^ combined passive ultra‐conductive metamaterials (AlSi10Mg) with block‐shaped natural materials to demonstrate an excellent thermal conductivity of 1915 W(m·K)^−1^ without extra energy cost, where the local thermal resistance is regulated by vertical thermal transport channels. In summary, thermal conductivity regulation via nanotechnology can optimize the heat flow pathways, featuring enhanced phonon transport efficiency, reduced interfacial thermal resistance, lightweight and flexible properties, increased heat exchanging area, and high compatibility with nanodevices. These characteristics provide significant potential for improving on‐chip thermal management.

### 3.2 Thermal Path Regulation

Thermal path regulation (e.g., thermal focusing, spreading, and trapping) has garnered immense attention in energy management, which is still challenging due to the inherent diffusive nature of heat. In 2008, Fan et al.^[^ ^150^ ^]^ employed a coordinate transformation method to develop a new type of thermal metamaterial, which enables heat flow around an “invisible” region under steady‐state conditions, termed as thermal cloaking. Meanwhile, Chen et al.^[^ ^184^ ^]^ explored the possibility of cloaking a region in curvilinearly anisotropic background metamaterials, characterized by constant properties in specific curvilinear coordinates. They demonstrated that the coordinate transformation procedure remains applicable even for cylindrically and spherically anisotropic solids, where the cloak center and material coordinate origin are generally non‐collocated. In addition to the steady‐state thermal cloaking, theoretical conceptions of unsteady‐state thermal cloaks and their extensions have also been experimentally demonstrated.^[^ ^157^, ^158^, ^185^, ^186^ ^]^ Afterwards, Li et al.^[^ ^187^ ^]^ introduced twisted thermotics, a novel approach inspired by twistronics, to control heat diffusion in bilayer thermal metamaterials. By twisting stripe‐patterned layers, the researchers experimentally demonstrated a thermal magic angle for the dynamic switching between thermal cloaking and concentration. Shen et al.^[^ ^188^, ^189^ ^]^ presented theoretical and experimental studies on a novel thermal concentrator. Contrary to the classical Fourier's law, this thermal concentrator enables heat to propagate along predetermined paths convergently by adjusting the microstructures. As illustrated in Figure 5b, the heat flux converges to a focal point on the interface, distinct from conventional heat conduction patterns.^[^ ^183^ ^]^ Hamed and Ndao^[^ ^190^ ^]^ developed a high‐anisotropy metamaterial heat spreader, which consists of alternating bilayers of copper/ultra‐high temperature ceramic (UHTC) and polydimethylsiloxane (PDMS) thin films. This device significantly enhanced lateral heat spreading compared to the simple copper heat sink. Similarly, Bai et al.^[^ ^149^ ^]^ focused on designing a thermal metamaterial‐based heat spreader to protect electronic device elements from heat source impacts. The density model in COMSOL's topological optimization was harnessed to predict the optimal material distribution of carbon fiber film and paraffin, achieving a 5.92 K temperature reduction with optimized parameters. Russell et al.^[^ ^161^ ^]^ designed a class of spatially anisotropy metamaterial heat spreaders with conical and trapezoidal shapes. A ground cloak transformation was modified to utilize the mapping in a new heat‐spreading manner. Xu et al.^[^ ^159^ ^]^ found novel passive metashells to realize chameleonlike behavior with adaptive thermal conductivities. The heat flow can be adjusted without being affected by densities or heat capacities (Figure 5c). They recently developed black‐hole‐inspired meta‐devices to achieve thermal trapping via graded heat conduction based on a theoretical framework incorporating hybrid transformations,^[^ ^160^ ^]^ as shown in Figure 5d. In general, thermal path regulation by micro/nanostructured metamaterials facilitates heat flow to pre‐designed locations for directional heat exchanging, which thereby prevents redundant heating of the board‐level devices (i.e., PCB).

### 3.3 Anisotropic Heat Conduction

Removing basic assumptions proposed in the Onsager–Casimir theory facilitates the development of nonreciprocal technology.^[^ ^99^ ^]^ Over the past two decades, substantial effort has been devoted to developing nonreciprocity based on nonlinear materials featuring space‐dependent or time‐dependent constitutive properties.^[^ ^191^ ^]^ The possible thermal diode was first introduced by Li et al.^[^ ^147^ ^]^ in 2004, a numerical model was exhibited that exhibited directional heat flow manipulation by using two coupled nonlinear lattices. The effect can be attributed to asymmetrical interfacial thermal resistance according to their qualitative analysis based on numerical results.^[^ ^192^ ^]^ However, as the chain length of the FK model increases, the anisotropic effect diminishes with the attenuation of nonlinearity.^[^ ^193^ ^]^ This issue can be mitigated promptly using separated structures (a ballistic channel connected to a single mass‐graded nonlinear chain),^[^ ^194^ ^]^ while the underlying problem of size‐dependent thermal rectification remains unresolved. However, a localized thermal diode with a high rectification factor (>10^6^) was achieved by introducing a finite‐depth defect and asymmetric coupling, and the size‐dependent effect was alleviated.^[^ ^195^ ^]^

**Figure 6:**
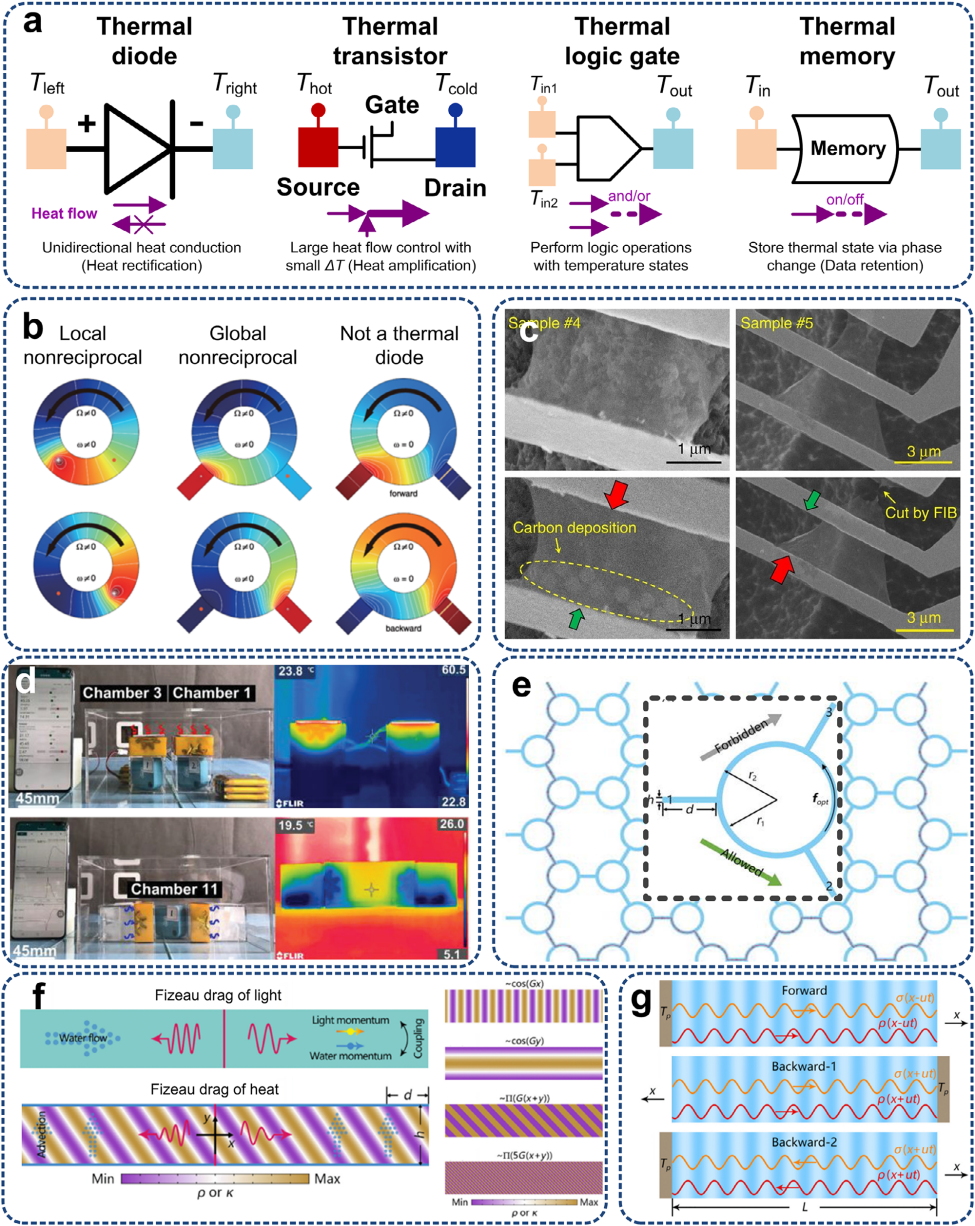
a) Schematics of various thermal phononic devices, including thermal diode, thermal transistor, thermal logic gate, and thermal memory. The heat flux distribution indicated by the purple arrows illustrates functionalities such as unidirectional heat conduction, heat flux amplification, logic computation, and data storage. b) Temperature profiles on three rotating ring configurations generated by a time‐harmonic point heat source with all boundaries thermally insulated. Reproduced with permission.^[^ ^102^ ^]^ Copyright 2022, American Physical Society. c) SEM images of graphene‐based thermal diode, where large red and small green arrows represent heat flow direction with high thermal conduction and opposite direction with low conduction, respectively. Reproduced with permission.^[^ ^173^ ^]^ Copyright 2017, Springer Nature. d) Experiment device and infrared images of temperature chambers with different thermal diode arrangements. Reproduced with permission.^[^ ^213^ ^]^ Copyright 2021, Wiley‐VCH. e) A Schematic diagram of angular‐momentum‐biased ring exhibiting thermal nonreciprocity and isolation. Reproduced with permission.^[^ ^223^ ^]^ Copyright 2021, AIP Publishing. f) Origin of diffusive Fizeau drag: heat in a spatiotemporal thermal metamaterial by thermal Willis coupling. Reproduced with permission.^[^ ^164^ ^]^ Copyright 2021, American Physical Society. g) Schematics of thermal wave nonreciprocity, including forward case, backward‐1 case by changing the source position, and backward‐2 case by changing the modulation direction. Reproduced with permission.^[^ ^214^ ^]^ Copyright 2021, American Physical Society.

Furthermore, as reviewed by Li et al.,^[^ ^196^ ^]^ various phononic devices including thermal diodes,^[^ ^102^ ^]^ thermal transistors,^[^ ^197^, ^198^, ^199^, ^200^ ^]^ thermal logic gates,^[^ ^201^, ^202^ ^]^ and thermal memory^[^ ^203^ ^]^ have been theoretically designed and developed, leveraging nonlinear dynamics and symmetry breaking to enable functionalities like rectification, amplification, and heat flow switching. **Figure** **6**a schematically explicates the function of these phononic devices. Analogous to electronic components, thermal diodes enable the forward flow of heat while impeding its reverse flow. Thermal transistors, on the other hand, amplify heat flow through the temperature difference from the gate. Thermal logic gates can implement Boolean logic using an array of thermal diodes, with a significant application in thermal calculators. Thermal memory can achieve data access and retention by reading temperature‐related information. In addition, inspired by the classic thermal diode (Figure 6b), thermal control technologies, including photonic thermal diode,^[^ ^204^ ^]^ electronic thermal diode,^[^ ^205^ ^]^ topological phononic diode,^[^ ^206^ ^]^ acoustic diode,^[^ ^207^ ^]^ radiation thermal diode,^[^ ^103^ ^]^ and elastic energy diode^[^ ^208^ ^]^ have been accordingly extended.^[^ ^209^ ^]^ These emerging technologies exhibit promising functionalities across different regimes in electronic thermal management. For example, photonic thermal diodes have been explored for unidirectional thermal radiation transport, which is further delineated in Section 6.3. Electronic thermal diodes leverage electron transport mechanisms for directional conduction in high‐power devices, while elastic energy diodes present opportunities for integrated control of mechanical and thermal transport in flexible electronics. Emerging directions in phononics span from topological phononics, which exploits band topology to enable robust transport and thermal regulation, to quantum phononics, which harnesses phonon quantum states and coherence for next‐generation quantum information technologies.^[^ ^206^, ^210^ ^]^ They have been analytically, numerically, and experimentally explored, with tremendous applicable potential in complex thermal and energy management, e.g., quantum heat engines and quantum refrigerators.^[^ ^211^, ^212^ ^]^

Pioneer experimental work has demonstrated thermal rectification in mass‐loading nanotubes obtained via vapor deposition of trimethyl‐cyclopentadienyl platinum (C_9_H_16_Pt),^[^ ^197^ ^]^ where the effect was attributed to solitons. Afterwards, advancements in experimental validation of thermal diodes span a broad range of scales^[^ ^213^, ^215^, ^216^, ^217^, ^218^, ^219^ ^]^ (Figure 6c,d). Microscopic systems such as defect‐engineered graphene nanostructures can achieve a thermal rectification factor of 26% (the ratio between heat flux enhancement and that before the rectification).^[^ ^173^ ^]^ Macroscopic devices, such as shape‐memory alloy‐based thermal diodes, have demonstrated switchable heat conduction through temperature‐dependent structural transformations.^[^ ^215^ ^]^ Xie et al.^[^ ^220^ ^]^ realized a solid‐state thermal memory using a VO_2_ nanobeam by metal‐insulator transitions. However, challenges ahead involve unifying performance metrics, developing advanced measurement techniques, device miniaturization, and addressing thermodynamic constraints.^[^ ^43^, ^185^, ^221^, ^222^ ^]^

Furthermore, the difference in temperature field diffused in opposite directions has also been achieved, considering the difference in light travel resistance across various media.^[^ ^214^, ^223^, ^224^, ^225^, ^226^ ^]^ Xu et al.^[^ ^163^ ^]^ revealed that robust one‐way edge states can exist in convection‐diffusion systems, as schematically shown in Figure 6f. Convection breaks space‐reversal symmetry and contributes to one‐way temperature propagation, similar to the role of electron cyclotrons in thermal edge propagation. The same team fabricated a spatiotemporal thermal metamaterial to reveal Fizeau drag in thermal propagation,^[^ ^164^ ^]^ as depicted in Figure 6g. The space‐related inhomogeneity and time‐related advection enabled the diffusive Fizeau drag effect, resulting in different propagating speeds of temperature fields in opposite directions. Furthermore, by introducing an angular‐momentum bias (thermal Zeeman effect) via a volume force, they extrapolated the electronic and acoustic Zeeman effect in convection‐diffusion thermal systems.^[^ ^223^ ^]^ Temperature modes split and interfere to produce nonreciprocal propagation and isolation in a three‐port ring can be achieved without relying on temperature‐dependent or phase‐change materials, as illustrated in Figure 6e. Meanwhile, thermal nonreciprocity based on spatiotemporal modulation was fundamentally discussed,^[^ ^214^ ^]^ which demonstrated a flexible and tunable approach to controlling thermal nonreciprocity using a phase difference.

It can be concluded that recent advances in thermal rectification highlighted the potential of micro/nanostructures to overcome the diffusive nature of heat transport. Theoretically, atomically engineered junctions have achieved high rectification ratios, while surface hydrogenation and heterostructure design enable length‐insensitive or directionally biased heat transfer. Experimental investigations into anisotropic thermal conduction span a broad range of scales, which demonstrated distinct differences in thermal conduction using defect‐engineered nanostructures. However, challenges remain in device scalability since the rectification effects degrade with system size. Experimental characterization and complicated comparisons across material systems are still lacking. Therefore, future research may focus on developing design principles that unify atomic‐level phonon engineering with macroscale metamaterial architectures, thereby enabling robust rectification independent of size and operating temperature. Meanwhile, integration of high‐conductivity metamaterials with chip‐scale devices could provide practical thermal diodes for next‐generation electronics, which demand not only materials innovation but also advanced measurement tools to resolve heat flow at nanometer and femtosecond scales.

## 4 Micro/Nanostructures for Enhanced Single‐Phase Convection

### 4.1 Flow Pattern Manipulation

Hydrodynamic designs with micro/nanostructures that can accelerate single‐phase flow in specific directions or regions have been proposed to effectively reduce energy losses along the flow path and enhance heat dissipation (e.g., coolant loop). Particular attention has been devoted to the flow direction, path, and velocity of those systems. Precise fluid manipulation can be achieved by delicate engineering micro/nanostructures, resulting in multiple applications tailored for prescribed manners, including hydrodynamic diodes,^[^ ^227^, ^228^, ^229^ ^]^ cloaks,^[^ ^230^, ^231^, ^232^ ^]^ concentrators,^[^ ^233^ ^]^ rotators,^[^ ^234^, ^235^ ^]^ and eveners.^[^ ^236^, ^237^, ^238^ ^]^ Two principal approaches dominate the design of hydrodynamic metadevices, namely transformation mapping theory^[^ ^146^, ^233^, ^239^ ^]^ and scattering cancellation theory,^[^ ^157^, ^238^, ^240^ ^]^ as previously discussed by Chen et al.^[^ ^241^ ^]^ The former exploits coordinate transformations to preserve the form invariance of governing equations, enabling multifunctional flow manipulation (e.g., cloaking, concentration, and rotation) through spatially anisotropic permeability tensors.^[^ ^235^ ^]^ Notably, permeability in different functions can be mathematically described in a similar form for fluid flow governed by the Laplace theory, indicating that these functions can be integrated into a single hydrodynamic metadevice.^[^ ^241^, ^242^ ^]^ While transformation methods enable versatile functionalities, they typically require complex micro/nanostructures with extreme parameter gradients, which compromise practical fabrication.^[^ ^243^, ^244^, ^245^ ^]^ In contrast, scattering cancellation theory achieves cloaking via homogeneous isotropic layers that annihilate scattering fields, offering manufacturable designs.^[^ ^157^, ^240^, ^246^ ^]^ However, its functionality is primarily limited to hydrodynamic cloaking and requires specific background materials.^[^ ^246^ ^]^ In summary, transformation‐based devices excel in programmable multi‐functionality, but face constraints in flow states, material properties, and manufacturing.^[^ ^230^ ^]^ In contrast, scattering‐cancellation systems prioritize experimental feasibility for practical flow manipulation devices. Emerging hybrid strategies may synergize their strengths for adaptive, large‐scale, precise flow manipulation (see^[^ ^242^ ^]^ for details).

**Figure 7:**
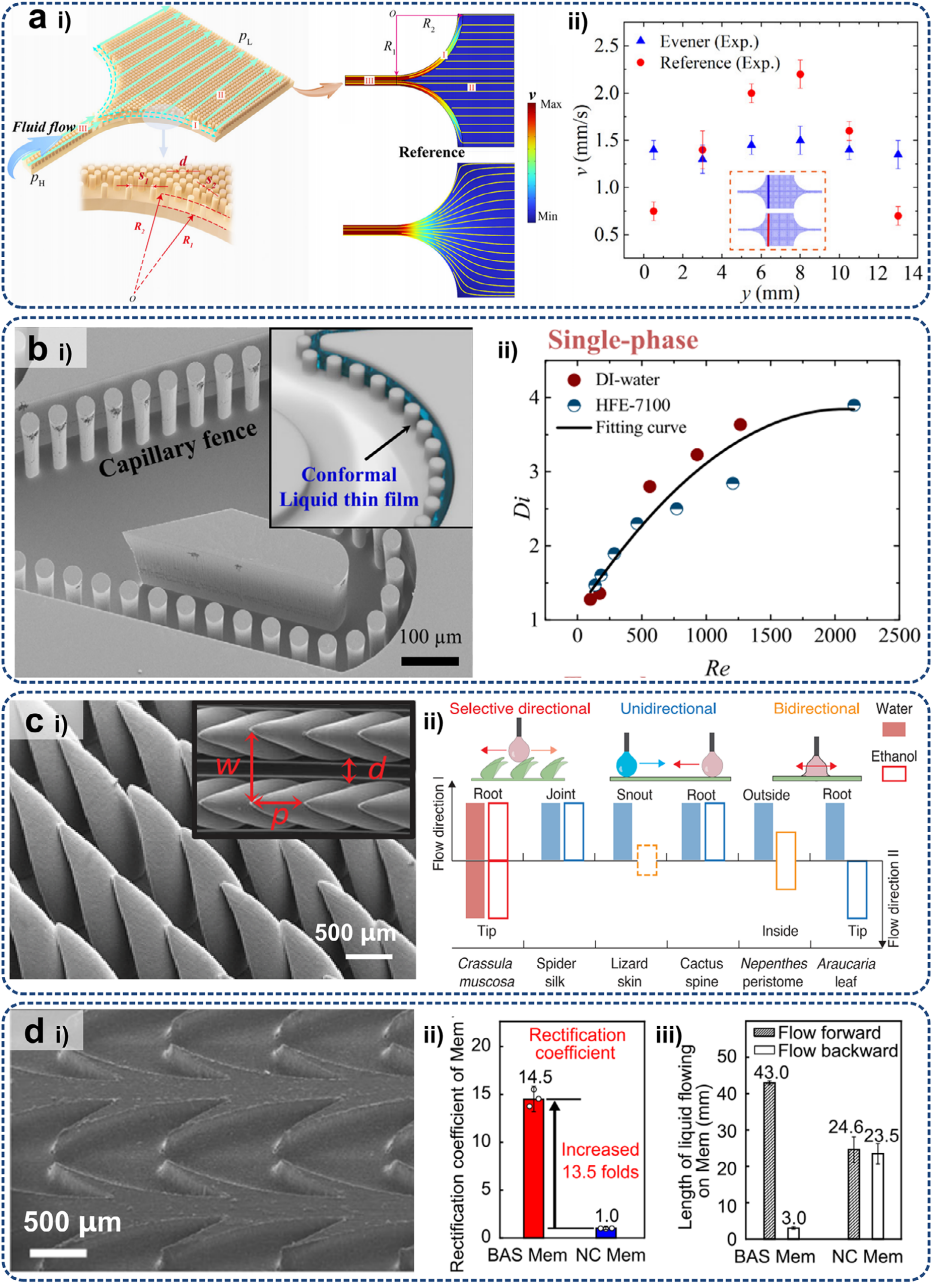
a‐i) Schematic of the specific structure design of a hydrodynamic evener consists of pillar arrays and its reference. a‐ii) Experimental validation of the flow uniformity obtained from the new evener compared to its reference. Reproduced with permission.^[^ ^238^ ^]^ Copyright 2024, AIP Publishing. b‐i) SEM images of a thermal regulator, including Tesla valves and regularly patterned pillars on the sidewalls. b‐ii) The variation of diodicity with *Re* and effective heat flux of one‐phase flow (DI‐water and HFE‐7000). Reproduced with permission.^[^ ^247^ ^]^ Copyright 2023, Springer Nature. c‐i) Microstructures of ALIS. The row‐to‐row width *w*, interside‐to‐interside width *d*, and tip‐to‐tip pitch *p* are 1000, 400, and 750 µm, respectively. c‐ii) Comparison of different transport modes of water and ethanol in different species. Reproduced with permission.^[^ ^228^ ^]^ Copyright 2024, AAAS. d‐i) Magnified SEM image of the Barbed arrow‐like structure. d‐ii) Rectification coefficients comparison between two types of membranes. d‐iii) Flow distance comparison of two types of membranes. Reproduced with permission.^[^ ^248^ ^]^ Copyright 2023, Springer Nature.

Some typical metadevices are expected to inspire the development of optimal resolutions for electronic heat dissipation at micro/nanoscales. Aiming at maintaining uniform water flow for optimizing liquid transport efficiency in medical and thermal management applications, Chen et al.^[^ ^238^ ^]^ proposed a porous hydrodynamic evener based on scattering cancellation theory, as shown in **Figure** **7**a. Experimental and numerical studies have validated its capability to maintain uniform water streamlines and velocity distributions in the presence of channel expansions or constrictions in the presented hydrodynamic metadevice. The extension of this design from 2D to 3D configurations highlighted its potential for manipulating large‐scale liquid flows. Park et al.^[^ ^234^ ^]^ developed a fluid‐flow rotator with anisotropic micropillars using the coordinate transformations method, which derives a spatially constant viscosity tensor to twist fluidic coordinate systems. Numerical simulations demonstrated their ability to redirect flow by varying angles (180°, 90°, and 30°) through the analysis of pressure and velocity fields, while the experimental realization confirmed a 30° flow rotation. Furthermore, they demonstrated a novel metamaterial hydrodynamic flow concentrator^[^ ^233^ ^]^ via transformation physics, engineered to focus fluid flow into a target region by leveraging coordinate transformations of the Stokes equations (low‐*Re*, incompressible, steady‐state flows). Experimentally, the visualization of fluorescent particles in the periodic micropillar concentrator demonstrated a 2.1‐fold increase in flow rate and a 4.5‐fold enhancement in hydrodynamic energy within the central target area, without disturbing the surrounding flow.

The development of hydrodynamic diodes is crucial for enhancing the performance of electronic thermal management systems at micro/nanoscales, particularly passive two‐phase loop flow systems, since they facilitate directional fluid movement without extra energy. Li et al.^[^ ^247^ ^]^ developed a novel capillary thermal regulator inspired by the Tesla valve to mitigate the heat transport deterioration caused by vapor backflow and chaotic two‐phase flow pattern, as shown in Figure 7b. It is found that the capillary strategy promotes liquid flow along sidewalls, avoiding dry‐out and enabling stable and continuous flow, which led to a capillary pressure within the meniscus of over 13 times higher than rectangular microchannels (Figure 7b‐ii). Wang et al.^[^ ^227^, ^228^ ^]^ proposed biomimetic structures (Araucaria leaf–inspired surface, ALIS, and Crassula muscosa inspired‐arrays, CMIAs) to realize directional liquid transport in two directions (Figure 7c), which was accomplished by magnetically controlling the reentrant angles of leaves for the variation in liquid meniscus heterogeneity. Furthermore, Li et al.^[^ ^248^ ^]^ considered a barbed arrow‐like structure (Figure 7d‐i) to achieve ultrafast and unidirectional transport of liquid shown in Figure 7d‐ii, which allowed a rectification coefficient as high as 14.5 (Figure 7d‐ii). Recently, a capillary‐driven 3D open fluidic networks (OFNs) was developed by Wu et al.,^[^ ^249^ ^]^ composed of interconnected polyhedral frames and connecting rods, which addresses the limitations of conventional closed‐pipe systems that restrict fluid‐environment interactions. Each polyhedral frame in OFNs functions as a fluid chamber with free interfaces enabling fluid entry or exit, while connecting rods act as valves to precisely control flow direction, velocity, and path. The formation of spontaneous capillary flow in OFNs is governed by wettability (hydrophilic), dimension relative to capillary length, and energy balance, ensuring reliable operation across diverse fluid systems (e.g., water in air, oil in water). These unique features enable enhanced heat transfer performance with open interfaces, where OFNs exhibit 1.7 times higher overall heat transfer coefficient compared to traditional heat exchanging systems with solid boundaries. Overall, for chip‐ or board‐level electronics compatible with liquid cooling, optimizing flow patterns and directions through micro/nanostructures can effectively mitigate liquid backflow and stagnation. This is particularly critical for devices incorporating phase‐change cooling systems, where achieving an efficient two‐phase separation flow state is essential for optimal thermal performance.

**Figure 8:**
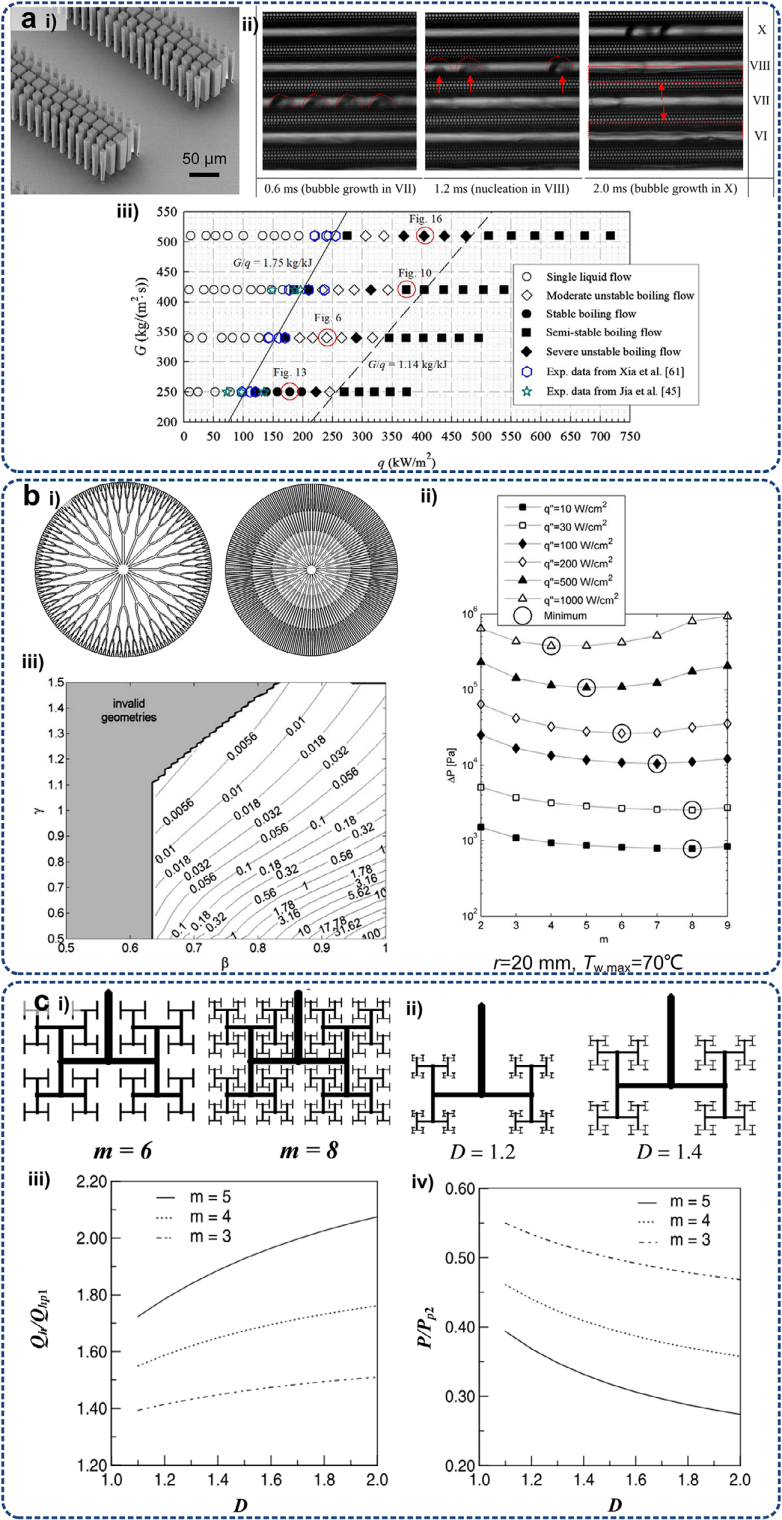
a‐i) SEM images of porous wall microchannels with micro pin fin arrangements. a‐ii) The vapor transmission phenomenon in the midstream of the porous‐wall microchannels (channel VI to X). a‐iii) Five boiling flow modes identified with the present experimental data of acetone at varying flow rates and heat flux densities induce minor oscillation with the domain frequencies of 28/30 Hz. Reproduced with permission.^[^ ^253^ ^]^ Copyright 2019, Elsevier. b‐i) Schematical diagram of flow networks with 16 branches emanating from the inlet plenum at the disk center with decreasing and increasing channel lengths. b‐ii) Minimized pressure drop changing with the number of *m* as a function of disk heat flux, where optimal *m*_b_ is highlighted by large circles. b‐iii) Representative division between valid and invalid geometries when *r*=20 mm, *w*_m_=85µm, *n*_0_=10, and *m*_b_=6. Reproduced with permission.^[^ ^257^ ^]^ Copyright 2010, Elsevier. c‐i) Schematic branch‐like microchannel profiles of different *m* at *D*=2. c‐ii) Microchannels of different fractal dimensions at *m*=7. c‐iii) The convection heat transfer ratio of fractal and parallel channels varies with channel lengths at different *m*. c‐iv) The pumping power ratio of fractal and parallel channels varies with channel lengths at different *m*. Reproduced with permission.^[^ ^258^ ^]^ Copyright 2002, Elsevier.

Microchannels were proposed in the 1980s for high‐power density electronic cooling, which has become popular in passive and active thermal management devices due to capillary and heat transfer advantages. Recently, micro/nanostructures have been applied to further improve liquid transportation and two‐phase flow stability. Woodcock et al.^[^ ^250^, ^251^ ^]^ proposed a piranha pin fin (PPF) microstructure to dissipate high heat fluxes using hydrofluoroethers (HFE7000) as a coolant. The microdevices were developed as an array of 150 µm diameter microstructures embedded within a 2.4 mm channel, featuring tailored slot‐jet orifices for vapor extraction and reentrant cavities. Xu et al.^[^ ^252^ ^]^ and Zong et al.^[^ ^253^ ^]^ developed gradient–porous–wall microchannels to prevent the bubble confinement effect and non‐uniform phase distribution, as illustrated in **Figure** **8**a‐i). The vapor flow direction is periodically switched between neighboring channels, causing the eye‐blinking interface motion to eliminate the channel blockage (Figure 8a‐ii). The evolution of five boiling modes was identified experimentally, and a minor oscillation with stable frequencies was observed (Figure 8a‐iii). To tackle the dry‐out problem shown in high aspect ratio (AR) microchannels, Wu et al.^[^ ^254^ ^]^ proposed a high AR groove‐wall microchannels featuring improved boiling behavior in the two‐phase region due to the thin liquid film evaporation in the relatively large cavity space. Typical sawtooth structures were established by Zhou et al.^[^ ^255^ ^]^ and Wongwises,^[^ ^256^ ^]^ where both the convective heat transfer area and the turbulence intensity of fluid flow were enhanced compared to the smooth channels.

The study of fractal geometry further accelerated after the pioneering work in the 1960s,^[^ ^259^ ^]^ characterized as self‐similarity branched systems. After that, scholars have found the fractal geometry inspired by elements in nature, such as bronchial trees,^[^ ^260^ ^]^ vascular systems,^[^ ^261^ ^]^ the leaf or root of the plant,^[^ ^262^ ^]^ snowflakes, and river channels.^[^ ^258^ ^]^ Based on the theoretical research by Bejan and Lorente^[^ ^263^ ^]^ and Pence,^[^ ^264^ ^]^ Heymann et al.^[^ ^257^ ^]^ optimized multiple geometric parameters on the disk‐shaped flow network by using two algorithms. Figure 8b‐i presents typical 16‐branch flow arrangements with increasing or decreasing channel lengths. The gradient‐based steepest descent search method was applied to optimize the fractal geometry, which resulted in a representative range of valid length and width ratios for fixed disk radius (*r*), terminal branch width (*w*_m_), total branch number of level 0 (*n*_0_), and branching level (*m*_b_), as shown in Figure 8b‐ii. The calculations showed that fewer branch levels with many channels emanating from the inlet plenum are suitable for higher heat flux cooling applications (Figure 8b‐iii). Du et al.^[^ ^265^ ^]^ designed four topologies of microchannels for chip cooling, i.e., ternate veiny, lateral veiny, snowflake, and spider net. By improving the uniformity of fluid distribution and flow rate, the spider‐netted structure yields better thermal performance than other cases. Chen et al.^[^ ^258^ ^]^ and Ghodoossi^[^ ^266^ ^]^ explored the incorporation of fractal branching channel nets inspired by mammalian circulatory systems in microscale device cooling systems. The H‐type channels enable rapid coverage of the entire heated surface by liquid, achieving superior heat transfer over traditional parallel channels, as depicted in Figure 8c‐i,c‐ii). This finding underscores the potential of fractal geometries to revolutionize thermal management in microscale devices. The flow and heat transfer performance of tree‐like microchannels were theoretically analyzed, which showed that the pump power and convection heat transfer increase with the branching level (Figure 8c‐iii) while decreasing with the increasing channel length (Figure 8c‐iv). Furthermore, Fan et al.^[^ ^267^ ^]^ investigated the effects of length ratio, height‐to‐width ratio, and channel volume fraction on maximum temperature and surface uniformity of the tree‐like structured heat sink.

### 4.2 Surface Wettability

**Figure 9:**
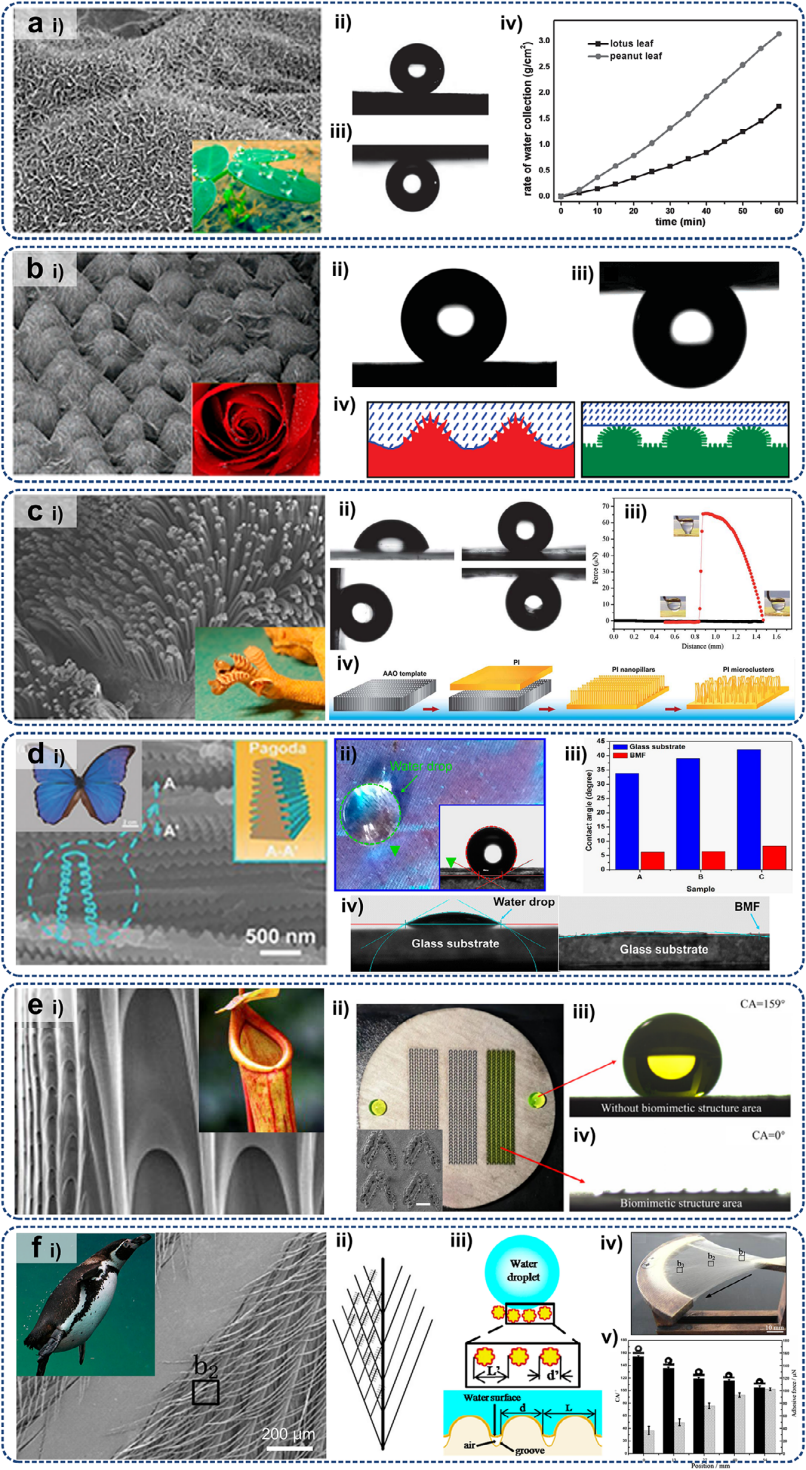
Typical bio‐inspired surface structures show excellent superhydrophobic properties. a‐i) SEM images and a photo of a peanut leaf. CA of water droplets (4µL) on the peanut leaf with the tilt angle of a‐ii) 0° and a‐iii) 180°. a‐iv) Collected water accumulation of the lotus leaf and peanut leaf with prolonged time in the fog flow. Reproduced with permission.^[^ ^277^ ^]^ Copyright 2014, American Chemical Society. b‐i) SEM image and photo of a red rose petal. Shape of a water droplet on the petal's surface with the tilt angle of b‐ii) 0° and b‐iii) 180°, indicating its super‐hydrophobicity with a contact angle of 152.4°. b‐iv) Schematic of a water droplet in contact with the petal of a red rose and a lotus leaf. Reproduced with permission.^[^ ^278^ ^]^ Copyright 2014, American Chemical Society. c‐i) SEM image and photo of the Gecko foot. c‐ii) A water droplet on the polyimide film without nanostructures, exhibiting the hydrophilic character. CA of a water droplet on the gecko‐inspired surface with different tilt angles, 0°, 90°, and 180°, demonstrating both superhydrophobicity and a high adhesive force. c‐iii) Force–distance curves recorded before and after the water droplet contacted the gecko‐inspired surface. c‐iv) Schematic representation of the manufacturing process. Reproduced with permission.^[^ ^274^, ^279^ ^]^ Copyright 2014, American Chemical Society. d‐i) SEM image and photo of the butterfly wing surface. d‐ii) Digital photo and CA measurement image show excellent superhydrophobic property. d‐iii) Contact angle comparison between glass substrate and BMF. Photos of water droplets contacting with d‐iv) untreated glass substrate and d‐v) BMF for comparison. Reproduced with permission.^[^ ^275^, ^280^ ^]^ Copyright 2018, Wiley‐VCH. e‐i) SEM image and photo of Nepenthes pitcher plant. e‐ii) Wettability characteristics of the biomimetic structure with a scale bar of 250 µm. e‐iii) The contact angle of the droplet on the biomimetic structure is 0°, indicating a super‐hydrophilic character. e‐iv) A hydrophobic nature shown without biomimetic structure. Reproduced with permission.^[^ ^272^ ^]^ Copyright 2024, AIP Publishing. f‐i) SEM image and photo of penguin feathers. f‐ii) Schematic diagram of a 3D microstructural network on the surface of the back feathers, including oriented barb, barbules, and hamuli. f‐iii) Contact between water microdroplets and hamuli to elucidate its hydrophobicity and icephobicity. f‐iv) Polyimide nanofiber membranes (Artificial replica of penguin feathers). f‐v) Water CA and adhesion force at different positions of the polyimide nanofiber membranes. Reproduced with permission.^[^ ^276^ ^]^ Copyright 2016 American Chemical Society.

Surface wettability significantly impacts boiling heat transfer, prompting numerous studies to focus on the synergy between micro/nano surface structures and wetting behaviors.^[^ ^268^ ^]^ According to the Wenzel and Cassie‐Baxter relation, surface wetting can be controlled by adjusting the surface area fraction for roughened and structured surfaces with micro/nanostructures. Therefore, the precise control of micro/nanoscale morphology is essential to achieve tunable wettability. Biomimetic studies have determined that the collaboration between these unique multi‐scale structures and the inherent properties of the material has led to the observed wettability and versatility. Examples in nature include self‐cleaning superhydrophobic surfaces of lotus leaves,^[^ ^269^ ^]^ Toro leaves, water lilies, spider webs, anisotropic superhydrophobic rice leaves, structure‐colored red rose petals,^[^ ^270^ ^]^ superhydrophobic sharkskin,^[^ ^271^ ^]^ Nepenthes pitcher lip surface,^[^ ^272^ ^]^ gecko foot,^[^ ^273^ ^]^ hydrogel‐like beetle back surface,^[^ ^274^ ^]^ butterfly wing,^[^ ^275^ ^]^ penguin feather.^[^ ^276^ ^]^ **Figure** **9**a‐i demonstrated the microscopic images of the peanut leaf surface,^[^ ^277^ ^]^ which introduced a superhydrophobic character, as shown in Figure 9a‐ii,a‐iii. The collected water accumulation of the peanut leaf is higher than that of the lotus leaf due to this feature (Figure 9a‐iv). Similar characteristics were exhibited on the surface of rose petals,^[^ ^278^ ^]^ as illustrated in Figure 9b‐i. A contact angle (CA) of 152.4° (Figure 9b‐ii) and high adhesive force (Figure 9b‐iii) indicated its advantages compared to the lotus leaf surface, whose mechanism was explained in Figure 9b‐iv. Liu et al.^[^ ^279^ ^]^ proposed a superhydrophobic gecko feet‐inspired surface (Figure 9c‐i). Photos of CA (Figure 9c‐ii) and force‐distance curves (Figure 9c‐iii) clarified its high adhesive forces towards water, and the fabrication process of polyimide films with bio‐inspired features was introduced (Figure 9c‐iv). A SiO_2_ biomimetic monolayer film (BMF) with multiscale hierarchical pagoda structures (MHPSs) on a glass base was fabricated using a biological template method, using the butterfly wing surface.^[^ ^280^ ^]^ SEM image and photo of the butterfly wing surface (Figure 9d‐i) explained its excellent superhydrophobic property, as shown in Figure 9d‐ii. The comparison between water wettability (Figure 9d‐iii, d‐iv) demonstrates its high active antifogging Performance. Inspired by the Nepenthes pitcher plant, Zhang et al.^[^ ^272^ ^]^ presented the wettability characteristics of Ti alloy substrate with biomimetic structure (Figure 9e‐i). Comparison of CA in areas with and without microstructures demonstrates its exceptional hydrophilicity and penetration capability (Figure 9e‐ii,e‐ii,e‐iv). Figure 9f‐i demonstrates a hydro‐ and icephobic surface inspired by penguin feathers. The hydrophobicity of microstructure was schematically explained by the contact mechanism between water microdroplets and hamuli, as shown in Figure 9f‐ii,f‐iii. The adhesive force and CA show opposite trends, changing with the distance of the Polyimide nanofiber membranes as an artificial replica of the feather (Figure 9f‐iv,f‐v). Wettability modification via micro/nanostructures is useful when applied in liquid cooling technologies for electronic devices. Specifically, it can be integrated with liquid metal cooling, micro‐loop heat pipes, and micro‐pump‐driven two‐phase systems to tailor the hydrophilicity or hydrophobicity of specific regions based on their requirements. By enhancing liquid‐phase wetting rates (hydrophilic surfaces) or bubble departure rates (hydrophobic surfaces), hydrodynamic and thermal characteristics can be significantly improved.

**Figure 10:**
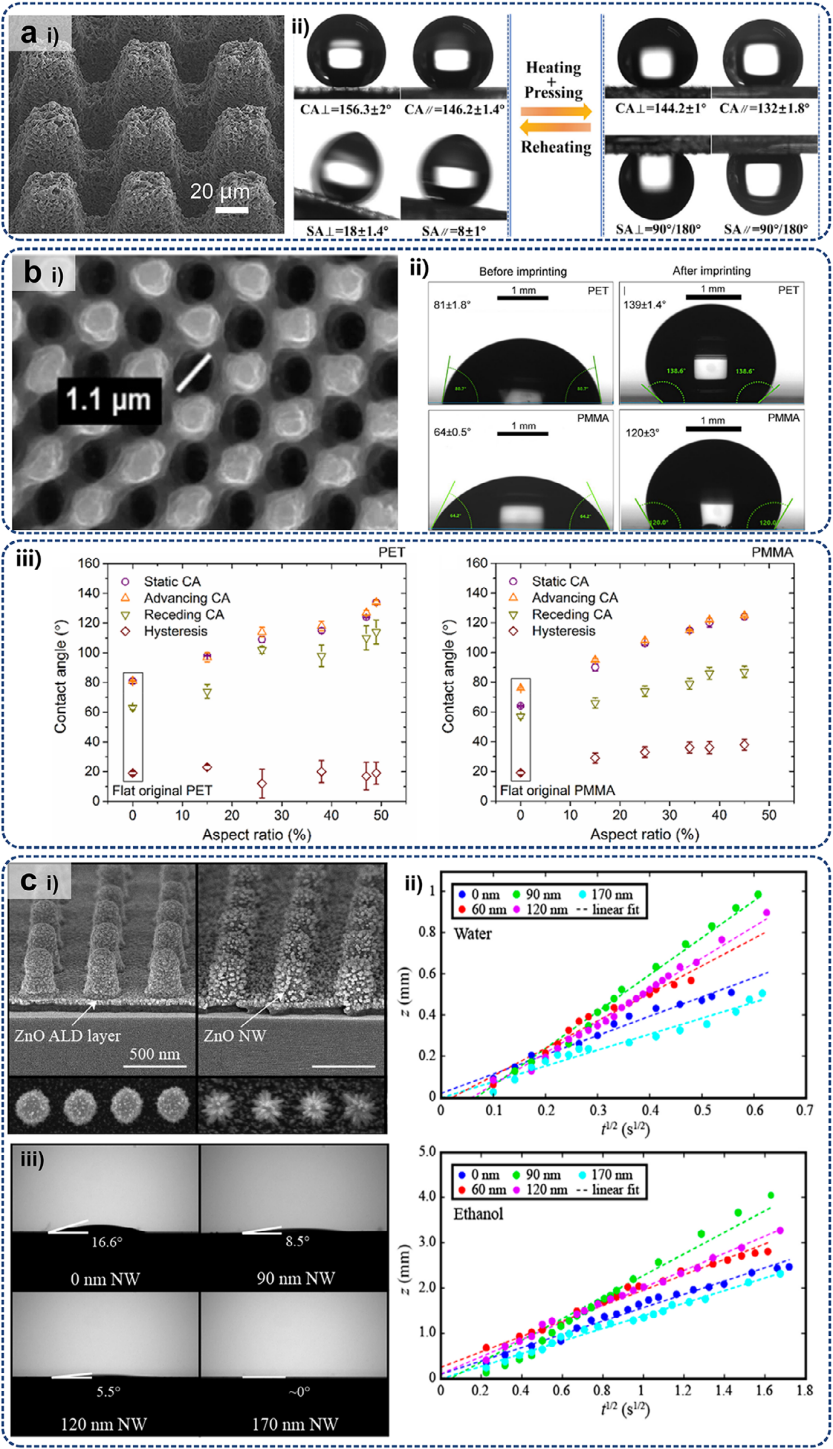
a‐i) Reversible transition of micropillar array microstructures for superhydrophobic properties on laser‐structured PVB substrate. a‐ii) Wettability of the original/recovered SMP microgroove array. Reproduced with permission.^[^ ^289^ ^]^ Copyright 2020, Elsevier. b‐i) Large magnification SEM image of DLIP‐produced hole‐like periodic micropatterns. b‐ii) Water droplets (7 µL) on flat and imprinted PET film. b‐iii) Static contact angles, advancing/receding contact angles, and hysteresis for water on flat and structured PET and PMMA with various texture ratios. Reproduced with permission.^[^ ^291^ ^]^ Copyright 2020, Springer Nature. c‐i) Cross‐sectional and top‐view SEM images of ZnO 3D hierarchical nanostructures with nanowire lengths of 0 and 60 nm. c‐ii) Water and ethanol wicking distance vs. square root of time for 500 nm‐period nanopillar structures with different nanowire lengths. c‐iii) The water contact angle of fabricated nanostructures with varying nanowire lengths. Reproduced with permission.^[^ ^292^ ^]^ Copyright 2016, American Chemical Society.

Existing literature has also focused on surface wettability manipulation of engineered arrays at micro/nanoscales. In earlier research, uniformly distributed microstructures have gained the primary focus of study. Yang et al.^[^ ^281^, ^282^ ^]^ investigated the wetting behavior of porous media composed of siliconized and polymethyl methacrylate (PMMA)‐coated beads and its dependence on capillary properties. Shirazy et al.^[^ ^283^ ^]^ studied the mechanism of the wettability transition from superhydrophilicity to hydrophobicity using copper metal foams. Due to the high capillary forces within micropores, the porous structure exhibits super‐hydrophilic behavior, where the foam immediately absorbs water due to the high capillary action. Li et al.^[^ ^284^ ^]^ experimentally verified the spacing influence of micro‐pillars on wettability. As the results demonstrate, the contact angle increases with the spacing between micro‐pillars, which is attributed to the increase in the proportion of gas‐liquid contact area. Schneider^[^ ^285^ ^]^ investigated the relationship between surface wettability and micro‐cone geometric parameters fabricated by reactive ion etching (RIE). Both the static contact and sliding angle strongly depend on the height and opening angle of micro‐cones. Milles et al.^[^ ^286^ ^]^ specially designed periodic micro‐structured textures on Al substrate for superhydrophobic and ice‐repellent surfaces, featuring scales ranging from 7 to 50 µm, through direct laser writing (DLW) and laser interference patterning (DLIP). Observations reveal that the water contact angle of the untreated reference surface remains almost constant over the studied period, averaging 93°. In contrast, samples treated with either DLW or DLIP methods exhibit an increase in contact angle over time, achieving a temporal transition from hydrophilic to hydrophobic properties. Wang et al.^[^ ^287^ ^]^ proposed Gecko‐inspired polyurethane (PU) micro‐pillar arrays with various tip structures, including spatula, spherical, and concave tips. Luo and Tagawa^[^ ^288^ ^]^ employed the hybrid cascaded lattice Boltzmann and finite difference methods to explore the boiling performance of 3D structured surfaces featuring square micropillars. It is shown that the staggered hydrophilic and hydrophobic array can mitigate bubble retention and prevent heat accumulation. Bai et al.^[^ ^289^ ^]^ explored anisotropic wettability through their laser‐induced shape‐memory polymer (SMP) microgroove arrays (**Figure** **10**a‐i), where the contact angles and sliding angles measured perpendicular to the microgrooves were greater than those measured parallel to the microgrooves, indicating anisotropic superhydrophobicity. Besides, the reversible regulation of slip characteristics was also achieved, as shown in Figure 10a‐ii. Xie et al.^[^ ^290^ ^]^ achieved a reduction in the surface contact angle from 145° to 125° by adjusting the microgroove spacing from 50 to 200 µm. Furthermore, the surface contact angle can be modulated through alterations in the white‐faced structure.

There is growing interest in the heterogeneous wetting characteristics achieved by submicron/nanostructured surface treatments. Fu et al.^[^ ^291^ ^]^ fabricated periodic microstructures on polyethylene terephthalate (PET) and PMMA substrates, with micro‐pillar arrays having periods ranging from 1.6 to 4.6 µm (Figure 10b‐i). These structures were patterned using a chromium stamp, structured by direct laser interference patterning with a four‐beam laser. The introduction of the presented structures achieved a transition from an intrinsically hydrophilic to a hydrophobic surface (Figure 10b‐ii,b‐iii). Ems^[^ ^293^ ^]^ utilized surface microstructures to demonstrate the transition from hydrophilic to hydrophobic wetting states on inherently hydrophilic surfaces composed of re‐entrant cavities. Contact angle measurements indicate that, compared to bare silicon surfaces with a contact angle of 40°, the micro‐fabricated surfaces can maintain a large contact angle exceeding 100°. Yin et al.^[^ ^294^ ^]^ processed controllable patterned and manipulable liquid surfaces on PI films. The laser‐treated samples exhibit a range of surface microstructures and chemical compositions induced by thermal accumulation, enabling continuous and controllable CA from 3.6° to 151.6°. Wang et al.^[^ ^292^ ^]^ investigated developed 3D hierarchical nanostructures with nanocolumn lengths of 0, 90, 120, and 170 nm, as shown in Figure 10c‐i. They found that as the nanocolumn length increased from 0 nm to 170 nm, the wettability was significantly enhanced by a reduction in the contact angle from 16.6° to 0° (Figure 10c‐iii).

## 5 Micro/Nanostructures for Enhanced Phase Transition

### 5.1 Efficient Melting on Contact Interfaces

According to the melting heat dissipation mechanism (Section 2.2.3), three key factors affecting the heat transfer efficiency of phase‐changing material (PCM) include the thermal conductivity of materials, latent heat capacity, and the melting point. Therefore, related researches were dedicated to optimizing the melting heat absorption efficiency from these aspects by employing micro/nanostructures or particles, while ensuring the continuous reliability of the phase transition process.^[^ ^295^, ^296^ ^]^ Common materials used in structured PCM systems include organic materials (such as paraffins, fatty acids, polymers) and inorganic materials (e.g., molten salts or metals, hydrated salts). Among them, paraffins (n‐alkanes, such as octadecane and eicosane) have been popular due to their high latent heats (exceeding 200 Jg^−1^) and chemically benign behavior.^[^ ^297^ ^]^ A typical example is the paraffin‐based PCM, PT58 (melting point of ≈58°C), which is widely investigated and applied combined with graphene or MgO nanoparticle additives and copper foam.^[^ ^298^ ^]^ Additionally, carbon (graphene nanoplatelets, carbon nanotubes, graphite aerogels)^[^ ^299^, ^300^, ^301^ ^]^ or metallic (oxides) nanoparticles (Cu, Al_2_O_3_, Fe_3_O_4_, SiO_2_) are used as primary additives to enhance the thermal conductivity.^[^ ^302^, ^303^, ^304^, ^305^ ^]^ More importantly, microstructural supports such as metal foams,^[^ ^306^, ^307^ ^]^ perforated fins,^[^ ^308^, ^309^ ^]^ and textured surfaces^[^ ^310^ ^]^ have been employed to enhance heat transfer efficiency and uniformity. Furthermore, it should be pointed out that the low‐melting‐point liquid metal or alloy, has been applied as PCM for electronic cooling, which has many favorable properties, such as high thermal conductivity, low vapor pressure and small volume expansion, and large volumetric latent heat of phase transition. However, the drawbacks of high density, toxicity, and high cost limit the practical engineering applications in portable and wearable devices.^[^ ^311^ ^]^ Typical low‐melting‐point liquid metal (LM) or alloy used as PCMs (LMPCMs), including Mercury, Cesium, Gallium, and Rubidium, demonstrate much higher heat transfer capacity (*λ* ranges from 8.1 to 86.9 W·m^−1^·K^−1^) than those common organic or inorganic materials. Details on the thermophysical properties of LMs can be found in ref. [312, 313, 314].

PCMs have been widely used in electronic thermal management systems,^[^ ^15^, ^315^, ^316^, ^317^ ^]^ especially for integrated high‐power electronic devices that require packaging, such as CPUs and servers. Researchers have integrated micro/nano‐composite PCMs into heat sinks and exchangers to buffer generated transient hotspots. Chang et al.^[^ ^318^ ^]^ investigated the thermal performance of a straight finned heat sink filled with paraffin/graphite nanoplatelets (GNPs) composite PCM for thermal management of insulated IGBT. The heat sink with nano‐composite PCM exhibited a temperature reduction of 13.1 °C compared to pure paraffin when operating at a loading capacity of 9 kW. Karthikeyan et al.^[^ ^319^ ^]^ proposed a novel organic PCM with a binary mixture of lauryl alcohol and capric acid, and carbon (GNP)‐metal‐oxide based hybrid nanoparticles, i.e., GNP‐Al_2_O_3_, GNP‐CuO, and GNP‐TiO_2_ were added as thermal conductivity promoters. A notable increment of 60.8% in thermal conductivity is observed for the LA‐CA/GNP‐Al_2_O_3_ composite, which demonstrates high thermal and cyclic stability. Similarly, embedding GNPs and metal‐oxide nanoparticles into the PCM inside a copper‐fin heat sink dramatically lowered device temperatures during bursts. Hassan et al.^[^ ^298^ ^]^ found GNPs‐NePCM/copper foam composites dropped base temperatures by 12.6% at heating input of 2.3 kWm^−2^. Arshad et al.^[^ ^320^ ^]^ developed a nanocomposite PCM using Cu nanoparticles dispersed in RT‐35HC, reducing the melting time duration by 18.1% with the addition of only 1% volume fraction of Cu NPs.

**Figure 11:**
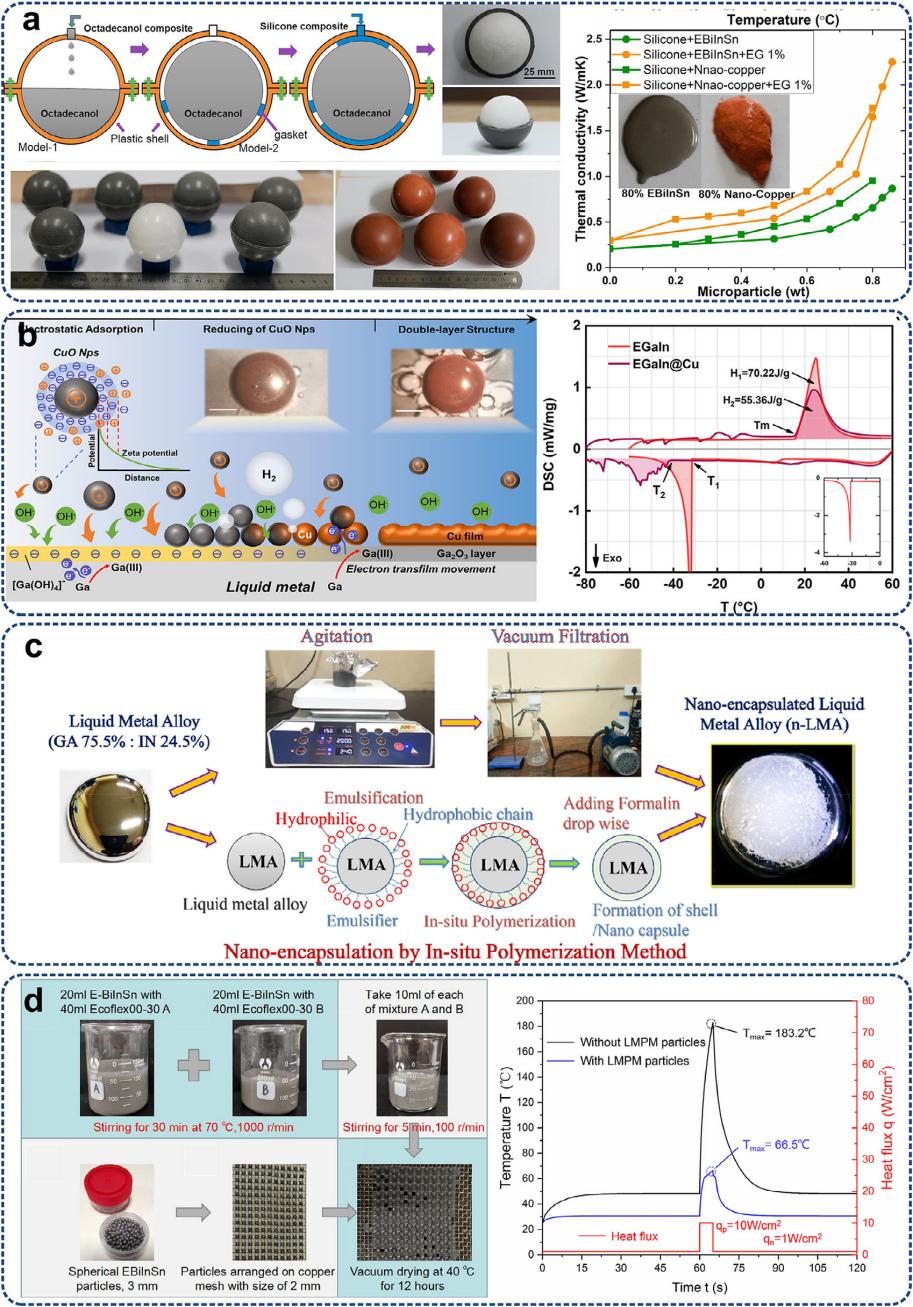
a) Schematic diagram of the preparation process of the spherical PCM macrocapsule, and photos of the macrocapsule core and shell. Reproduced with permission.^[^ ^337^ ^]^ Copyright 2019, Elsevier. b) Schematic diagram of self‐assembly mechanisms with images showing the state transition with a scale bar of 2 mm. DSC curves of EGaIn and EGaIn@Cu. Reproduced with permission.^[^ ^333^ ^]^ Copyright 2021, Elsevier. c) Nano‐encapsulated liquid gallium–indium eutectic metal alloy. Reproduced with permission.^[^ ^334^ ^]^ Copyright 2021, American Chemical Society. d) The preparation of the spherical LMPCM macrocapsules and their effect on the temperature response of the heat sink. Reproduced with permission.^[^ ^338^ ^]^ Copyright 2020, Elsevier.

Furthermore, LMPCMs, especially liquid Ga, demonstrate significant potential in thermal control of compact electronic devices.^[^ ^321^, ^322^, ^323^ ^]^ Previous studies explored LMPCMs for electronics cooling across pure metals, composites, and hybrid systems. Pure Ga‐based LMPCMs consistently outperform organic PCMs in transient heat spreading for phones, LEDs, batteries, and DCs.^[^ ^324^, ^325^ ^]^ For example, heat sinks with Ga reach a high thermal conductivity of around 72 W(m·K)^−1^, which has a reduced volume and weight compared to paraffin composites.^[^ ^326^ ^]^ When applying the Ga‐based LMPCMs for thermal management of lithium‐ion battery packs, battery discharge limits were prolonged by almost five times with improved uniformity.^[^ ^327^ ^]^ However, the drawbacks of Ga‐based LMPCMs (e.g., high density and cost, corrosion to copper and aluminum, and leakage/short‐circuit risks) motivate the development of composite and system‐level designs.^[^ ^311^ ^]^ A paste‐like LMPCM with high thermal conductivity (≈21 W·m^−1^·K^−1^), small supercooling degree (≈8.8 K), and large volumetric latent heat (≈308 Jcm^−3^) was prepared by incorporating high‐loading miscible Ga_2_O_3_ particles within Ga liquid metals, which can be used for high‐performance temporary heat dissipation of intermittent‐use electronic devices.^[^ ^66^ ^]^ High weight and cost were mitigated by mixing LMs with paraffin in expanded graphite to prevent phase separation and to tune latent heat (211–70 Jg^−1^) and conductivity (2.6–39.2 W m^−1^ K^−1^).^[^ ^328^ ^]^ There is an optimum proportion of Ga enabling the longest thermal control time in dual‐PCM cascades.^[^ ^329^, ^330^ ^]^ Besides, heat conduction can be further boosted via built‐in fins^[^ ^331^ ^]^ and carbon/metal porous matrices. Micro/nano‐encapsulation of LMPCMs has been an effective strategy to curb leakage^[^ ^332^, ^333^, ^334^, ^335^, ^336^ ^]^ (**Figure** **11**). The phase change LM microcapsules using PMMA as the shell achieved a lower LED‐chip (at 71.1 °C) temperature by 11.2 °C than that of the single thermal gel case.^[^ ^336^ ^]^ More importantly, hybrid thermal management strategies coupling LMPCMs to other cooling technologies (heat pipes, liquid cooling, and pumped loop systems) are promising to overcome duration limits under sustained thermal loads, broadening applicability to high‐power electronics. The progress and prospects of related works have also been reviewed recently.^[^ ^311^ ^]^

### 5.2 Nucleation Site Generation

**Figure 12:**
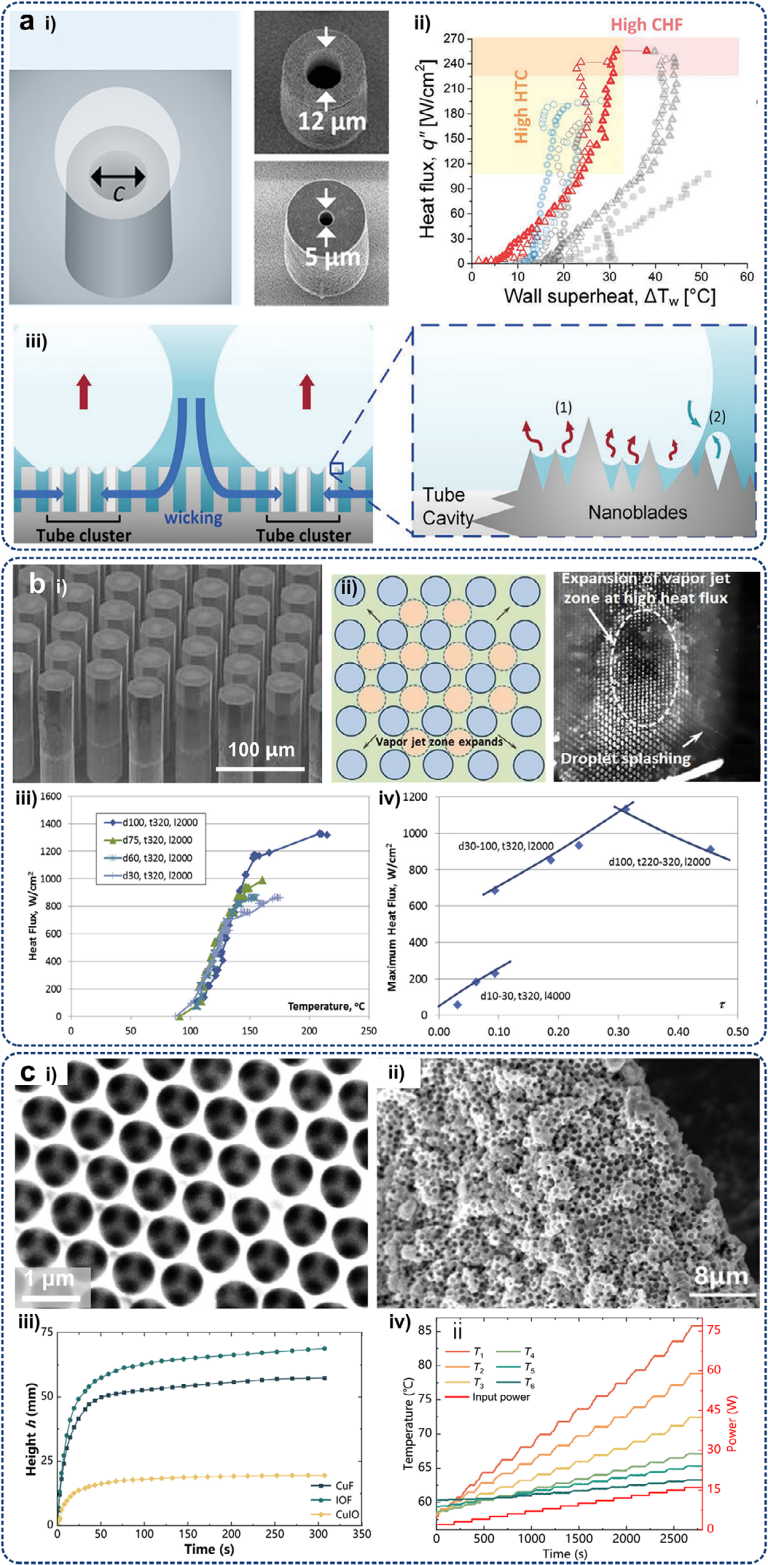
a‐i) Schematics and SEM images of the TIP surface, exhibiting capillary wicking while controlling vapor nucleation. a‐ii) Pool boiling (water) performance of h‐Tube and h‐TIP surfaces compared with surfaces without nanostructures. a‐iii) Schematic of the separated liquid–vapor paths during boiling on an h‐TIP surface. Reproduced with permission.^[^ ^347^ ^]^ Copyright 2022, Wiley‐VCH. b‐i) SEM image of silicon wick structures with a height of 320 µm and a diameter of 100 µm. b‐ii) Schematic and optical images of the active vapor jet zone at a high heat flux of 1130 Wcm^−2^. b‐iii) Heat flux versus the substrate temperature of the wick sample with different wick thicknesses. b‐iv) The maximum heat fluxes vary with the vapor phase geometrical numbers. Reproduced with permission.^[^ ^348^ ^]^ Copyright 2014, Elsevier. c‐i) Large magnification SEM image of CuIO structural sample. c‐ii) Microscopic visualization of CuIO modified on CuF (IOF). c‐iii) Time‐dependent capillary suction heights of three samples. c‐iv) Temperature variation of a UHP containing IOF used as porous wick (porosity of 54%). Reproduced with permission.^[^ ^352^ ^]^ Copyright 2024, Elsevier.

To achieve an optimal boiling heat transfer performance, it is believed that the following characteristics are desirable: moderate bubble size, uniform nucleation sites, high bubble generation frequency, rapid bubble detachment, regular bubble shape, and stable bubble‐induced disturbance,^[^ ^339^, ^340^, ^341^, ^342^ ^]^ which are closely related to the nucleation site arrangement. In the current mainstream research on the electronic cooling system, the micro/nano pin, pillar, and wire have been recognized as useful designs to improve heat transfer and bubble nucleation. Liao et al.^[^ ^343^ ^]^ developed a two‐phase microchannel cooling system covered with nucleated columnar pin‐fins, which periodically enabled bubble generation at the edge of the nucleated fin pore. Deng et al.^[^ ^344^ ^]^ arranged the micro cone pin fins at the bottom of microchannels (SM‐MPF), providing many stable nucleation sites with tiny reentrant cavities and introducing significant wicking effects to maintain the liquid rewetting. Khan et al.^[^ ^345^, ^346^ ^]^ performed a nucleate boiling performance assessment of a special‐design porous coating with microparticles, nanoparticles, and their mixture. The results showed that the heat transfer coefficient (HTC) and critical heat flux (CHF) were significantly enhanced due to micro/nano‐coating, which provides improved specific heating area, capillary effect, and bubble departure rate. However, the micro‐nano hybrid surface demonstrated a deterioration of the boiling performance, resulting from the damage to the capillary wicking. This damage disrupts the delicate balance between the liquid supply and vapor escape. By incorporating microcavities within hemi‐wicking structures, Song et al.^[^ ^347^ ^]^ overcame the intrinsic trade‐off between HTC and CHF by controlling vapor nucleation at multiple length scales on a hierarchically tube‐clusters in pillars (h‐TIP) surface (**Figure** **12**a‐i). Pool boiling tests showed enhancements of HTC and CHF on presented structures (Figure 12a‐ii), which were further enhanced by including nano‐blades atop the microstructures (Figure 12a‐iii). In addition, researchers have constructed arrayed structures, including conical, pyramidal, spherical (Figure 12b‐i), and rectangular prism,^[^ ^348^, ^349^, ^350^ ^]^ aiming to mitigate flow resistance and improve the surface liquid supply, as shown in Figure 12b‐ii. An ultra‐high heat flux (1130 Wcm^−2^) was achieved by specially designed geometrical parameters (Figure 12b‐iii,b‐iv). Recently, Fan et al.^[^ ^340^, ^351^ ^]^ presented an annular inverse opal (AIO) surface with a gap for liquid replenishment by attenuating the coffee ring effect (Figure 12c‐i). The bubble growth rate, CHF (214%), and HTC (240%) were significantly increased (75%), according to the phenomenological observation, resulting from the lateral movement of the liquid when nanopores existed. Furthermore, the copper inverse opal (CuIO) modified sintered copper foam (CuF), IOF, was applied as the capillary wick in an ultrathin heat pipe (UHP) with a thickness of less than 1 mm, which allowed a GaN chip to work at a heat flux of 208 Wcm^−2^.^[^ ^352^ ^]^ It is displayed in Figure 12c‐ii that a regular arrangement of nanopores on the surface of metal foam was fabricated, which plays a crucial role in liquid replenishment (Figure 12c‐iii) and facilitating bubble detachment (Figure 12c‐iv). Optimizing bubble nucleation, growth, and departure through micro/nanostructures is critical for advancing two‐phase flow cooling strategies. Compared to single‐phase cases, boiling microfluidics integration with directional thermal conduction promises to enable targeted thermal management. However, current research on micro/nanostructures for bubble morphology control remains insufficient. The regulation of bubble aggregation and departure dynamics demands profound inspiration from biomimetic or engineered solutions.

### 5.3 Liquid Replenishment (Wickability)

**Figure 13:**
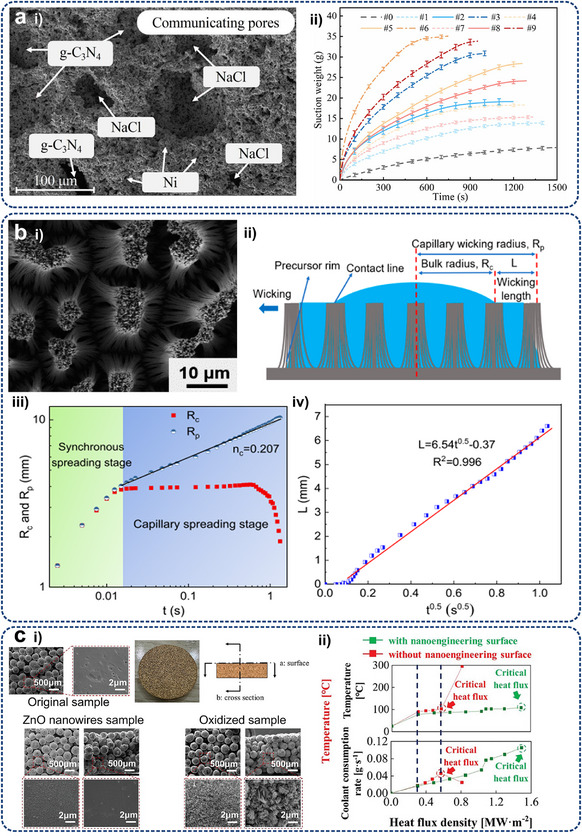
a‐i) SEM image of the multiscale porous wick fabricated using NaCl and g‐C_3_N_4._ a‐ii) The suction weight curves of porous wicks. Reproduced with permission.^[^ ^354^ ^]^ Copyright 2024, Elsevier. b‐i) SEM image of the NW‐A3 hierarchical nanowired surfaces as an example. The height and diameter are 9‐14 µm and 110 nm. b‐ii) A Schematic of capillary process in nano‐wired structures with parameter analysis. b‐iii) Variation of the contact line and the precursor rim radius with time on NW‐A3. b‐iv) The relationship between wicking lengths and the square root of time *t* ^0.5^ on NW‐A3. Reproduced with permission.^[^ ^362^ ^]^ Copyright 2021, American Chemical Society. c‐i) SEM characterization of sintered bronze samples (*d*_p_=300 µm), the ZnO nanowires sample, and oxidized samples. c‐ii) Comparison of the coolant consumption rate and temperature of samples with or without nanoengineered surfaces with increasing heat flux densities. Reproduced with permission.^[^ ^364^ ^]^ Copyright 2024, Wiley‐VCH.

Capillary‐driven liquid replenishment via micro/nanostructures is a key mechanism against dry‐out during boiling heat transfer.^[^ ^76^ ^]^ When bubbles cover the heated surface, the working fluid is delivered continuously within a porous framework to nucleation sites through lateral transport and vertical suction, resulting from spontaneous capillary forces,^[^ ^353^, ^354^, ^355^ ^]^ which enables rewetting and sustains efficient heat dissipation. Kumar et al.^[^ ^356^ ^]^ proposed a CuO nanowires porous layer on copper substrates to improve CHF by 200%, which boosts capillary suction pressure, accelerating bubble departure frequency, and delaying dry out. Kim et al.^[^ ^357^ ^]^ explained the boiling mechanism at micro/nanoscales by the wicking and pumping action of capillary as transpiration. The liquid was induced to enter the area below the bubble, promoting the bubbles’ departure from the surface. A similar phenomenon was observed and investigated on the surface of silicon nanotube arrays used for nucleate boiling heat dissipation.^[^ ^358^ ^]^ Kim et al.^[^ ^359^ ^]^ systematically explored the underlying theory between wicking dynamics and CHF using arranged nanopillars with varying characteristic lengths. It is found that a high wicking coefficient leads to a 164% improvement in CHF, which was previously underestimated by classic models related to wettability and surface roughness. Liu et al.^[^ ^354^ ^]^ developed a hydrophilic wick with porous microstructures used for an evaporator for a two‐phase loop system (**Figure** **13**a). It achieved a high heat load of 400 W due to its desired liquid replenishment performance, characterized by highly connected and multi‐morphology pores. Advanced cooling technologies have become critical for thermal protection under extreme conditions involving high heat fluxes and temperatures, as demonstrated by recent research on capillary‐driven transpiration cooling. Kim et al.^[^ ^359^ ^]^ focused on the dynamics of capillary wicking and its impact on the CHF for enhanced boiling heat transfer. Hexagonally arranged submicron‐scale nanopillars were utilized for precise wicking control of wicking. The reduction in the spacing between the nanopillars enhanced the wicking distance, which led to an increase in the CHF. By using surface modification of aluminum fins, Lou et al.^[^ ^360^, ^361^ ^]^ introduced a new type of nano‐capillary evaporative finned heat sinks, which experimentally validated to be 7.8–8.4 times higher passive cooling power than conventional designs.^[^ ^361^ ^]^ Hybrid approaches, such as combining sublimation with transpiration cooling, have addressed localized hot spots (e.g., stagnation regions) by activating transpiration locally via sublimable Teflon coatings, thereby increasing cooling effectiveness by up to 63%.^[^ ^361^ ^]^ Ma et al.^[^ ^362^ ^]^ introduced hierarchical copper nanowired surfaces with interconnected V‐grooves as an effective solution for enhancing capillary wick, shown in Figure 13b‐i. The authors demonstrated that the combined effects of copper nanowires and V‐grooves significantly improve the wicking effect, schematically illustrated in Figure 13b‐ii. The nanowires provided high capillary pressure, while the V‐grooves acted as transport channels to reduce viscous resistance (Figure 13b‐iii,b‐iv). Additionally, Jiang and his coworkers^[^ ^363^ ^]^ combined transpiration and film cooling in sintered metal porous struts, which proved effective, with non‐uniform coolant injection improving front‐part cooling efficiency from 25.7% to 37.9% by targeting high‐heat‐flux zones. Recently, they originally developed a nanostructure‐engineered capillary system^[^ ^364^ ^]^(Figure 13c), leveraging evaporation‐driven flow, to perform a CHF up to 3.1 MWm^−2^, where Al_2_O_3_ and titanium alloy (Ti‐6Al‐4V) micro‐scale particles and surface nanostructures were employed to improve their wettability and capillary action.

In summary, to maximize the heat transfer efficacy associated with phase transition, research efforts have focused on accelerating melting rates, improving melting uniformity, optimizing bubble dynamics, enhancing liquid replenishment, balancing liquid‐vapor transport, and integrating with complementary cooling technologies. Nevertheless, the spatial constraint of integrated devices poses challenges to the direct applicability of classic theories and practical micro/nanostructures related to phase transition heat transfer, while simultaneously inspiring a range of compelling scientific and engineering directions. Future advancements in phase‐changing thermal management for electronics will depend on a fundamental shift toward multiscale structural coordination, adaptive gas‐liquid management, in‐depth integration, and dynamic‐response heat transfer modeling. In particular, the synergistic effect of micro‐/nanoscale structures should be harnessed to simultaneously regulate melting propagation, bubble dynamics, and liquid transport behavior. Materials or engineering structures with dynamically tunable or smart‐responsive surface wettability are required to enable adaptive reconstruction of liquid transport paths in local high–heat‐flux areas. Practical deployment that couples phase‐change cooling units with broader cooling systems for optimal integration is highly recommended. Last but not least, structure–performance inverse optimization strategies combined with machine learning should be considered to accelerate the closed‐loop process of thermal management deployment. Cooling performance of typical micro/nanostructures used for enhanced convection and phase‐changing (evaporation) can be found in Table S1 (Supporting Information).

## 6 Micro/Nanostructures for Enhanced Radiation

### 6.1 Radiation Control by Micro/Nanostructures

By tailoring the geometry, periodicity, and material combinations of micro/nanostructures, precise control over the wavelength, intensity, directionality, and polarization of Infrared (IR) radiation has untapped potential for advancing efficient thermal management, optoelectronic devices, IR stealth technologies, and energy applications.^[^ ^91^, ^93^, ^365^ ^]^ Micro/nanostructured texture modifies the emissivity and absorptivity of material surfaces, influencing IR radiation characteristics. The directionality, angular distribution, and radiative intensity can be controlled by tuning nano‐scaled roughness to enhance multiple scattering events. Furthermore, micro/nanostructured devices, such as photonic crystals or nano‐gratings, introduce periodic variations in dielectric constants or refractive indices, forming photonic bandgaps, which selectively suppress or enhance emission propagation.^[^ ^366^ ^]^ Introducing nonreciprocal metamaterials (e.g., MO materials or topological insulators) into micro/nanostructures breaks the time‐reversal symmetry of thermal radiation (Kirchhoff's law), paving the way for thermal diodes and non‐reciprocal emitters. Therefore, crucial factors influencing radiation heat transfer are the temperature of subjects, radiative surface area, and emissivity at the specific wavelength and direction, whose performance can be characterized by cooling power and temperature response rate.^[^ ^93^ ^]^

### 6.2 Radiative Cooling

Terrestrial passive radiative cooling (PRC) dissipates heat to the universe by adjusting the balance of radiative energy flow.^[^ ^91^, ^94^ ^]^ Cooling sky‐facing objects to a temperature below the environment can be achieved by minimizing their heat gain while maximizing the outgoing thermal radiation via the atmospheric window, i.e., 8 to 13 µm, at which the highly efficient radiative energy exchange with low losses is realizable between the Earth and outer space. Compared with other conventional thermal management technologies, the distinctive strength of radiative cooling is that it directly sends waste heat to outer space at an extremely high rate instead of depositing it in the surrounding environment. With the development and technological maturity of nanofabrication processes, several types of radiative coolers, including multilayer thin film,^[^ ^367^, ^368^, ^369^, ^370^ ^]^ metamaterials,^[^ ^371^, ^372^, ^373^, ^374^, ^375^, ^376^ ^]^ and polymer,^[^ ^377^, ^378^, ^379^, ^380^, ^381^ ^]^ have been widely investigated in the past decades, which were specially designed based on bio‐inspired or engineered nanostructures.^[^ ^382^, ^383^ ^]^

**Figure 14:**
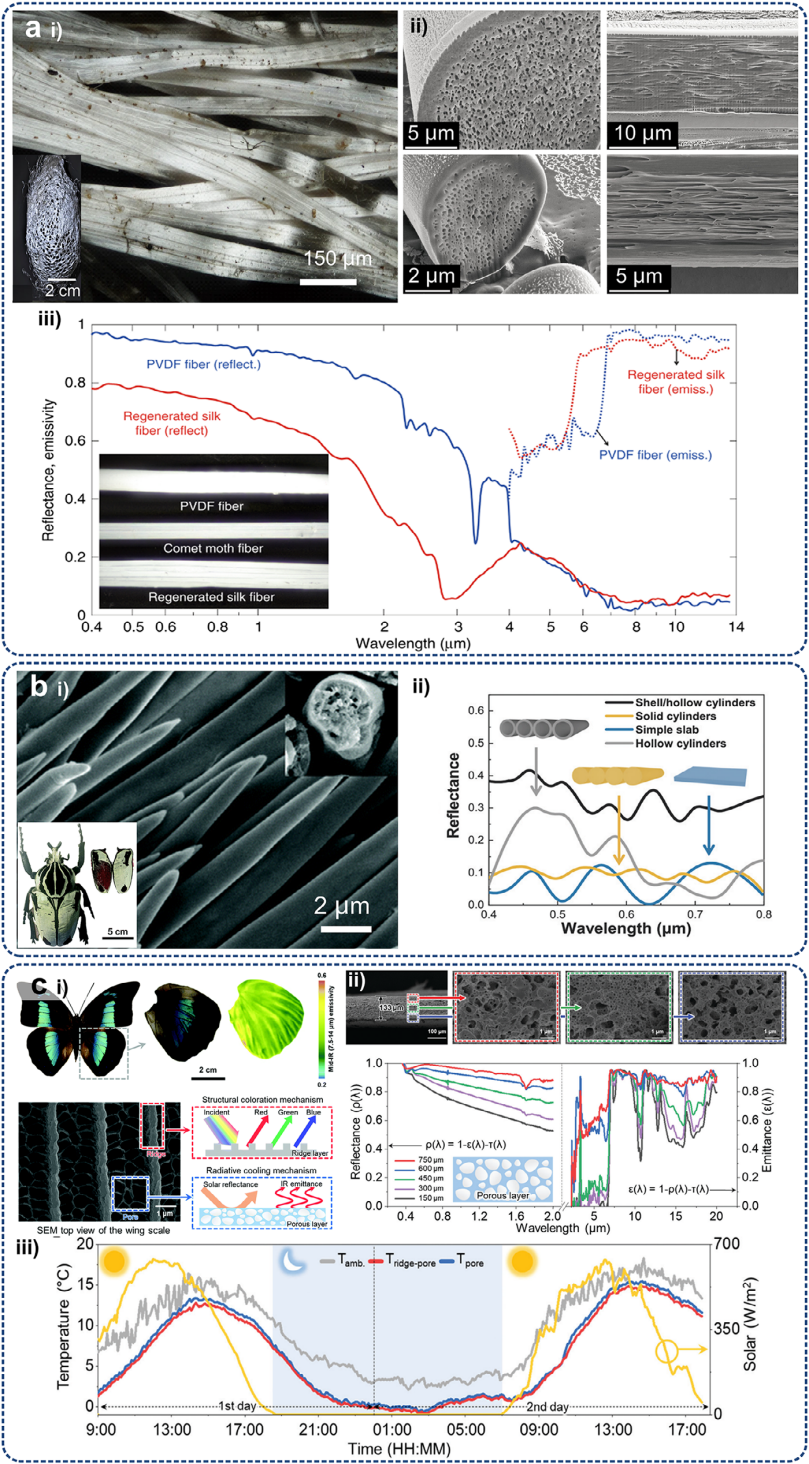
a‐i) Dark‐field optical microscopy image showing overlapping cocoon fibers. Inset: Photo of comet moth cocoon with a reflective sheen. a‐ii) SEM images of transverse and longitudinal cross‐sections of regenerated silk and PVDF fibers. a‐iii) Optical properties of a bundle of regenerated silk fibers and a single PVDF fiber from the visible to the mid‐infrared region. Reproduced with permission.^[^ ^384^ ^]^ Copyright 2018, The Royal Society of Chemistry. b‐i) SEM microscopy image of the central part of white scales. Inset: Cross‐sectional image and photograph of a male Goliathus goliatus. b‐ii) Reflectance comparison of bio‐inspired structure to solid cylinders, simple slab, and hollow cylinders. Reproduced with permission.^[^ ^385^ ^]^ Copyright 2019, Springer Nature. c‐i) Photograph of the entire body of Archaeoprepona demophon and its dorsal wing with its corresponding mid‐IR emissivity mapping, microstructures of the dorsal wing, physical mechanisms of induced structural coloration, and PRC based on ridge nanograting and porous structures, respectively. c‐ii) Microscopic images of a porous structure cross‐section, including the pores of different sizes across the layer uniformly and their optical properties changing with film thickness. c‐iii) Radiative cooling performance of the porous film and integrated biomimetic film. Reproduced with permission.^[^ ^386^ ^]^ Copyright 2022, The Royal Society of Chemistry.

Nature has evolved various biological systems with fascinating optical properties that can inspire the development of PRC technologies. In 2018, Shi et al.^[^ ^384^ ^]^ investigated silk cocoon fibers of the Argema mittrei (**Figure** **14**a‐i). They found that they are populated with a high density of air voids randomly distributed across the fiber cross‐section (Figure 14a‐ii), which strongly scatters light in the solar spectrum, resulting in a high solar reflectance. Additionally, the chemical bonds of the silk proteins lead to a high emissivity in the mid‐infrared (Figure 14a‐iii). In 2019, inspired by the white beetle Goliaths goliaths, Xie et al.^[^ ^385^ ^]^ investigated the morphology and optical properties of wing scales (Figure 14b‐i), revealing that the porous structure enhances visible reflectance and infrared emittance (Figure 14b‐ii). They mimicked this nanostructure to develop a biomimetic film using a SiO_2_ sacrificial template‐based solution process and laser‐interference lithography. The film demonstrated a maximum temperature reduction of 8.45 °C and a color gamut covering 91.8% of the standardized color profile (sRGB). More recently, Lee et al.^[^ ^386^ ^]^ developed a multifunctional biofilm that mimics the nanostructure of the Archaeoprepona demophon wing scales, which exhibited radiative cooling and structural coloring functionalities, as depicted in Figure 14c‐i. They used a solution‐based process to create a porous structure and laser interference lithography to generate the ridge structure. Figure 14c‐ii shows that multiple‐scaled nanopores were produced in a porous (polyvinylidene fluoride cohexafluor‐opropylene) PVDF‐HFP layer, whose optical properties strongly depended on the thickness. They demonstrated a maximum temperature reduction of 8.45 °C for the integrated film during all‐day testing (Figure 14c‐iii), and the ability to generate different colors based on the imprinted ridge structure. Cheng et al.^[^ ^387^ ^]^ proposed a PRC film inspired by natural wrinkle structures of human skin, combined with optimized BaSO_4_ and SiO_2_ particles to achieve efficient optical property regulation for both solar and atmospheric window bands. The coating with a thickness of approximately 100 µm reflected 95% of solar irradiance, and an emissivity of 96% was observed in the atmospheric window band.

**Figure 15:**
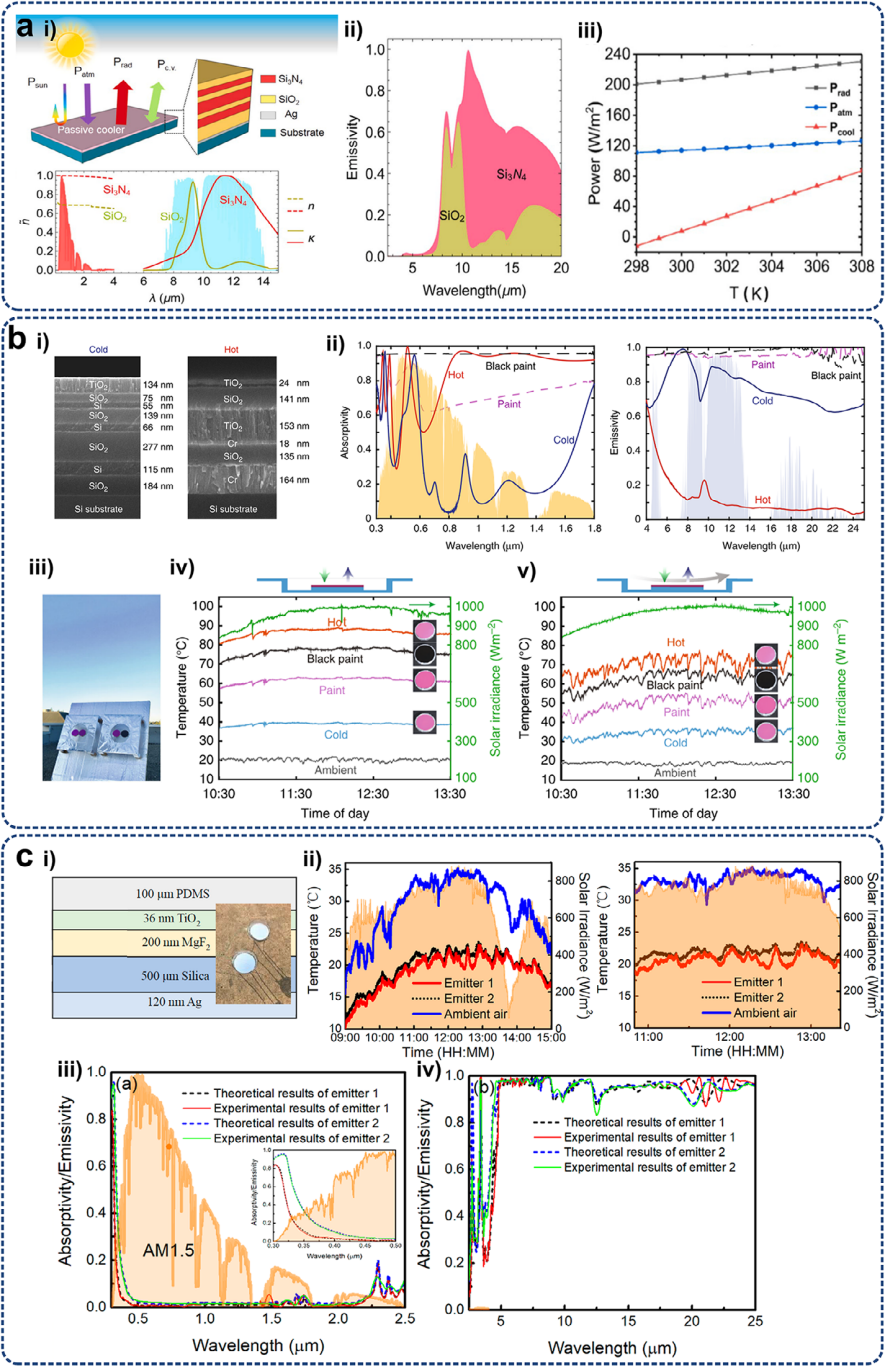
a‐i) Schematic of the proposed radiative cooler with random layers designed based on the genetic algorithm and normalized complex refractive indexes of SiO_2_ (green lines) and Si_3_N_4_ (red lines). a‐ii) Spectral contribution of SiO_2_ (green region) and Si_3_N_4_ (pink region) layers to the total emissivity over the mid‐infrared band. a‐iii) Temperature‐dependent powers of the presented radiation cooler. Reproduced with permission.^[^ ^367^ ^]^ Copyright 2020, Springer Nature. b‐i) Cross‐section SEM images of the cold and hot thermos‐photonic structures. b‐ii) Absorptivity and Emissivity spectrum of the cold (blue curve) and hot (red curve) emitters, the pink paint (dashed purple curve), and the black paint (dashed black curve) samples, where the yellow and blue shaded areas represent normalized AM 1.5 solar spectrum and atmosphere transmittance, respectively. b‐iii) Photo of the testing apparatus on the rooftop. Temperature measurements of four samples, ambient temperature (light grey), and solar irradiance (green) at noontime b‐iv) without or b‐v) with convection effects. Reproduced with permission.^[^ ^370^ ^]^ Copyright 2018, Springer Nature. c‐i) Schematic of multilayer PRC film including five materials. c‐ii) Measured and calculated emissivity/absorptivity of emitter 1 (red solid curve) and emitter 2 (green solid curve) at 12° angle with the normalized AM1.5 solar spectrum. (Inset) Results for two emitters in the UV region. c‐iii) and c‐iv) Optical properties of emitter 1 (red solid curve) and emitter 2 (green solid curve) at a 30° angle of spectra. Reproduced with permission.^[^ ^388^ ^]^ Copyright 2019, MDPI (Basel, Switzerland).

Multilayer hybrid PRC film can achieve a specific electromagnetic spectrum in a predesigned wavelength range resulting from thin‐film interference, which is composed of a thin metal film (e.g., Ag and Al) featuring high reflection in solar spectra to reduce energy absorption and the stack of absorbing/dielectric layers (e.g., SiO_2_, TiO_2_, and Si_3_N_4_) for high emissivity in IR regime.^[^ ^93^ ^]^ Ma et al.^[^ ^367^ ^]^ designed a radiative cooler consisting of seven layers of SiO_2_ and Si_3_N_4_, which enabled a high solar irradiation reflection and broadband emissivity by complementary phonon resonances (**Figure** **15**a‐i,a‐ii). The cooling power calculation indicated a strong inverse dependence on the temperature, which required external energy to achieve a balanced state (Figure 15a‐iii). Li et al.^[^ ^370^ ^]^ theoretically analyzed the radiation thermal power of samples depicting different colors via multilayer thermal‐photonic structures (Figure 15b‐i). Two devices exhibiting different surface thermal performance with the same color were defined as ‘cold’ and ‘hot’ emitters, characterized by their absorptivity/emissivity in visible, solar, and mid‐infrared spectra, as shown (Figure 15b‐ii). Outdoor testing showed a high average temperature difference (35.4 °C) between the two emitters for a measurement period (Figure 15b‐iii,b‐iv,b‐v). Zhu et al.^[^ ^388^ ^]^ fabricated a multilayer radiative cooler with high solar reflectance for daytime PRC applications (Figure 15c‐i). Figure 15c‐ii reveals an average temperature reduction of around 12.6 °C for one of the emitters, even with a significant solar irradiance. The emissivity of the proposed film was shown to be angle‐independent, which was used to maximize the radiative power in practice (Figure 15c‐iii,c‐iv).

**Figure 16:**
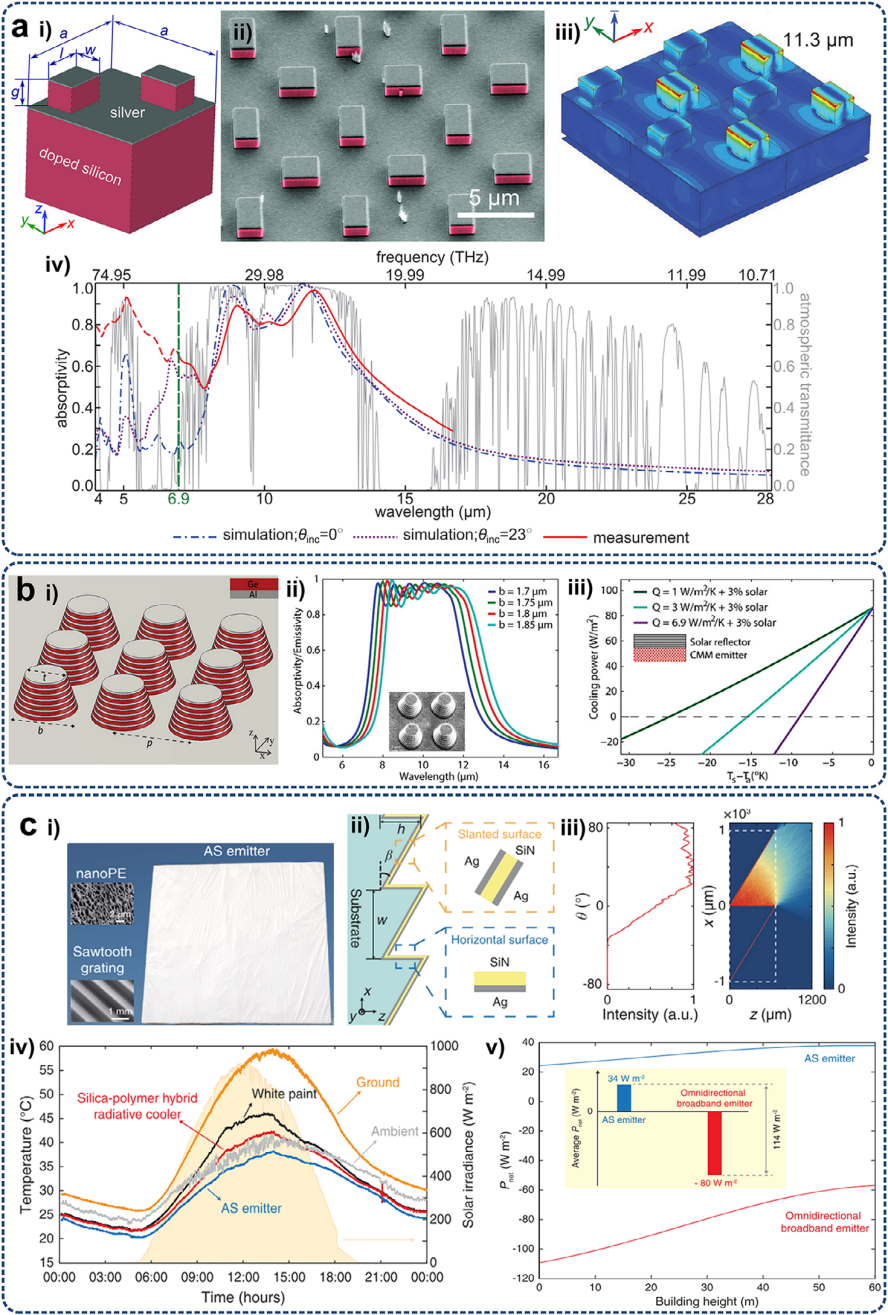
a‐i) Schematic of proposed metamaterials for radiation cooling. a‐ii) SEM images of subwavelength dielectric resonator metasurface with 100 nm thick silver layer coating. a‐iii) Simulated *E*‐field distribution on the metasurface @11.3 µm. a‐iv) Comparison of simulated and measured absorptivity. The blue dotted‐dashed and purple discrete lines represent results under normal incident excitation and 23° incidence, and the red solid line is the measured absorptivity at the angle ranging from 15° to 30°. The gray background is atmospheric transmission as a benchmark. Reproduced with permission.^[^ ^382^ ^]^ Copyright 2015, Wiley‐VCH. b‐i) Schematics of CMM pattern. b‐ii) Simulated and c) Measured emissivity/absorptivity of metasurface changing with the bottom width of CMM pillars, and magnified SEM images of the CMM structures (bottom width=1.75 µm). b‐iii) Calculated cooling power of the CMM metamaterial with 3% solar absorption and radiative insulation. Reproduced with permission.^[^ ^374^ ^]^ Copyright 2017, Wiley‐VCH. c‐i) Photograph of the 35 × 35 cm^2^ AS radiation cooler. c‐ii) Schematic of the AS emitter consisting of a sawtooth grating covered by a UV–VIS–reflective, IR‐transparent nanoPE film. c‐iii) Calculated angular (left) and spatial (right) distribution of thermal radiation intensity at the wavelength of 11 µm. c‐iv) Cooling performance of AS emitter under full‐day solar irradiance (a peak solar irradiance of >920 W/m^2^). c‐v) Comparison of net cooling power at different locations on a building wall covered with an AS emitter and an omnidirectional broadband emitter. Reproduced with permission.^[^ ^373^ ^]^ Copyright 2024, AAAS.

Plasmonic nanostructures and metamaterials have been numerically investigated and experimentally validated as radiative coolers with desired performance. In 2012, Guha et al.^[^ ^389^ ^]^ experimentally demonstrated near‐field radiative cooling of thermally isolated nanostructures with a local temperature reduction of up to 1.5 K. Based on this pioneering observation, Zhu et al.^[^ ^390^ ^]^ demonstrated a thermophotonic approach using a silica photonic crystal as a visibly transparent thermal blackbody to cool solar absorbers. Placing the silica photonic crystal on top of a silicon substrate lowered the temperature of the substrate by up to 13 °C while maintaining and even slightly enhancing solar absorption. Zou et al.^[^ ^382^ ^]^ developed a type of metasurface with nano‐structured square prisms and silver coating to tailor its emissivity and absorptivity for radiative cooling performance (104.5 Wm^−2^) (**Figure** **16**a‐i,a‐ii). As shown in Figure 16a‐iii, the simulated *E*‐field distributions illustrated the high resonances along the length of the structures at 8.8 µm. A good agreement between experimental and simulated absorptivity (or emissivity) was obtained to validate the modeling (Figure 16a‐iv). Hossain et al.^[^ ^374^ ^]^ designed a strictly selective yet broadband metamaterial emitter based on an array of symmetrically shaped conical metamaterial (CMM) pillars (Figure 16b), which achieved a cooling power of 116.6 Wm^−^^2^ at the ambient temperature. A significant enhancement of emissivity/absorptivity was shown over the mid‐infrared spectrum based on calculation, which was further experimentally demonstrated. Recently, Xie et al.^[^ ^373^ ^]^ originally developed a sub‐ambient daytime angularly asymmetric, spectrally selective (AS) radiation cooling on vertical surfaces rather than facing the sky, which exhibited a higher cooling power (≈114 Wm^−2^) than omnidirectional broadband film due to the angularly asymmetric feature. It consists of a sawtooth grating covered by an ultraviolet‐visible (UV‐VIS) reflective IR transparent nanoporous polyethylene (nanoPE) film (Figure 16c‐i). The silicon nitride (SiN) layer with a thickness of 4 µm on the grating (Figure 16c‐ii) enables spectrally selective thermal emission at specific angles, as shown in Figure 16c‐iii.

**Figure 17:**
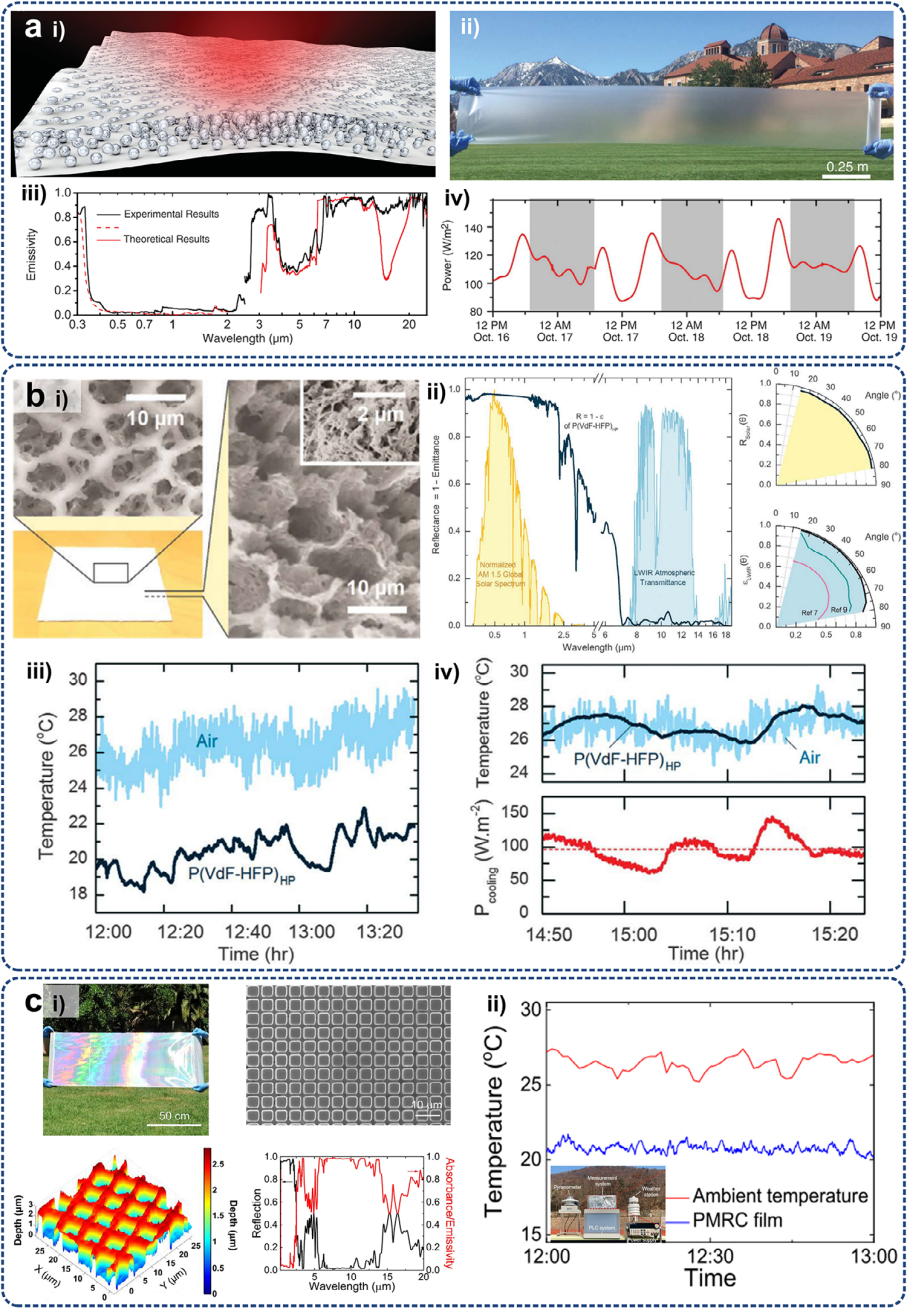
a‐i) Schematical diagram of the glass‐polymer hybrid film with randomly distributed SiO_2_ microsphere inclusions. a‐ii) photo showing the 300‐mm‐wide hybrid metamaterial thin film produced by a roll‐to‐roll method. a‐iii) Comparison of experimental and theoretical optical properties (emissivity/absorptivity) of the presented hybrid metamaterial. a‐iv) Measured radiative cooling power over 3 days shows an average cooling power of >110 Wm^−2^ and a noontime cooling power of 93 Wm^−2^. Reproduced with permission.^[^ ^383^ ^]^ Copyright 2017, AAAS. b‐i) Microscopic showing top and cross‐section views of P(VdF‐HFP)_HP_ with the nano‐porous features. b‐ii) Spectral and angular reflectance of a 300‐mm‐thick film coating presented against normalized Global solar spectrum and the atmospheric window. b‐iii) Temperature reduction of the given film at noontime. b‐iv) Temperature reduction and average cooling power over time in the afternoon. Reproduced with permission.^[^ ^391^ ^]^ Copyright 2018, AAAS. c‐i) Photograph of the fabricated PRC film, top‑view SEM image, optical profilometer 3D image, and measured optical parameters. c‐ii) Real‑time temperature drop of proposed PMRC film. Reproduced with permission.^[^ ^376^ ^]^ Copyright 2024, Springer Nature.

Polymer offers great potential in PRC film preparation, including designable thermos‐optical properties, chemical stability and durability, and ease of large‐scale manufacturing at a low cost.^[^ ^383^ ^]^ In 2017, Zhai et al.^[^ ^383^ ^]^ developed a glass‐polymer hybrid metamaterial containing SiO_2_ microparticles for radiative cooling, as schematically shown in **Figure** **17**a‐i,a‐ii. The spectroscopic performance characterized by Fourier transform infrared (FTIR) spectrometer and spectrophotometer showed an ultra‐high solar reflection (≈96%) and emissivity (>93%) (Figure 17a‐iii). Consequently, an average cooling power of around 93 Wm^−2^ was observed (Figure 17a‐iv). Mandal et al.^[^ ^391^ ^]^ focused on a daytime PRC film with hierarchically porous poly (vinylidene fluoride‐co‐hexafluoropropene) [P(VdF‐HFP)_HP_] coatings, as depicted in Figure 17b‐i. The selective spectroscopic properties (Figure 17b‐ii) lead to an average temperature drop of around 6°C (Figure 17b‐iii) and an exceptional cooling power of 96 Wm^−2^ achieved in Phoenix, USA (Figure 17b‐iv). Recently, Lin et al.^[^ ^376^ ^]^ presented a novel approach to PRC using a polymer metasurface with periodically arranged 3D trench‐like structures in a thin polymer layer creatively manufactured by a highly efficient roll‐to‐roll printing method. The film exhibited low solar absorptivity (4.8%) and high average emissivity (96.1%) in the atmospheric window (Figure 17c‐i). A temperature reduction of 7°C was observed by outdoor testing, as shown in Figure 17c‐ii. The impressive cooling powers of 129.8 and 104.2 Wm^−^^2^ have been achieved at daytime and nighttime, respectively.

**Figure 18:**
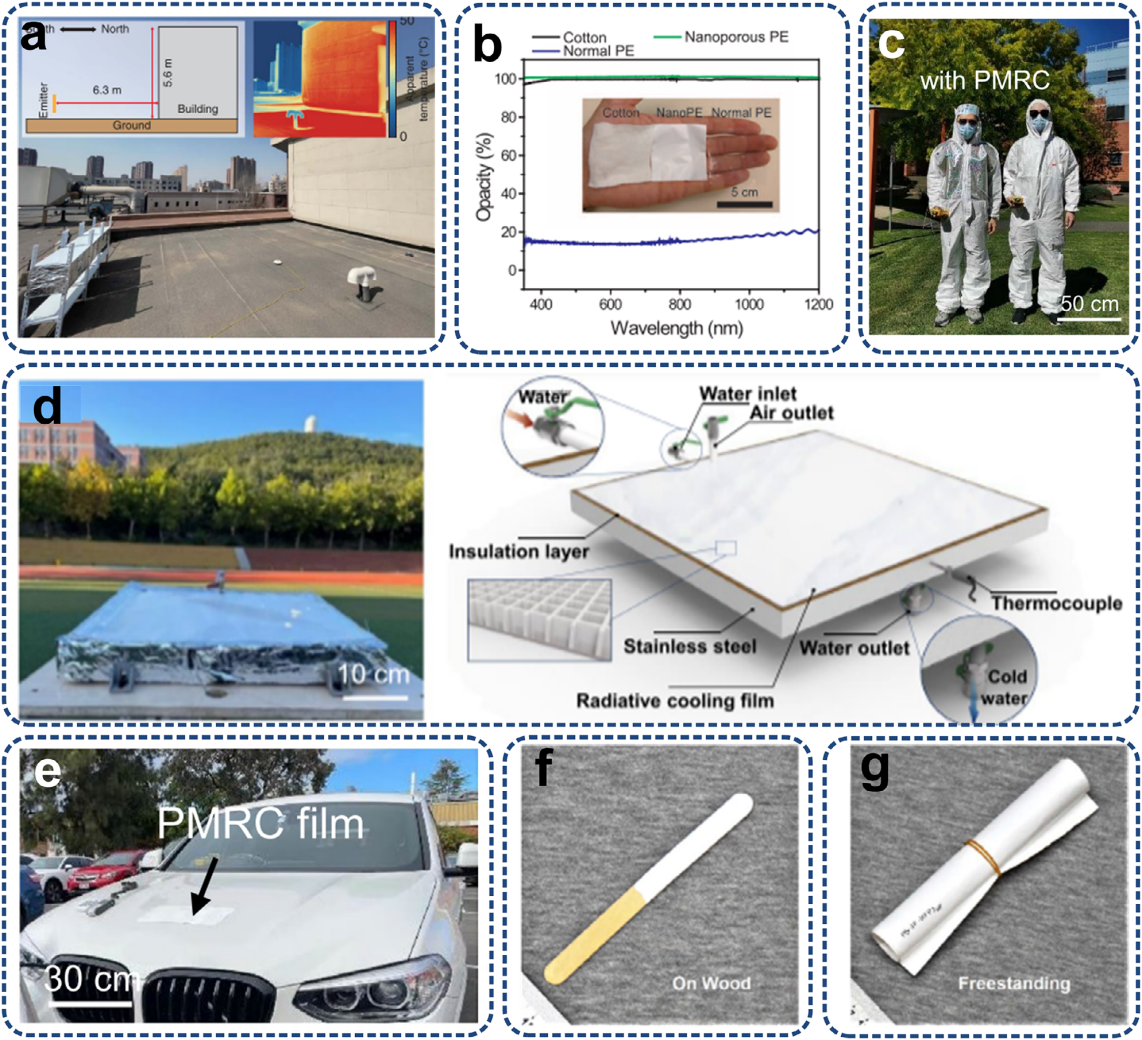
Photograph and/or schematic diagram of various PRC films coated or attached on the surface of a) building, b) hand, c) COVID‐19 protective clothing, d) water cooler system, e) car hood, f) wood, and g) freestanding sheets for tarpaulin‐like designs. Reproduced with the permission.^[^ ^373^, ^376^, ^378^, ^391^ ^]^ Copyright of a, b, f, and g, 2024, 2016, 2018, AAAS. Copyright of c, d, and e, 2023, Springer Nature.

Various application scenarios have been envisioned for cooling radiative films, enabling efficient thermal management, energy conversion, and harvesting without extra electricity. Typical examples include solar cells,^[^ ^390^, ^392^ ^]^ fluid‐cooling systems,^[^ ^376^, ^393^, ^394^ ^]^ buildings,^[^ ^373^, ^395^, ^396^ ^]^ and clothes.^[^ ^397^, ^398^ ^]^ PRC materials initially used for building energy‐saving purposes can be extended to the thermal management of DCs (**Figure** **18**a), which is expected to trigger a revolutionary enhancement in energy efficiency compared to traditional cooling methods employed for large‐scale electronics. For PRC films applied to clothing (Figure 18b), combining this technique will significantly improve heat transfer efficiency as well as reduce costs, given that electronic devices are designed to be wearable and portable. The radiative cooling film proposed previously was applied for medical protective clothing^[^ ^377^ ^]^ (Figure 18c) and water‐cooling devices (Figure 18d) due to its preferable radiative cooling performance. The film was further developed to be magnetically attachable for convenient detachment from steel or iron surfaces (Figure 18e–g),^[^ ^399^ ^]^ which is promising for highly efficient cooling on the surface of electric vehicles or (5G/6G) communication base stations. We have summarized the recent advancement of PRC devices and their outcomes (see Table S2, Supporting Information), covering the transparent and colored PRCs that have recently attracted considerable attention.^[^ ^400^, ^401^, ^402^ ^]^ Earlier results (before 2021) can be found in our previous work.^[^ ^93^ ^]^

### 6.3 Nonreciprocal Thermal Radiation

Nonreciprocal thermal radiation has emerged rapidly as a promising field in thermos‐photonic engineering and energy conversion applications.^[^ ^98^, ^403^ ^]^ Breaking the time‐reversal symmetry in radiative heat transfer enables system design with unique functionalities, such as directional thermal emitters, thermal diodes, and enhanced energy conversion devices. Various physical mechanisms have been explored to achieve nonreciprocity, including MO effects under external magnetic fields, motion‐induced momentum bias, time‐modulated materials, and nonlinear optical effects.^[^ ^100^ ^]^ Recent advances in micro/nanophotonic structures, such as photonic crystals, metasurfaces, and topological materials, have further enhanced the control of nonreciprocal radiation heat transfer at micro‐/nanoscales. The contribution of the quantum Hall effect to NTR by using topological insulators or other quantum materials has been highlighted.^[^ ^99^ ^]^ These developments deepen our understanding of fundamental thermal photonics and pave the way for ultra‐fast transformative applications in energy harvesting, thermal management, and infrared stealth technology.

**Figure 19:**
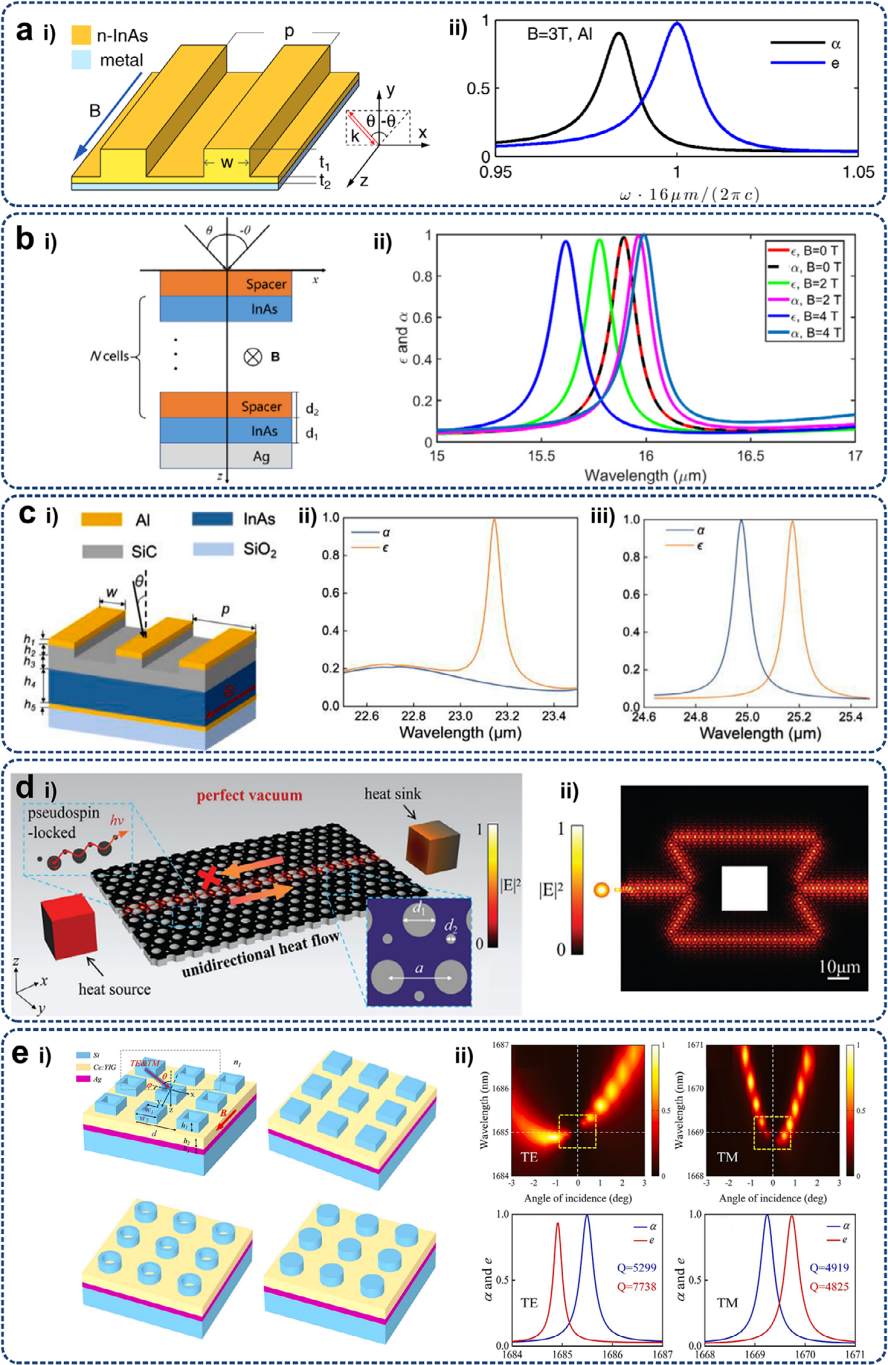
a‐i) A schematic diagram of a photonic crystal structure for the NTR emitter with an extra magnet field. a‐ii) Absorptivity and emissivity spectra for the structure atop an aluminum (Al) substrate. Reproduced with permission.^[^ ^406^ ^]^ Copyright 2014, American Physical Society. b‐i) A Schematic of the multilayer magnetophotonic crystal structure on the silver substrate. b‐ii) The absorptivity and emissivity spectra change with an external magnetic field (*θ* = 60°). Reproduced with permission.^[^ ^414^ ^]^ Copyright 2023, Elsevier. c‐i) Near‐normal NTR of MO grating structure with B = 0.3 T. The spectroscopic performance of the proposed structure with c‐ii) *θ* = 1°and c‐iii) *θ* = 65° (B = 0.3 T). Reproduced with permission.^[^ ^97^ ^]^ Copyright 2024, Elsevier. d‐i) Microscale unidirectional and ultrafast radiative heat transfer was implemented on silicon‐based VPCs, compared to traditional heat conduction. d‐ii) The normalized thermal radiation field intensity distribution demonstrates a specially designed radiation pathway. Reproduced with permission.^[^ ^103^ ^]^ Copyright 2024, Wiley‐VCH. e‐i) Schematic diagrams of four types of unidirectional emitters. e‐ii) The absorption spectra with small angles and narrow‐band spectrum of square ring array structures under TE and TM polarizations, and their spectroscopic performances. Reproduced with permission.^[^ ^415^ ^]^ Copyright 2024, Elsevier.

Violating Kirchhoff's radiation law through MO materials or spatiotemporal metamaterials is the potential for highly efficient engineering thermal radiation,^[^ ^404^ ^]^ which supports nonreciprocal surface modes or guided modes based on transverse‐magnetic (TM) and/or transverse‐electric (TE) polarizations.^[^ ^405^ ^]^ In 2014, Zhu and Fan^[^ ^406^ ^]^ originally proposed a 1D nano‐structured semiconductor comprised of an n‐InAs grating on the Al substrate, based on fluctuational electrodynamics and coupled mode theory, which demonstrates NTR performance with a high external magnetic field (*B* = 3T) (**Figure** **19**a). Furthermore, to illustrate the enhanced effect and nonreciprocal characteristics exhibited by near‐field radiative heat transfer at the micro‐/nano‐scales, detailed theoretical analyses have been conducted based on basic geometric models, such as particle,^[^ ^405^, ^407^ ^]^ cylinder,^[^ ^408^ ^]^ and substrate films.^[^ ^409^, ^410^ ^]^ Furthermore, the enhanced NTR effect based on topological interface states gained considerable attention. It comprised Weyl semimetal films sandwiched by asymmetric topological photonic crystal layers. Chen et al.^[^ ^404^ ^]^ introduced a Monte Carlo tree search algorithm combined with the Bayesian optimization algorithm and the transfer matrix method (TMM) to efficiently design a multilayer (32 layers) film composed of MO material InAs and SiO_2_, which demonstrates a ‐band NTR emitter with an external magnetic field applied. With *B* = 5T, they substantially violated Kirchhoff's radiation across a wide range of incident angles (15°–85°). A similar multilayer heterostructure was proposed by Luo et al.^[^ ^411^ ^]^ They found that coupled topological edge state (CTES) modes can effectively enhance the local field and facilitate perfect multi‐band NTR based on TMM calculations. Wu et al.^[^ ^412^ ^]^ developed a novel multilayer NTR emitter by the TMM and the simulated annealing method. They achieved near‐perfect nonreciprocal radiation with a nonreciprocity of 0.939 at a wavelength of 10 µm and an incident angle of 60° due to the excitation of topological interface states, which was further expanded to strong tri‐band nonreciprocal radiation with an improved nonreciprocity. Zhang et al.^[^ ^413^ ^]^ theoretically investigated a multiscale NTR multilayer structure predicated on Weyl semimetal. The device demonstrated remarkable angle and refractive index detection capabilities across four scenarios. Notably, Shayegan et al.^[^ ^95^ ^]^ demonstrated a direct observation of NTR phenomenon using a guided‐mode resonance coupled to a InAs MO, resulting in an antisymmetric relationship where the magnetic tuning of enhanced emissivity for a given angle correlates with decreased absorptivity.

Introducing an extra magnetic field is promising to achieve nonreciprocal heat transfer since the asymmetric permittivity and/or permeability tensors break the symmetry of the scattering, violating conventional Kirchhoff's law. Wu et al.^[^ ^414^, ^416^ ^]^ explored the potential of realizing intense nonreciprocal radiation within magnetophotonic crystals and deciphering the underlying physical mechanism. They demonstrated a strong NTR with a value close to 0.9 at an incidence angle (*θ*) of 30° (Figure 19b). Chen et al.^[^ ^97^ ^]^ introduced a novel structure of a metal‐SiC grating above an MO film. They adopted a research approach that combines theoretical analysis and numerical simulation using COMSOL Multiphysics to investigate the near‐normal NTR. They explored the impact of multiple factors on NTR performance caused by TM polarization. A strong nonreciprocity performance with a 0.3 T magnetic field and ultra‐small incident angle (*θ* = 1°) was discovered (Figure 19c). Compared to TM polarized‐based nonreciprocal radiation, TE polarization has gradually drawn extensive concern. Recently, Wu et al.^[^ ^417^ ^]^ developed an NTR emitter using Weyl semimetal film with Ag substrate for TE wave. The nonreciprocity was improved by manipulating the incident angle and film thickness. Yang et al.^[^ ^103^ ^]^ proposed a device using silicon‐based valley photonic crystals (VPCs) for ultrafast and unidirectional radiative heat transfer on a microscale, which creates a topological photonic bandgap for TE polarized light under optimized structures. As illustrated in Figure 19d, the design of VPCs was optimized to match the radiation bandwidth of electronic devices, demonstrating the ability to control heat transfer along arbitrary paths by the topological waveguide and immune to defects. This work provided a new approach for thermal management in microelectronics, which is eight orders of magnitude faster than conventional conduction in milliseconds.

The combination of surface micro/nanostructured morphology on photonic crystals facilitates the realization of broadband NTR operated under dual‐polarization conditions (TE and TM). Zou et al.^[^ ^418^ ^]^ proposed a polarization‐independent NTR nanostructure based on silicon cylindrical grating. The nonreciprocal efficiencies of both TE and TM polarizations exceed 90% with optimized structural parameters at a wavelength of 11.763 µm, and high coherence of nonreciprocal efficiency between TE and TM modes was observed. Fang et al.^[^ ^415^ ^]^ designed a near‐infrared dual‐polarization narrow‐band unidirectional emitter composed of periodic annular arrays (square‐column, cylindrical and circular ring, and square ring arrays) (Figure 19e), magneto‐optical medium (Ce: YIG), and Ag reflective layer. Rigorous coupled‐wave analysis (RCWA) and the finite element method were applied to demonstrate high nonreciprocities (>85%) for both TE and TM polarizations at an incident angle of 0.8°. Besides, the dual‐polarization NTR performance of silicon‐based nanopore arrays fabricated on the MO dielectric layer (InAs) and Al layer was investigated.^[^ ^419^ ^]^ It was found that a strong nonreciprocity can be achieved under an incident angle of 49° and a magnetic field of 4T, which was attributed to the mixed resonance excited by guide mode and cavity mode resonances. Furthermore, they demonstrated a dual‐polarization NTR radiator composed of cross‐shaped silicon nanopores, Weyl semimetal, and Ag reflective layer.^[^ ^420^ ^]^ The RCWA simulation results showed that dual polarization nonreciprocity exceeds 80% at an *θ* = 1.6°, and the absorptivity of TE polarization and the emissivity of TM polarization were 98.8% and 97.6%, respectively. It can be established that promising nonreciprocal thermal applications based on thermal nanophotonics have been highlighted due to recent breakthroughs in nanotechnologies. Its high efficiency, stability, and minimization inspired speculation on the application for the heat dissipation of electronics. Nevertheless, the compatibility of TM‐polarized devices used for electronic thermal management is doubtful since the magnetic field will interfere with the transmission and reception of signals, especially in communication devices. Therefore, bi‐anisotropic magnetized lossy metamaterials with strong polarization‐insensitive nonreciprocity gain great potential to be developed as targeted thermal management for chip‐level electronics.

## 7 Design and Manufacturing Methods of Micro/Nanostructures

### 7.1 Design Methods

#### 7.1.1 Conventional Design Methods

**Figure 20:**
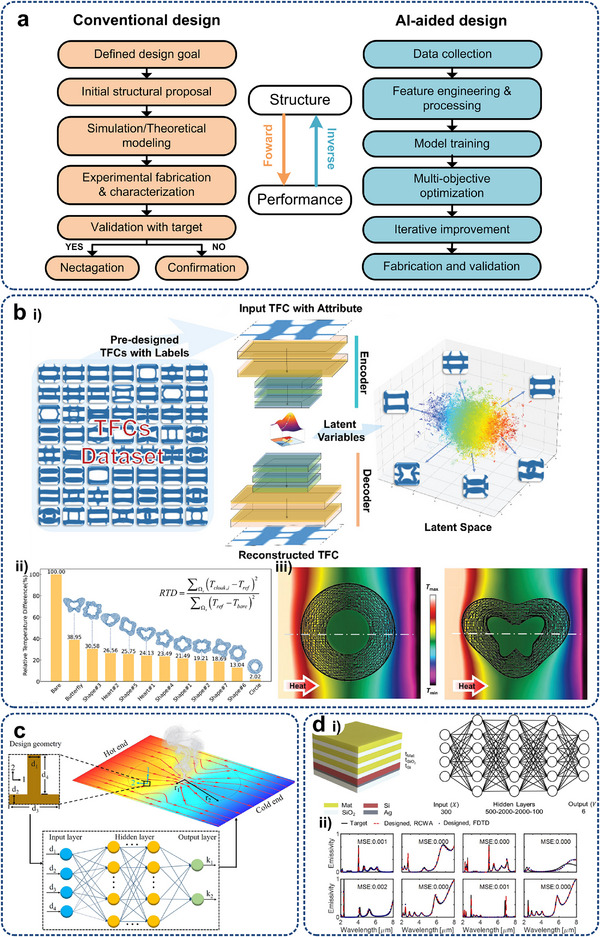
a) Comparison of conventional (Forward) and AI‐aided (Inverse) design processes. b‐i) Schematics of pre‐designed unit cell data set, architecture of the deep generative network, and distribution of training data in latent space. b‐ii) The radiative temperature difference of various designed thermal cloaks. b‐iii) Experimentally measured temperature fields of two thermal cloaks. Reproduced with permission.^[^ ^429^ ^]^ Copyright 2023, Wiley‐VCH. c) Schematic diagram of the optimized design geometry of the thermal concentrator by an ANN and realized the desired thermal concentration effect shown in Figure 4b. Reproduced with permission.^[^ ^183^ ^]^ Copyright 2022, Elsevier. d‐i) Schematic illustration of the multilayer structure using the ANN. d‐ii) Validation of target optical parameters obtained by ANN compared with numerical simulations. Reproduced with permission.^[^ ^430^ ^]^ Copyright 2021, Optical Society of America.

Traditional design methods for micro/nanostructured metamaterials, surfaces, and heat sink architectures in electronic cooling primarily involve analytical modeling, empirical correlations, and numerical simulations derived from hydrodynamic and thermophysical principles.^[^ ^421^ ^]^ Specifically, analytical models are built upon classical heat transfer mechanisms such as Fourier's law of conduction,^[^ ^160^, ^161^, ^422^, ^423^ ^]^ phonon transport theory,^[^ ^46^ ^]^ Newton's law,^[^ ^6^, ^342^, ^352^, ^424^ ^]^ network‐based thermal resistance formulations,^[^ ^353^, ^355^, ^425^ ^]^ flow dynamics theory,^[^ ^227^, ^248^, ^274^ ^]^ radiation transport theory,^[^ ^373^, ^376^, ^399^ ^]^ and other models related to micro/nanoscale heat transfer,^[^ ^233^, ^238^ ^]^ as we discussed in Section 2. Empirical design rules, such as correlations for dimensionless numbers or expressions for fin‐array optimizations, offer practical guidance and have been widely used in industrial applications due to their simplicity and reliability. Numerical approaches, including molecular dynamics,^[^ ^426^ ^]^ finite element methods (FEM),^[^ ^267^, ^351^ ^]^ and finite‐difference time‐domain method (FDTD),^[^ ^93^, ^376^ ^]^ enable detailed exploration of complex geometries and multi‐physical interactions, such as the coupling of multiple heat transfer mechanisms to electrical or magnetic fields.^[^ ^414^, ^415^, ^419^, ^420^ ^]^ However, these methods are computationally intensive, as optimization often relies on parameter sweeps, orthogonal testing, or heuristic search methods, which can be time‐consuming and have limited design scope. Furthermore, bio‐inspired^[^ ^274^, ^427^ ^]^ and fractal‐based approaches^[^ ^428^ ^]^ have also been investigated, aiming to replicate efficient natural flow or radiation patterns. It should be noted that those conventional design strategies follow a forward design process, which transforms physical models into mathematical formulations via reasonable assumptions, and evaluates the predictive performance based on simulations or experiments. However, their outcomes are constrained by human intuition and iterative trial‐and‐error processes^[^ ^183^ ^]^ (**Figure** **20**a).

#### 7.1.2 Machine Learning aided Optimization

AI has revolutionized material research by overcoming limitations of traditional approaches.^[^ ^431^ ^]^ A good example is employing machine learning for the forecasting of unprecedented material characteristics, rather than relying heavily on trial‐and‐error or theoretical models.^[^ ^432^ ^]^ Among various machine learning models, e.g., decision trees, support vector machines (SVM), and artificial neural network (ANN), deep learning (DL) has emerged as an auspicious approach in this thriving field and has found versatile applications related to thermal and energy management systems, including thermoelectric cooling,^[^ ^431^, ^433^, ^434^, ^435^, ^436^ ^]^ processors,^[^ ^437^, ^438^ ^]^ battery thermal management,^[^ ^439^, ^440^ ^]^ fuel cell,^[^ ^441^, ^442^ ^]^ and hydrogen storage.^[^ ^443^ ^]^ In terms of theoretical analysis and model modification of heat transfer, DL methods demonstrate high efficiency and accuracy in various aspects (thermal boundary resistance, boiling heat transfer, thermal radiation, etc).^[^ ^444^, ^445^, ^446^, ^447^ ^]^ As a result, facilitating the structural and process optimization of metamaterials (thermal or hydrodynamics) via DL algorithms has attracted significant attention. Zhu et al.^[^ ^448^ ^]^ presented a comprehensive review focusing on AI‐aided metamaterial design. This timely report specifically explored three key aspects of machine learning applications, i.e., optimization of metamaterials to achieve target properties, integration of discriminative models with optimization algorithms to improve computational efficiency, and generative models used for structural design^[^ ^449^ ^]^ (Figure 20b–d). Furthermore, DL methods have been applied in the design and optimization of microfluidic systems (hydrodynamic metamaterials) to fabricate microstructures with tailored morphologies and heterogeneous material compositions.^[^ ^450^, ^451^, ^452^, ^453^ ^]^ This technique can be further extended to microrobots^[^ ^454^ ^]^ and engineered fibrous tissue constructs,^[^ ^455^, ^456^ ^]^ highlighting its versatility in biomedical and robotic applications. For instance, Yang et al.^[^ ^453^ ^]^ proposed an AI‐driven microfluidic flow programming framework (CeyeHao) that integrates a custom neural network (CEyeNet) with hierarchically assembled obstacles (HAOs). This system overcomes the limitations of conventional microfluidics by enabling highly configurable, complex flow manipulation within the Stokes flow regime. CEyeNet predicts flow transformations with up to 2700 times faster speed than CFD while maintaining high accuracy validated by experiments (90%). It supports both real‐time human‐guided design and automatic inverse design, allowing for the creation of intricate geometric and artistic flow profiles with experimental fidelity. Consequently, different from traditional methods, AI‐aided approaches represent a fundamentally different paradigm that leverages large datasets generated from experiments or high‐fidelity simulations. They are capable of creating novel micro/nanostructures beyond conventional human intuition (Inverse process), and facilitate multi‐objective optimization, as illustrated in Figure 20a. The primary trade‐off, however, lies in the lack of physical interpretability. **Table** **3** summarizes the differences between AI‐assisted and traditional strategies, providing a reference for the next‐generation design methods.

**Table 3:** Characteristics of traditional and AI‐aided design methods.

	Traditional methods (Physics‐based)	AI‐assisted methods (Data‐driven)
Efficiency	Low. Iterative simulations/experiments required	High. Fast prediction and automatic optimization
Interpretability	Strong. Clear physical mechanisms	Limited. “black‐box” predictions
Design scope	Narrow‐Constrained by assumptions and intuition	Broad. Capable of exploring complex, high‐dimensional cases
Innovation	Incremental. Experience‐driven	Potentially. novel‐Beyond conventional intuition

### 7.2 Fabrication Methods

#### 7.2.1 Rapid 3D Printing

**Figure 21:**
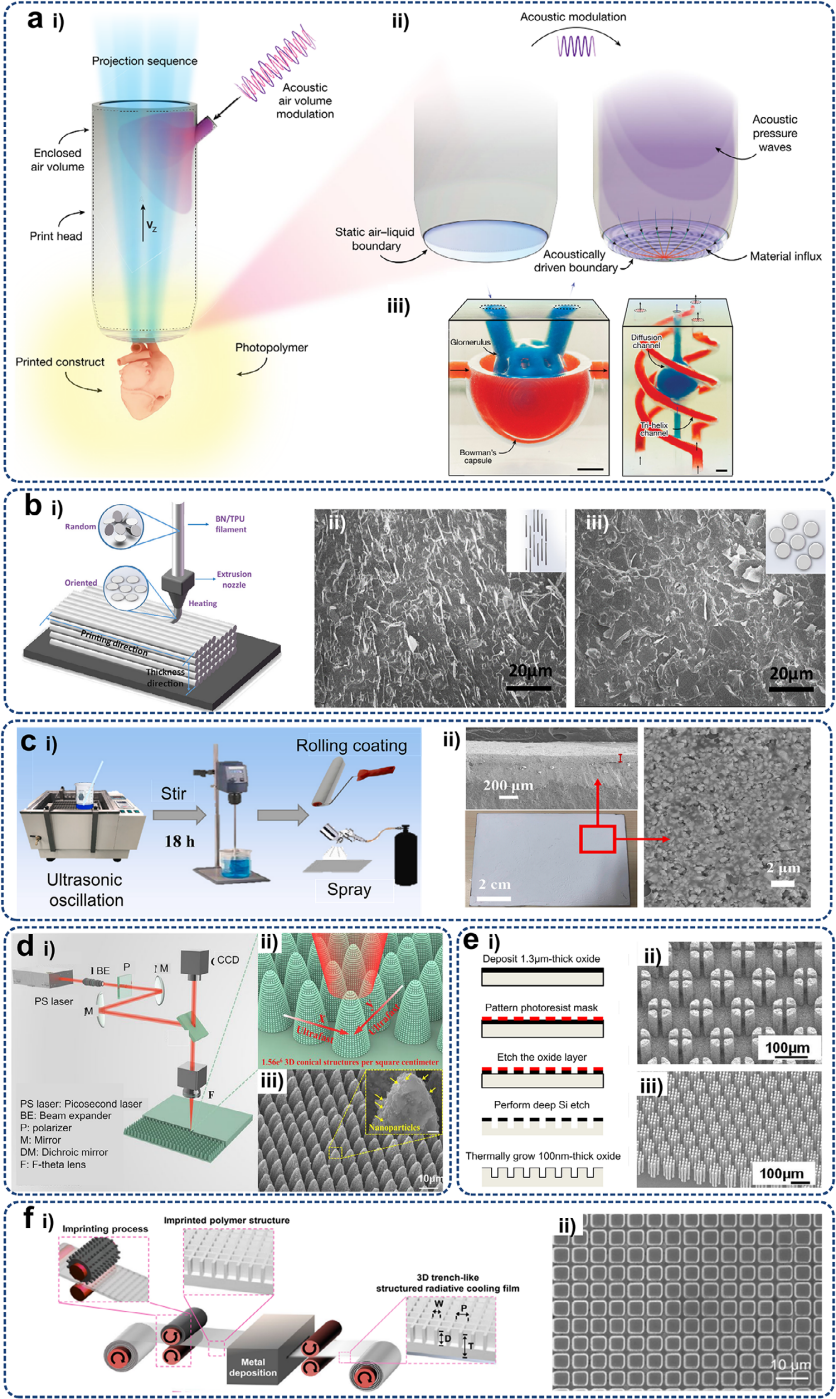
a‐i) Schematic illustration of the DIP shows an air‐liquid boundary forms on a partially submerged print head. a‐ii) Acoustic manipulation of the internal air volume in the print head. a‐iii) Printed samples fabricated by DIP include Bowman's capsule and Tri‐helix structure. Scale bar 2 mm (left) 1 mm (right). Reproduced with permission.^[^ ^474^ ^]^ Copyright 2024, Springer Nature. b‐i) A schematic of fused deposition modeling 3D printing. SEM images of cross‐sections for the FDM 3D printed hBN/TPU samples in b‐ii) printing, and b‐iii) thickness direction. Reproduced with permission.^[^ ^475^ ^]^ Copyright 2021, Elsevier. c‐i) Preparation process of the Bio‐PRC film via surface coating. c‐ii) SEM images of the Bio‐PRC coating. Reproduced with permission.^[^ ^387^ ^]^ Copyright 2019, Elsevier. d‐i) A schematic of 3D micro/nanostructures fabricated by picosecond laser processing. d‐ii) 3D and d‐iii) SEM images of microstructures. Reproduced with permission.^[^ ^476^ ^]^ Copyright 2024, Springer Nature. e‐i) DRIE process flow used to fabricate the silicon wicks. SEM images of e‐ii) clusters of Si cylinders and e‐iii) pied Si cylinders. Reproduced with permission.^[^ ^477^ ^]^ Copyright 2012, Elsevier. f‐i) Schematic of steps in roll‑to‑roll imprinting for preparing 3D structured PRC films. f‐ii) SEM of polymer metamaterial used for radiative cooling. Reproduced with permission.^[^ ^376^ ^]^ Copyright 2023, Springer Nature.

Rapid 3D printing allows straightforward fabrication of complex architectures, which has been recognized as an efficient technique for various applications, including aerospace,^[^ ^457^ ^]^ automobile industry,^[^ ^458^ ^]^ microfabrication,^[^ ^459^ ^]^ and architectural modeling,^[^ ^460^ ^]^ especially in biomedical field.^[^ ^461^, ^462^, ^463^ ^]^ Light‐based printing technologies, featuring high solution and surface quality, can meet the requirement of micro/nano‐manufacturing. It can be further classified into two‐photon polymerization,^[^ ^464^ ^]^ projection micro‐stereolithography (PµSL),^[^ ^465^, ^466^ ^]^ and volumetric printing.^[^ ^462^, ^467^, ^468^ ^]^ Loterie et al.^[^ ^469^ ^]^ suggested a tomographic volumetric printing that solidifies 3D objects by irradiating the volume of liquid photopolymer. In their research, the photopolymerization kinetics can be accurately controlled in a short time (< 30 s), and the specimen with sub‐millimeter scales can be fabricated by optimizing the étendue of the source. Toombs et al.^[^ ^470^ ^]^ developed a microscale computed axial lithography (*µ*‐CAL) technology in silica glass 3D manufacturing. This technology fabricated a variety of microstructures by tomographically illuminating a photopolymer‐silica nanocomposite and sintering process, including three‐dimensional microfluidics with an internal diameter of 150 µm and complex high‐strength trusses and lattice structures with a minimum feature size of 50 µm. Hahn et al.^[^ ^471^ ^]^ presented a light‐sheet 3D microprinting technology based on two‐color two‐step absorption. It was comprised of a specific photo‐initiator and continuous‐wave laser diodes, which combined image projection with optical nonlinearity to achieve high‐speed and high‐resolution printing. The peak printing rate can reach 7 × 10^6^ voxels/s at a voxel volume of 0.55 µm^3^. Geng et al.^[^ ^472^ ^]^ investigated an ultrafast multi‐focus 3D nanofabrication technology based on two‐photon polymerization (TPP) and a digital micromirror device (DMD) scanner at a high resolution (≈500 nm). Experiments demonstrated its capability in fabricating complex structures such as 3D trusses and woodpile structures. Compared with the traditional TPP process, it significantly improved production and enhanced focus position and laser dosage control. Vidler et al.^[^ ^473^ ^]^ explored the digital light processing (DLP)‐based PµSL for ultrahigh resolution. The technology can achieve an optical resolution of 1 µm on millimeter‐scaled samples through optimized exposure compensation, resin formulation, cure depth, and in‐plane feature resolution. Experiments showed that the small pixel size is crucial for printing high‐fidelity microparts, and this technology can batch process multiple parts and is widely applicable in many fields. Recently, the authors proposed a novel dynamic interface printing (DIP), which utilized an acoustically modulated, constrained air‐liquid boundary to rapidly fabricate 3D structures with high speed and a wide range of materials (**Figure** **21**a).^[^ ^474^ ^]^ Application of this technique spans from creating complex structures in hard acrylates to biologically relevant models with high cell viability.

#### 7.2.2 Fused Deposition Modeling

Fused deposition modeling (FDM) has been widely employed in manufacturing specially designed micro‐/nanostructures. A heated nozzle is used to melt and extrude a thermoplastic filament to build the object layer by layer, which is simple, has high material flexibility, and is suitable for rapid prototyping. However, this technique may have limitations in resolution and surface‐processing scenarios. Liu et al.^[^ ^475^ ^]^ prepared thermoplastic polyurethane samples filled with highly thermal conductive hexagonal boron nitrides platelets using FDM (Figure 21b). The hBN was found to be directional, and the printing process was caused by the shearing effect of the FDM technique. Li et al.^[^ ^478^ ^]^ employed high‐resolution 3D printing to fabricate sophisticated structures with peristome‐mimetic surfaces, and the PDMS was used as the replica. Temperature‐responsive gels, Poly N‐isopropyl acrylamide, were used to mimic the opening and closing state of the shorebird's beak.

#### 7.2.3 Surface Coating

The research on surface coating is advancing in multiple aspects. For instance, technological innovations like nanocoating and 3D printing coating have been emphasized. New materials are emerging, and applications are expanding to the aerospace, automotive, medical, and electronics fields. Performance and theoretical studies are ongoing to improve durability and understand the relationship between microstructure and properties. Chae et al.^[^ ^366^ ^]^ sputtered the ethylene tetrafluoroethylene (ETFE) films with a silver target to form a 200 nm‐thick silver backing layer, and the thickness of the two types of films are 50 and 100 µm, respectively. A high transmittance (90.5%) and low absorption (4.2%) of the ETFE film in the solar band were observed after coating. Cheng et al.^[^ ^387^ ^]^ fabricated the Bio‐RC coating using a simple roller coating method that included ball‐milling, weighing, ultrasonic oscillating, stirring, and drying. According to microscopic observations, the Bio‐RC coating with about 100 µm thickness was tightly attached to the substrate, as shown in Figure 21c. Zhai et al.^[^ ^383^ ^]^ randomly embedded resonant polar dielectric microspheres (SiO_2_) in a polymeric matrix. The film was backed with a 200‐nm‐thick silver coating to realize high infrared emissivity and solar irradiance reflection. The film was fabricated in 300‐mm‐wide sheets at a rate of 5 mmin^−1^, demonstrating the high efficiency of this technique. Another literature mentioned the fabrication process of a biomimetic hydrophilic foam structure via coating the hydrophilic polyvinyl alcohol foam with photothermal polypyrrole.^[^ ^479^ ^]^

#### 7.2.4 Deep Reactive Ion Etching

Compared with traditional microfabrication processes, deep reactive ion etching (DRIE) has been popular due to its high processing efficiency, accuracy, and compatibility. Zhu et al.^[^ ^390^ ^]^ developed a silica photonic crystal layer by etching 10‐µm‐deep holes into a 500‐µm‐thick fused silica wafer with DRIE techniques, with precise size and geometry. Zou et al.^[^ ^382^ ^]^ fabricated a radiative metasurface with the unit cell in microscales (2.3 × 1.55 × 1.5 µm). Adera et al.^[^ ^350^ ^]^ conducted the fabrication of the micropillar array via DRIE, which was used as a capillary wick in the thermal management systems of microprocessors. The pillars' width, height, and distance varied between 5–12, 30–90, and 12–20 µm, respectively. The microscale Tesla valve array was built via DRIE for the unidirectional flow control.^[^ ^106^ ^]^ Patterned pillar arrays along the tesla valve channels were demonstrated with the diameter and spacing of pillars at 30 and 10 µm. A porous‐wall microchannel was designed by Xu et al.^[^ ^110^ ^]^ for a high‐frequency ‘eye‐blinking’ interface oscillation. The minimum spacing between micro‐fins was only 5 microns. Other typical structures, including Piranha pin fin,^[^ ^103^ ^]^ droplet,^[^ ^266^ ^]^ pied Si cylinders,^[^ ^182^ ^]^ and pillar array,^[^ ^2^ ^]^ illustrate the reliability of DRIE in processing within the precision range from micron to sub‐micron (Figure 21d).

#### 7.2.5 Laser Micro‐Milling

Laser micro‐milling has made significant progress in the field of micro/nanostructure processing. It offers relatively high precision and flexibility, enabling the creation of complex microstructures. This technology has been widely applied in areas such as electronics, optics, and biomedical engineering. With continuous research and development, its capabilities are expected to be further expanded. Su et al.^[^ ^422^ ^]^ developed a metamaterial by mechanical punching and laser micromilling with 30 × 6 holes evenly punched in a 15 × 3 cm^2^ area. Liao et al.^[^ ^343^ ^]^ processed the microfabrication of nucleated pin‐fins in microchannels to experimentally explore their boiling heat transfer performance. The center pore size, opening size, and height were 60, 45, and 100 µmwa, respectively. Deng et al.^[^ ^344^ ^]^ built micro cone pin fins on the bottom surface of parallel microchannel arrays using the laser micromilling method. The line spacing was set as 5 µm, and the laser was set to produce 100 ns pulses with a 1064 nm wavelength at 20 kHz. Zong et al.^[^ ^253^ ^]^ investigated the boiling performance within microchannels, whose walls were designed as porous structures, etched with micro pin‐fin arrangement. The smallest design spacing between fins was only 5 microns. To achieve ultra‐high thermal convection boiling, Palko et al.^[^ ^104^ ^]^ specially designed laser‐etched polycrystalline diamond microchannels integrated with template‐fabricated microporous copper. High aspect ratio extended surfaces were achievable with the laser (1064 nm) micromachining. This technique was also used to effectively control the hydrophilic and hydrophobic properties of the surface by simply processing arrayed grooves.^[^ ^480^ ^]^

#### 7.2.6 Pico‐/Femtosecond Laser Fabrication

Femto‐/picosecond lasers have experiencing significant development, especially in the past decade. They offer extremely high precision and minimal thermal damage, making them valuable in various fields connected with micro/nano‐manufacturing. However, low processing efficiency, high system complexity, and high equipment cost pose challenges to promoting this technology.^[^ ^481^ ^]^ Liu et al.^[^ ^476^ ^]^ reported an ultra‐fast Picosecond laser processing for 3D conical structures on zinc anodes. The spot diameter, wavelength, and pulse duration are 10 µm, 355 nm, and 8 ps (Figure 21e). Hanks et al.^[^ ^482^ ^]^ proposed a new device for the thermal management of high‐flux electronics. A 600‐nm‐thick nanoporous membrane with 110 nm pores was suspended over a liquid supply network of microchannels to realize high capillary pressure with enhanced permeability. Gao et al.^[^ ^483^ ^]^ explored the application of femtosecond laser to machine parallel grooved structures on the surface of silicon nitride ceramics to tackle their high brittleness and low fracture toughness. The spot size in the focal plane was only 13 µm with the wavelength of 1035±5 nm.

#### 7.2.7 Photolithography

Photolithography has become a promising technique for fabricating nanoscale features with high resolution and throughput. Currently, research efforts are focused on further improving the resolution and fidelity of nanoimprint lithography and exploring its applications related to semiconductors, optics, and biotechnology. Despite its strengths, wickedness such as template fabrication, alignment, and process control still needs to be addressed to unleash the potential. Fernández et al.^[^ ^484^ ^]^ proposed a hybrid hierarchical surface realized by combining lotus and petal surface structures with different wetting states fabricated by nanoimprint lithography. The molds were made by casting the PDMS prepolymer against the relief structure of the different silicon substrates. Nanopillars and nanospikes with a 200‐700 nm height can effectively tailor the adhesiveness of water droplets. Woodcock et al.^[^ ^251^ ^]^ patterned Piranha Pin Fins as small as 5 µm on two 400‐µm‐thick double‐polished silicon wafers by nanoimprint and photolithography. Cai and Bhunia^[^ ^348^ ^]^ developed a micropillar array evaporator for thin film evaporation to achieve high‐density heat removal. The minimum diameter of the perpendicular cylindrical silicon pillar was designed to be 10 µm. The monolayer graphene thermal rectifiers were prepared by Wang et al.,^[^ ^173^ ^]^ who found that applying electron beam lithography and oxygen plasma etching can form the suspended graphene ribbon with nanoparticles (≈200 nm) into any desired shape. Bryche et al.^[^ ^485^ ^]^ designed and fabricated 60 nm‐thick gold nanocrosses arrays by electronic beam lithography atop 30 nm‐thick gold films with a 2.5 nm thick Ti layer. It showed that heat can be selectively and ultra‐fast transferred through either of the two arms of the novel nanocrosses. Jia et al.^[^ ^374^ ^]^ employed a combination of e‐beam lithography and an e‐beam evaporation process to develop the CMM pillars for anisotropic radiative heat transfer. The optimized top and bottom diameters of CMM were 1.05 and 1.75 µm, respectively, which allowed highly selective emission almost overlapping the atmosphere window, where the peak emissivity reached 99% at 4.9 µm. The microstructures were generated by filling the evaporated germanium and aluminum into PMMA molds. Furthermore, they creatively proposed the manufacturing‐friendly roll‐to‐roll printing technique.^[^ ^376^ ^]^ The photo‐imprinting process produced 5‐µm‐thick PET films with 3D trench‐like microstructures (Figure 21f).

In summary, while methods like femtosecond laser and vat photopolymerization offer high precision and material versatility, they often come with trade‐offs like lower throughput and higher costs. Conversely, more straightforward methods like DIW and FDM are more cost‐effective but provide lower resolution and manufacturing speed. Given the rapid development of nanoscale metamaterials in non‐reciprocal heat transfer, surface textures at the nano‐to‐submicron scales have become paramount. However, it is challenging for traditional strategies, such as ion etching, surface coating, and fused deposition, to manipulate uniform and regular micro‐nanostructures. Therefore, laser processing and lithography combined with imprinting or surface deposition to improve their accuracy and efficiency are highly recommended. The strengths and weaknesses of the mentioned manufacturing methods can be found in **Table** **4**.

**Table 4:** Comparison of micro/nanostructures manufacturing methods.

Method	Strength	Weakness	Precision	Refs.
Rapid 3D printing	Personalization and customization. Rapid Prototyping. Portability and mobility. Reduced Waste By‐products.	High cost with high quality. Printing speed needs improved. Printing size limitations. Material limitations.	micron to sub‐millimeter	[457, 462, 468, 473, 474, 486]
Fused deposition modeling	Easy to use and affordable. Capable of large‐scale models. High accessibility.	Low accuracy and mechanical properties. Not suitable for fabrication at nanoscale (resolution limitations)	millimeter to sub‐millimeter	[475, 478]
Surface coating	High flexibility (various materials). Efficiently enhanced the surface properties (optical properties, corrosion or wear resistance, adhesion etc.) Low cost and simple process.	Thickness limitations that may affect the performance of micro‐/nano‐structures. May introduce additional mechanical stress on the underlying structures. Difficult to perform 3D structures.	sub‐micron to micron	[366, 383, 387, 479]
Deep reactive ion etching	Achieve rapid removal of material. Precise control of shape and dimensions. High flexibility (various materials).	High equipment cost. May cause some surface damage or defects on the etched material	sub‐micron to micron	[6, 250, 252, 382, 390, 477, 487]
Laser micromilling/caving	Achieve fine resolution as the microscale. Relatively high processing efficiency compared with traditional processing methods Apply on various materials, e.g., sapphire, plastic, glass, ceramics, aluminum, Good flexibility by controlling laser parameters Without generating mechanical extrusion or mechanical stress	High equipment cost Professional technical knowledge and operating experience are needed Not suitable for large‐depth milling The surface roughness still needs to be further improved	sub‐micron to micron	[104, 253, 343, 344, 422, 480]
Femtosecond laser nanoprinting (FSLNP)	Extremely high precision and resolution. Minimal heat‐affected zones. Ability to process nearly all materials, including metals, semiconductors, and biological tissues. 3D structuring. Reducing contamination and wear on equipment.	Low efficiency for large‐scale production Complex systems and expensive equipment are required. Difficulties in monitoring and controlling electron dynamics at the femtosecond scale	nanometer	[476, 481, 482, 483, 488]
Nanoimprint lithography	Achieve high‐resolution nanoscale pattern transfer Perform large area imprinting to improve production efficiency Simple process without a complex optical system and photoresist	High‐quality template fabrication is difficult and costly Defects such as bubbles and residual layers may occur. High requirements on the imprinting materials (fluidity and plasticity)	nanometer	[173, 251, 348, 374, 376, 399, 484, 485]

## 8 Application of Micro/Nanostructures in Electronic Thermal Management

Micro/nanostructures offer practical benefits in advanced electronic thermal management systems, such as enabling device miniaturization, reducing contact resistance, increasing the heat exchange surface area, and enhancing heat transfer rates.^[^ ^489^, ^490^, ^491^, ^492^, ^493^ ^]^ Special concern has been paid to the performance of TIMs (heat dissipation from the chip) and heat sinks (heat diffusion to the environment) for thermal management in chip‐level devices (heat source), as discussed in Section 2.2. Therefore, this section will focus on the practical applications of these two components in electronic cooling to inspire the relevant application potential of micro/nanotechnologies.

### 8.1 Thermal Interfacial Material

**Figure 22:**
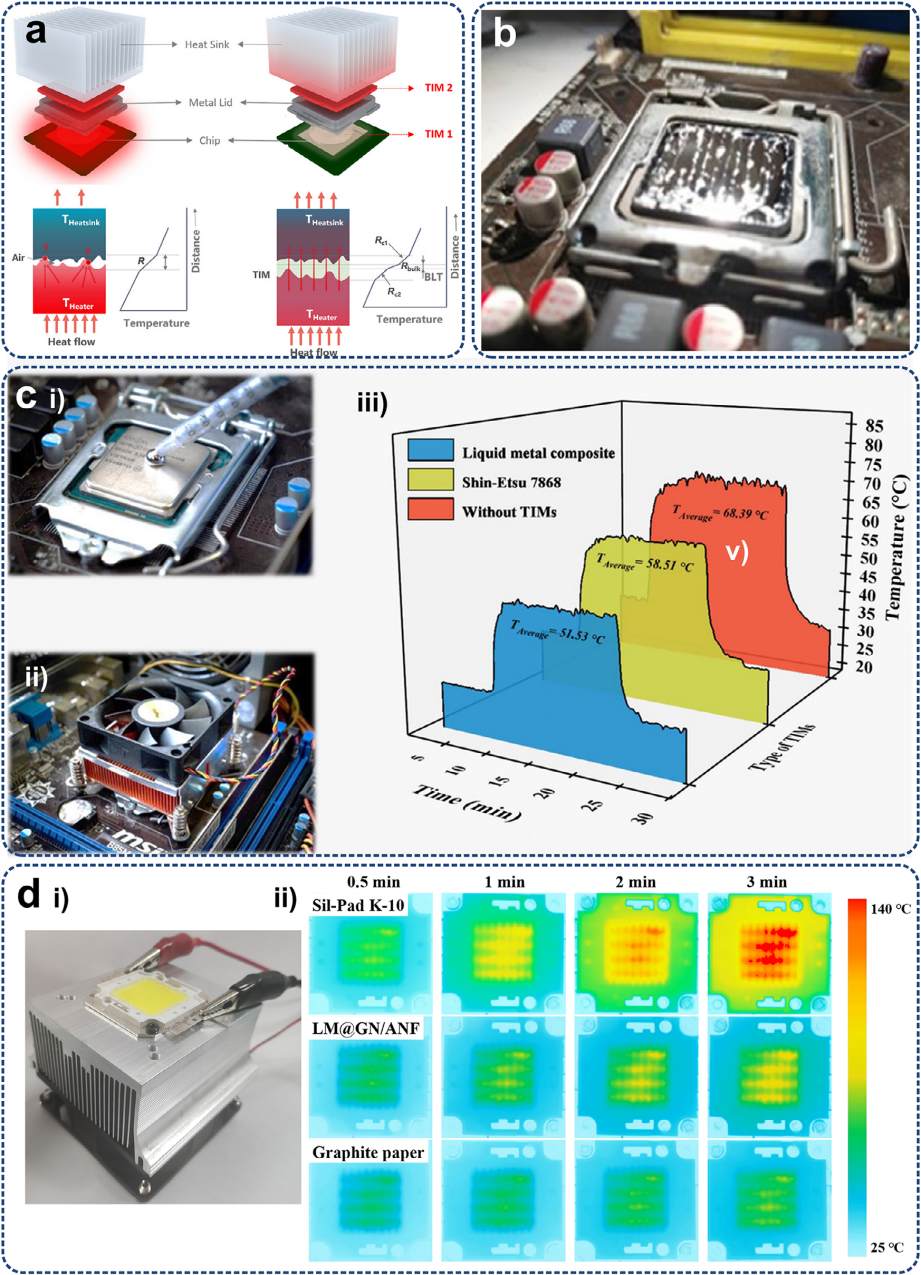
a) Schematic diagram of chips with (right) or without TIMs (left) that demonstrates its heat dissipation performance. Reproduced with permission.^[^ ^521^ ^]^ Copyright 2024, Wiley‐VCH. b) Demonstration of TIM (LM) coated on chip surface after destructing the oxide layer. Reproduced with permission.^[^ ^522^ ^]^ Copyright 2024, Elsevier. c‐i) and c‐ii) Photographs of heat dissipation tests of CPU with LMPCM and the traditional cooling strategy. c‐iii) CPU core temperature comparison using two types of LMPCMs and without the TIM. Reproduced with permission.^[^ ^523^ ^]^ Copyright 2023, Wiley‐VCH. d‐i) LED cooling system with TIM sandwiched between the LED and the heat sink. d‐ii) Infrared images of the LED cooling system with different TIMs. Reproduced with permission.^[^ ^524^ ^]^ Copyright 2024, Wiley‐VCH.

TIMs are critical materials designed to fill gaps at solid interfaces and enhance thermal conduction through the interfaces.^[^ ^43^, ^494^, ^495^ ^]^ Specifically, the microscopically rough contact surfaces between components and heat sinks lead to low‐thermal‐conductivity air occupying the gaps within electronic devices, significantly increasing interfacial thermal resistance and impeding heat transfer.^[^ ^496^ ^]^ By filling these gaps, TIMs can effectively reduce interfacial thermal resistance (ITR) and improve heat dissipation efficiency, which is crucial for the stable operation of light‐emitting diodes, quantum cascade lasers, phase‐change memories, thermoelectric devices, wearable technologies, and photovoltaic cells^[^ ^497^, ^498^, ^499^ ^]^ (**Figure** **22**a). Common TIM types include thermal greases, thermal pads, and phase‐change materials. The underlying physics of TIM refer to continuous practical and theoretical development of ITR, as previously reviewed by Chen et al.,^[^ ^43^ ^]^ which has evolved from continuum theory (i.e., acoustic mismatch theory^[^ ^500^, ^501^, ^502^ ^]^ and diffused mismatch theory^[^ ^503^, ^504^, ^505^, ^506^ ^]^) to atomistic approaches, such as lattice dynamics,^[^ ^507^, ^508^ ^]^ atomistic Green's functions,^[^ ^509^, ^510^ ^]^ MD,^[^ ^511^, ^512^, ^513^ ^]^ and Boltzmann transport/Monte‐Carlo solvers.^[^ ^514^, ^515^ ^]^ The former is suitable for predicting solid‐solid ITR at low temperatures (<30 K), while the latter enables capturing factors such as lattice mismatch, anharmonicity, and interfacial disorder. Experimental tools include the pump‐probe thermoreflectance technique, the thermal bridge method, and the electron‐beam self‐heating method, which has been widely testified in studying various interfaces.^[^ ^516^, ^517^, ^518^ ^]^ Challenges and opportunities of TIMs lie in updating the fundamental theory of ITR (phonon characteristics),^[^ ^519^, ^520^ ^]^ development of sustainable TIMs, and AI‐aided TIM design.^[^ ^449^, ^521^ ^]^

Recently progress on TIMs has been focused on liquid metals and their composites to bridge the contact upper and lower surfaces in electronic devices, enhancing the thermal conductivity and avoiding thermal stress problems.^[^ ^222^, ^524^, ^525^ ^]^ Zheng et al.^[^ ^523^ ^]^ developed a low‐temperature solidifiable EGaInSn/Cu composite, which achieved an ultrahigh thermal conductivity of 86.7 W(m·K)^−^ and a shear strength of around 13.7 MPa (comparable to low‐temperature solders). The thermal and mechanical properties remain stable in CPU cooling after 1000 thermal cycles (0–90°C), outperforms commercial thermal grease by 7 °C. Zheng et al.^[^ ^523^ ^]^ proposed gallium‐based LMPCM with 55% liquid metal and 15% Cu achieved a thermal conductivity of 3.94 W/(m·K) and a Young's modulus of 699 kPa. The TIM maintained stable properties over 200 temperature cycles and performed well in large‐size LED applications. Yan et al.^[^ ^522^ ^]^ experimentally investigated Ga‐In binary and Ga‐In‐Sn ternary LM‐TIMs with different copper microparticle compositions, as shown in Figure 22b. The LM‐TIMs exhibited good thermal conductivities of 29.6‐38.5 W(m·K)^−1^ at 80–120°C. Luo et al.^[^ ^524^ ^]^ proposed LM@GN/ANF films fabricated by graphene nanosheets (GN) coated LM nanodroplets combined with aramid nanofibers (ANF), which prevented LM aggregation and formed a continuous thermal network. The film with 32.5 vol.% LM@GN achieved a high thermal conductivity (5.67 W·m^−1^·K^−1^), a 24.5% reduction in Young's modulus of (1209.6 MPa) and high fracture energy (202.79 Jm^−2^) via LM deformability, suitable for wearable devices and soft robots (Figure 22d). Liu et al.^[^ ^526^, ^527^ ^]^ prepared a new type of LM@CNT/SR composites by coating In_51_Bi_32.5_Sn_16.5_ with CNTs, and formed with silicone rubber (SR), achieving a thermal conductivity of 1.37 W(m·K)^−1^. Inspired by tree growth rings, vertically aligned graphene films (VAGF) were embedded for macro‐level enhancement. The LM@CNT /SR/VAGF composite showed excellent stability after ≈2000 cycles and reduced LED temperature by 53.8 °C. Meanwhile, they introduced an LM (In_51_Bi_32.5_Sn_16.5_) microcapsule encapsulated by graphene oxide (GO). The thermal conductivity made from LM@GO particles and silicone rubber (SR) is increased by 882.1 % compared to pure SR, and a temperature reduction of 8 °C compared to the thermal grease was obtained using for LED chip cooling.^[^ ^527^ ^]^

### 8.2 Heat Sink

The optimized design and fabrication of heat sinks are crucial to meeting the increasing thermal management demands of electronic devices, requiring multifaceted consideration, including desired heat transfer performance, lightweight, compactness, and mechanical reliability.^[^ ^144^, ^428^ ^]^ Structurally, traditional heat sinks were designed as plate, microchannel, finned (straight, wavy or spiral), pin‐fin, and base‐fin configurations.^[^ ^1^, ^528^ ^]^ Common materials include Al, Cu, and composite materials such as graphite or graphene‐enhanced alloys.^[^ ^24^, ^529^ ^]^ According to their application scenarios, heat sinks can operate via passive natural convection, active forced convection with fans or pumps, and advanced liquid/two‐phase systems.^[^ ^4^, ^530^ ^]^

**Figure 23:**
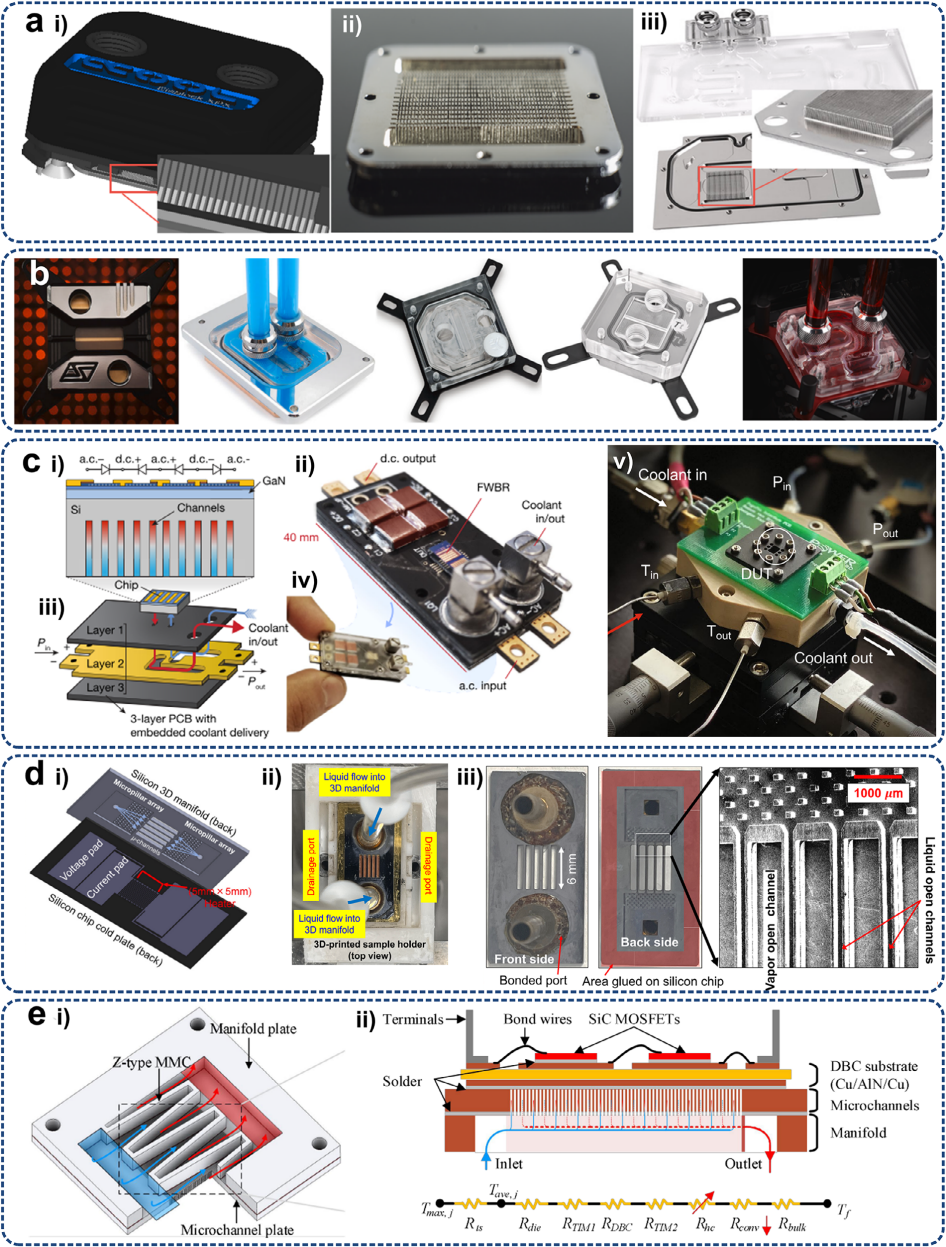
Photograph of microchannel heat sinks used for commercialized microchips, including a‐i) Alphacool, a‐ii) Swiftech, and a‐iii) Thermaltake. Reproduced with permission.^[^ ^531^ ^]^ Copyright 2024, Elsevier. b) Several types of inlet/outlet micro heat sinks. Reproduced with permission.^[^ ^531^ ^]^ Copyright 2024, Elsevier. c‐i) Schematic diagram of a.c.‐d.c. converter with liquid‐cooled power IC based on four GaN power Schottky barrier diodes integrated with a single chip. c‐ii) Structure illustration of the coolant supplementary embedded in the PCB. c‐iii) Photograph of the converter with the thermal management devices. c‐iv) Picture of thermal performance testing section. Reproduced with permission.^[^ ^4^ ^]^ Copyright 2020, Springer Nature. d‐i) A schematic of the Si‐based microchannel manifold and cold plate in the backside view. d‐ii) A photo of the micro‐cooler was placed on the sample holder. d‐iii) Pictures of the front and back sides of the micro‐cooler, as well as the SEM image, show the pillar array, open liquid delivery, and vapor exit channels. Reproduced with permission. Copyright 2024, Elsevier. e‐i) A schematic showing details of TZMMCs in section view. e‐ii) The package illustration and its thermal resistances of manifold microchannels. Reproduced with permission.^[^ ^131^ ^]^ Copyright 2024, IEEE.

The practical application of single‐/two‐phase heat sinks on chip‐level systems has gradually emerged in recent decades.^[^ ^5^ ^]^ Rangarajan et al.^[^ ^531^ ^]^ comprehensively reviewed the research on on‐chip embedding problems. It is found that embedded microchannels and printing fins within chips can significantly enhance cooling efficiency and temperature uniformity (**Figure** **23**a). I‐type microchannel patterns have been considered in several commercially available CPU‐integrated cooling devices due to their symmetrical flow distribution and vertical inlet/outlet, decreasing higher flow maldistribution in microchannels (Figure 23b).^[^ ^531^ ^]^ Erp et al.^[^ ^4^ ^]^ presented co‐designing microfluidics and GaN‐on‐Si integrated chip for efficient cooling, which realized heat dissipation at ultra‐high heat flux densities (>1700 Wcm^−2^) at the expense of low pumping power (0.57 Wcm^−2^) as shown in Figure 23c. Kwon et al.^[^ ^532^ ^]^ demonstrated a 25‐µm‐thick copper inverse opal porous wicking combined with a microchannel manifold for high‐heat‐flux cooling. A high CHF (485 Wcm^−2^) was achieved by enhanced wicking and bubble minimization caused by microcavities. Xin et al.^[^ ^6^ ^]^ designed and manufactured a miniature loop heat pipe featuring micropillar arrays (15–25 µm) for the thermal management of 3D‐ICs (Figure 23d). The micropillar array wick provided a powerful capillary force, a larger heat exchange area in a limited space, and a stable two‐phase distribution during the high heat flux operation (119 Wcm^−2^). Recently, Tang et al.^[^ ^131^ ^]^ proposed an integrated thermal management strategy for high‐power‐density SiC power modules with die‐level heat flux over 1000 Wcm^−^^2^. Tapered Z‐type manifold microchannels (TZMMCs) (Figure 23e) and connected Z‐type manifold microchannels (CZMMCs) were optimized to improve the overall thermal‐hydraulic performance, resulting in a low junction‐to‐fluid thermal resistance of 9.85 mm^2^·KW^−1^ at a flow rate of 2.16 Lmin^−1^, ensuring a maximum junction temperature less than 127 °C. The integrated cooling approach can support a current density of over 300 A/cm^2^, which is 138% higher than that of conventional cooling packages. On the other hand, the structural optimization of heat sinks using topological methods has been an attractive approach, with early studies dating back to Chen et al.^[^ ^258^ ^]^ and Yu et al.^[^ ^533^ ^]^ Incorporating novel technologies or theoretical approaches facilitates the structural optimization of heat sinks and their cooling performance at micro/nanoscales. Recently, Luo et al.^[^ ^428^ ^]^ optimized lightweight heat sinks for LEDs cooling using amorphous network structures via a five‐step process based on feature analysis, which achieved around 50% weight reduction while maintaining cooling efficiency. Experimental validation with 3D‐printed AlSi_10_Mg heat sinks with various flow channel arrangements showed network designs matched or exceeded the performance of bulk heat sinks. Furthermore, ML methods were employed in predicting the thermal transport properties of networks with complex geometries^[^ ^449^ ^]^ and amorphous materials,^[^ ^534^ ^]^ providing a theoretical basis for the performance evaluation of complex‐structured heat sinks and thermophysical properties of micro/nanostructured materials used in electronic devices.

**Figure 24:**
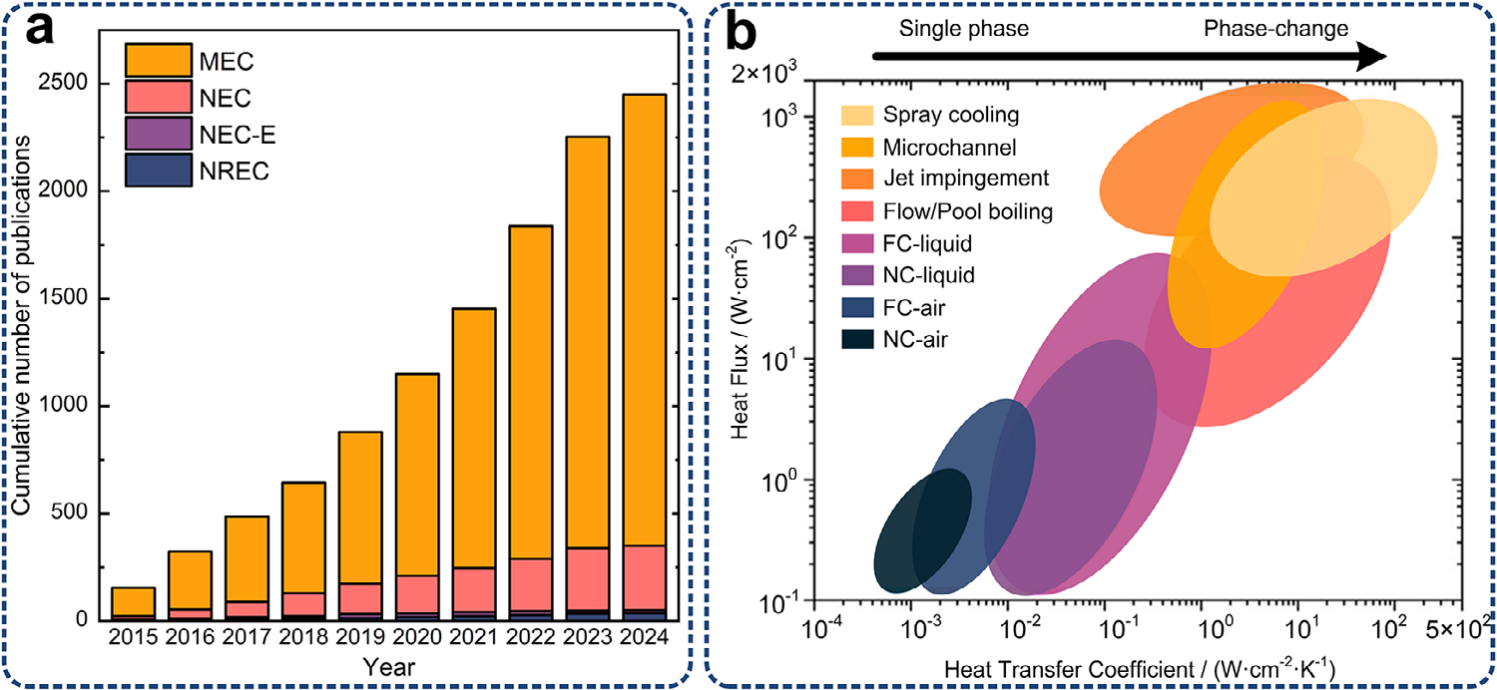
a) The annual publication count for various electronic cooling technologies. MEC, NEC, NEC‐E, and NREC represent microstructure‐based electronic cooling, nanostructure‐based electronic cooling, experimental studies on microstructure‐based electronic cooling, and nonreciprocal‐based electronic cooling. b) Heat transfer performance of different cooling methods, FC and NC represent single‐/two‐phase forced convection and natural convection, respectively. Reproduced with permission.^[^ ^5^ ^]^ Copyright 2024, Elsevier.

Micro/nanostructures demonstrate significant potential in electronic thermal management, while the practice of micro/nanotechnology‐based thermal management in electronics remains in its early stages. As shown in the literature statistics for the last decade, experimental studies on nanostructure‐based electronic cooling are scarcely involved (**Figure** **24**a). For TIMs, liquid metal composites exhibit excellent thermal conductivity and cycling stability, but challenges persist in understanding interfacial mechanisms, ensuring material sustainability, and balancing mechanical reliability. For heat sinks, although micro/nanostructural optimization can support high‐heat‐flux scenarios, issues such as flow maldistribution, fabrication complexity, and the trade‐off between lightweight design and mechanical strength hinder broader application. Future efforts should focus on advancing material design, structural optimization, and reliability assurance. Consequently, there is substantial potential for future advancements, particularly in applying nonreciprocal heat conduction, thermal radiation, and novel modes of latent heat dissipation at micro/nanoscales to electronic cooling systems since the heat flux density of phase‐changing technologies exceeds that of single‐phase cases by several orders of magnitude (Figure 24b).

## 9 Conclusion 

The rapid advancement in 3D‐ICs and AI hardware, marked by miniaturization and increasing power densities, has highlighted the critical need for efficient thermal management systems. This review underscores the pivotal role of micro/nanostructures in addressing heat dissipation challenges at the chip, board, and system levels. These structures leverage their unique material properties and geometries to enhance heat transfer via conduction, convection, phase transition and radiation, particularly in facilitating targeted thermal management at each level rather than taking measures after the heat spreads throughout, as conventional cooling methods. Micro/nanostructures have revolutionized heat conduction pathways, ensuring efficient thermal transport in integrated electronic systems. Metamaterials with anisotropic thermal conductivity, thermal rectifiers, and innovative bonding technologies enhance conduction by reducing thermal resistance and tailoring heat flow pathways. Specific structures, such as graphene nanoribbons, carbon nanotubes, and heterostructures, demonstrate exceptional thermal properties, allowing for targeted heat management and improved reliability. In convection, bio‐inspired structures, optimized microchannels, and surface wettability engineering improve flow patterns and heat transfer efficiency. Especially, fluid metamaterials based on micro/nanostructures enable multifunctional flow manipulation, such as cloaking, concentration, and rotation. Phase transition enhancement can be achieved via efficient melting, nucleation site optimization, and capillary‐driven liquid replenishment, which highlights the significant role of adaptive reconstruction of liquid transport paths in local hot spots. Enhanced radiation heat transfer by integrating advanced nanostructures and coatings has gained traction due to its unique strengths in efficient heat dissipation and scalability for miniaturized systems compared to conduction or convection. Inspired by natural systems, bio‐inspired materials such as butterfly wing scales and silk cocoon fibers demonstrated the potential for structural color and high emissivity, offering multifunctional cooling solutions. Engineered structures at micro/nanoscales can achieve desired optical properties with specific geometric parameters. Besides, NTR devices have been theoretically validated to radiate infrared emission in designing directions for highly efficient cooling, which, however, is currently restricted due to the application of an external magnetic field. Advanced design and manufacturing methods have facilitated the precise fabrication of these structures. Among them, structure‐performance inverse optimization strategies combined with machine learning are promising to accelerate the design process of thermal management systems. Practical applications in thermal interface materials, heat sinks, and cooling systems demonstrate their potential, though challenges remain in scalability, durability, and integration.

## 10 Outlook

The examination of the thermal management requirements of multi‐scale electronic devices, in combination with application cases of micro/nanostructures for efficient heat dissipation, highlights several key prospects:

1. Previous research and applications in on‐chip thermal management remain insufficient. Effective fluid management through hydrodynamic metamaterials is crucial for mitigating system failures caused by clogging, fouling, or coolant evaporation. AI‐driven optimization of microfluidic systems, leveraging nanotechnology and bio‐inspired phase‐change mechanisms (e.g., the sweat evaporation process in living organisms), presents a viable strategy for controllable evaporation and spray cooling microsystems.
2. LMPCMs, particularly Ga‐based systems, hold great promise for use in TIMs due to their superior heat transfer capacity, while challenges such as density, cost, corrosion, and leakage necessitate innovations in composite design, micro/nano‐encapsulation, and paste‐like formulations. Nevertheless, given the significant impact of size on the melting characteristics of PCMs, their performance in nano‐scale confinement (nanocapsules) requires further in‐depth investigation.
3. Future advancements in electronic cooling via evaporation rely on multiscale structural coordination, adaptive gas‐liquid management, and intelligent inverse design. Synergistic micro/nanostructured designs are expected to regulate bubble dynamics and liquid transport simultaneously, while adaptive gas–liquid management enabled by smart‐responsive or dynamically tunable wettability materials will allow reconstruction of liquid pathways in high‐heat‐flux targets.
4. Nonreciprocal heat transfer, facilitating directional radiative dissipation, offers an efficient means of transferring excess heat to a pre‐designated cold source. Unlike convection and conduction, this approach circumvents inherent limitations, potentially reducing system complexity and enhancing energy efficiency. In situ fabrication of polarization‐independent metamaterials on heating elements at the board and system levels (e.g., DC) holds opportunities for achieving rapid thermal dissipation via radiation.
5. Combining non‐reciprocal heat transfer with emerging active thermal management systems, particularly micro/nanostructure‐based designs of all‐solid elastocaloric^[^ ^535^, ^536^, ^537^ ^]^ and electrocaloric cooling systems,^[^ ^538^, ^539^, ^540^, ^541^, ^542^ ^]^ holds promise for next‐generation electronic thermal management technologies. A typical example is the integration of non‐reciprocal thermal conductive metamaterials with an elastocaloric cooler, demonstrating a good cooling performance and fatigue resistance over two million cycles.^[^ ^537^ ^]^ The new system eliminated the need for periodic contact switching between components due to heat flow manipulation, enabling continuous heat flow control and buckling‐free, uniform compression. It provided a new design paradigm for compact, efficient, and long‐life solid‐state cooling systems that address the limitations in existing devices. However, the coupling of electrocaloric cooling with the thermal manipulation technique remains unexplored. Introducing micro/nanostructure‐based non‐reciprocal heat transfer, in conjunction with intrinsic material properties, offers a pathway to mitigate or substitute the influence of external field sources.
6. Advancements in manufacturing techniques are crucial for overcoming existing limitations. Improving the cost‐effectiveness and scalability of micro‐ and nanostructured manufacturing is critical for its widespread adoption. Nanoimprint lithography exhibits notable potential among the reviewed approaches due to its high precision and processing efficiency for the fabrication of architectures at the nanoscale.

## Conflict of Interest

The authors declare no conflict of interest.

## Supporting information



Supporting Information

## References

[1] X. Zhang , Z. Ji , J. Wang , X. Lv , Appl. Therm. Eng. 2023, 235, 121294.

[2] W. Chen , X. Shi , J. Zou , Z. Chen , Mater. Sci. Eng.: R: Rep. 2022, 151, 100700.

[3] Y. K. Joshi , S. V. Garimella , Microelectron. J. 2003, 34, 169.

[4] R. van Erp , R. Soleimanzadeh , L. Nela , G. Kampitsis , E. Matioli , Nature 2020, 585, 211. 32908265 10.1038/s41586-020-2666-1

[5] Z. Li , H. Luo , Y. Jiang , H. Liu , L. Xu , K. Cao , H. Wu , P. Gao , H. Liu , Appl. Therm. Eng. 2024, 251, 123612.

[6] D. Zhou , Y. Chen , W. Gao , G. Xin , Int. J. Therm. Sci. 2024, 199, 108906.

[7] V. Venkatadri , B. Sammakia , K. Srihari , D. Santos , J. Electron. Packag. 2011, 133, 041011.

[8] J. L. Smoyer , P. M. Norris , Heat Transfer Eng. 2018, 40, 269.

[9] M. Coutinho , D. Bento , A. Souza , R. Cruz , F. Afonso , F. Lau , A. Suleman , F. R. Barbosa , R. Gandolfi , W. Affonso , F. I. K. Odaguil , M. F. Westin , R. J. N. dos Reis , C. R. I. da Silva , Appl. Therm. Eng. 2023, 227, 120427.

[10] A. B. Awan , Int. J. Thermofluids 2023, 20, 100517.

[11] W. Hua , L. Zhang , X. Zhang , J. Mol. Liq. 2021, 340, 117183.

[12] Z. Lu , W. Huang , M. R. Stan , K. Skadron , J. Lach , IEEE Trans. Very Large Scale Integr. VLSI Syst. 2007, 15, 159.

[13] J. Mathew , S. Krishnan , J. Electron. Packag. 2021, 144.

[14] H. Sanchez , B. Kuttanna , T. Olson , M. Alexander , G. Gerosa , R. Philip , in Proceedings IEEE Compcon 97 , IEEE, Piscataway, NJ 1997, 325.

[15] R. Kandasamy , X.‐Q. Wang , A. S. Mujumdar , Appl. Therm. Eng. 2008, 28, 1047.

[16] S. Sarkar , R. Gupta , T. Roy , R. Ganguly , C. M. Megaridis , Int. J. Heat. Mass. Transfer. 2023, 206, 123888.

[17] D. H. Wang , M. Z. Wang , Y. H. Peng , Y. Zhang , Appl. Therm. Eng. 2022, 205, 117860.

[18] L. Micheli , N. Sarmah , X. Luo , K. S. Reddy , T. K. Mallick , Renewable Sustainable Energy Rev. 2013, 20, 595.

[19] J. Siriwardana , S. Jayasekara , S. K. Halgamuge , Appl. Energy 2013, 104, 207.

[20] R. Curtis , T. Shedd , E. B. Clark , Performance comparison of five data center server thermal management technologies, Dell Technologies, Round Rock, TX, 2023.

[21] Catherine . NVIDIA HGX B200 and thoughts on its liquid cooling solution , https://www.fibermall.com/blog/nvidia‐hgx‐b200‐cooling‐solution.htm?srsltid=AfmBOooIif2_tgZ82JGA2S_a87AQzoG4Fy6ArAPPUzUJjJijBlDNMuKj, 2024.

[22] A. Chuttar , D. Banerjee , Electronics 2021, 10, 2785.

[23] Z. Haddad , D. Belkadi , A. Mourad , A. Aissa , Z. Said , O. Younis , A. Alazzam , E. Abu‐Nada , J. Power Sources 2024, 603, 234382.

[24] Z.‐Q. Yu , M.‐T. Li , B.‐Y. Cao , Int. J. Extreme Manuf. 2024, 6, 022005.

[25] M. F. Abdullah , M. R. Mat Hussin , M. A. Ismail , S. K. Wan Sabli , Microelectron. Eng. 2023, 273, 111958.

[26] F. Tavakkoli , S. Ebrahimi , S. Wang , K. Vafai , Int. J. Heat. Mass. Transfer. 2016, 97, 337.

[27] X. Zhu , C. Chen , J. Zhang , G. Xin , J. Refrig. 2023, 44, 15.

[28] Y. Çengel , A. Ghajar , Heat and mass transfer: fundamentals & applications, McGraw‐Hill, New York, 2011.

[29] J. Zhang , X. Yang , Y. Feng , Y. Li , M. Wang , J. Shen , L. Wei , D. Liu , S. Wu , Z. Cai , F. Xu , X. Wang , W. Ge , B. Shen , Phys. Rev. Mater.. 2020, 4, 073402.

[30] T.‐L. Luo , Y.‐F. Ding , B.‐J. Wei , J.‐Y. Du , X.‐Y. Shen , G.‐M. Zhu , B.‐W. Li , Acta Phys. Sin. 2023, 72, 234401.

[31] E. Lieb , J. L. Lebowitz , Z. Rieder , J. Math. Phys. 1967, 8, 1073.

[32] B. Li , J. Wang , Phys. Rev. Lett. 2003, 91, 044301. 12906664 10.1103/PhysRevLett.91.044301

[33] X. Xu , J. Chen , B. Li , J. Phys.: Condens. Matter 2016, 28, 483001. 27665943 10.1088/0953-8984/28/48/483001

[34] Y. Yu , T. Minhaj , L. Huang , Y. Yu , L. Cao , Phys. Rev. Appl. 2020, 13, 034059.

[35] X. Xu , L. F. C. Pereira , Y. Wang , J. Wu , K. Zhang , X. Zhao , S. Bae , C. T. Bui , R. Xie , J. T. L. Thong , B. H. Hong , K. P. Loh , D. Donadio , B. Li , B. Özyilmaz , Nat. Commun. 2014, 5, 3689. 24736666 10.1038/ncomms4689

[36] S. Lepri , R. Livi , A. Politi , Phys. Rev. Lett. 1997, 78, 1896.

[37] S. Maruyama , Phys. B 2002, 323, 193.

[38] C. W. Chang , D. Okawa , H. Garcia , A. Majumdar , A. Zettl , Phys. Rev. Lett. 2008, 101, 075903. 18764555 10.1103/PhysRevLett.101.075903

[39] V. Lee , C. Wu , Z. Lou , W. Lee , C. Chang , Phys. Rev. Lett. 2017, 118, 135901. 28409955 10.1103/PhysRevLett.118.135901

[40] F. Yao , S. Xia , H. Wei , J. Zheng , Z. Yuan , Y. Wang , B. Huang , D. Li , H. Lu , D. Xu , Nano Lett. 2022, 22, 6888. 36054095 10.1021/acs.nanolett.2c01050

[41] H. Asegun , C. Gang , Phys. Rev. Lett. 2008, 101, 235502.19113566

[42] N. Yang , X. Xu , G. Zhang , B. Li , AIP Adv. 2012, 2, 041410.

[43] J. Chen , X. Xu , J. Zhou , B. Li , Rev. Modern Phys. 2022, 94, 025002.

[44] B. Hu , B. Li , H. Zhao , Phys. Rev. E 1998, 57, 2992.

[45] G. Zhang , B. Li , J. Chem. Phys. 2005, 123, 114714. 16392590 10.1063/1.2036967

[46] N. Yang , G. Zhang , B. Li , Nano Today 2010, 5, 85.

[47] J. Liu , R. Yang , Phys. Rev. B 2012, 86, 104307.

[48] L. Yang , Y. Tao , Y. Zhu , M. Akter , K. Wang , Z. Pan , Y. Zhao , Q. Zhang , Y.‐Q. Xu , R. Chen , T. T. Xu , Y. Chen , Z. Mao , D. Li , Nat. Nanotechnol. 2021, 16, 764. 33859389 10.1038/s41565-021-00884-6

[49] S. Liu , P. Hanggi , N. Li , J. Ren , B. Li , Phys. Rev. Lett. 2014, 112, 040601. 24580429 10.1103/PhysRevLett.112.040601

[50] S.‐N. Li , B.‐Y. Cao , Appl. Math. Lett 2020, 99, 105992.

[51] D. G. Cahill , W. K. Ford , K. E. Goodson , J. Appl. Phys. 2003, 93, 793.

[52] D. G. Cahill , P. V. Braun , G. Chen , D. R. Clarke , S. Fan , K. E. Goodson , P. Keblinski , W. P. King , G. D. Mahan , A. Majumdar , H. J. Maris , S. R. Phillpot , E. Pop , L. Shi , Appl. Phys. Rev. 2014, 1, 011305.

[53] Z. M. Zhang , Nano/microscale heat transfer, McGraw‐Hill, New York 2007, p. 480.

[54] S. Volz , Microscale and nanoscale heat transfer, Springer, Berlin, Heidelberg 2007.

[55] C. B. Sobhan , G. P. Peterson , Microscale and Nanoscale Heat Transfer: Fundamentals and Engineering Applications, CRC press, Boca Raton 2008.

[56] N. Mingo , Phys. Rev. B:Condens. Matter Mater. Phys. 2003, 68, 113308.

[57] G. Chen , Nanoscale Energy Transport and Conversion: A Parallel Treatment of Electrons, Molecules, Phonons, and Photon, Oxford University Press, New York, NY, USA 2005.

[58] J. M. Ziman , Electrons and Phonons: The Theory of Transport Phenomena in Solids, Oxford University Press, New York, NY, USA 1960.

[59] P. G. Klemens , Solid State Physics, Academic Press, New York, USA 1958, p. 99.

[60] H. J. Maris , S.‐i. Tamura , Phys. Rev. B 2012, 85, 054304.

[61] K. Termentzidis , P. Chantrenne , P. Keblinski , Phys. Rev. B 2009, 79, 214307.

[62] L. C. Burmeister , Convective heat transfer, John Wiley & Sons, New York 1993.

[63] K. Wark , Thermodynamics, 5th ed., McGraw‐Hill, New York, USA, 1988.

[64] E. V. Pavlyuk , Eur. Phys. J. 2024, 233, 3321.

[65] A. M. Meirmanov , The Stefan Problem, De Gruyter, Berlin, Germany 1992.

[66] J. Zheng , X. Li , W. Xing , B. Fu , C. Song , W. Shang , P. Tao , T. Deng , Appl. Therm. Eng. 2022, 213, 118766.

[67] E. S. Asmolov , M. N. Kogan , Fluid Dyn. 1984, 19, 107.

[68] T. Yang , H. Lin , K.‐T. Lin , D. M. Saldarriaga , G. Yang , C. Guo , H. Zhang , J. Zhang , S. Fraser , A. K.‐T. Lau , T. Ma , B. Jia , Carbon 2022, 199, 469.

[69] N. Amrofel , M. Dymitrowska , A. Obliger , A.‐J. Tinet , F. Golfier , Phys. Fluids 2024, 36, 022028.

[70] K. K. Nanda , F. E. Kruis , H. Fissan , Phys. Rev. Lett. 2002, 89, 256103. 12484904 10.1103/PhysRevLett.89.256103

[71] Z. Tian , L. Fei , C. Wang , K. Guo , W. Tian , S. Qiu , G. Su , D. Derome , J. Carmeliet , Phys. Fluids 2025, 37, 033351.

[72] Z. Lu , I. Kinefuchi , K. L. Wilke , G. Vaartstra , E. N. Wang , Nat. Commun. 2019, 10, 1. 31147534 10.1038/s41467-019-10209-w PMC6542818

[73] J. E. Vesper , C. S. Obiji , R. Westerwaal , C. Boelsma , S. Kenjereš , C. R. Kleijn , Appl. Therm. Eng. 2021, 195C, 117099.

[74] M. Jakob , W. Linke , Physikalische Zeitschrift 1935, 36, 267.

[75] D. E. Kim , D. I. Yu , D. W. Jerng , M. H. Kim , H. S. Ahn , Exp. Thermal Fluid Sci. 2015, 66, 173.

[76] H. Chu , N. Xu , X. Yu , H. Jiang , W. Ma , F. Qiao , Appl. Therm. Eng. 2022, 215, 119041.

[77] P. R. Sharma , in 11th International Heat Transfer Conference , Kyongju, South Korea, 1998.

[78] L.‐H. Chien , R. L. Webb , Int. J. Heat. Mass. Transfer. 1998, 41, 2183.

[79] N. I. Kolev , Exp. Thermal Fluid Sci. 1994, 8, 167.

[80] L. Z. Zeng , J. F. Klausner , R. Mei , Int. J. Heat. Mass. Transfer. 1993, 36, 2261.

[81] C. Ramaswamy , Y. Johsi , W. Nakayama , W. B. Johnson , Int. J. Heat. Mass. Transfer. 2002, 45, 4761.

[82] C. Ramaswamy , Y. Joshi , W. Nakayama , W. B. Johnson , Int. J. Heat. Mass. Transfer. 2003, 46, 4257.

[83] W. B. Zhou , Y. J. Luan , X. L. Dai , X. G. Hu , Int. J. Therm. Sci. 2019, 135, 434.

[84] H. Moghadasi , H. Saffari , Int. J. Mech. Sci. 2021, 196, 106270.

[85] L. Zhang , J. Xu , G. Liu , J. Lei , Int. J. Therm. Sci. 2020, 152, 106325.

[86] X. Quan , D. Wang , P. Cheng , Int. J. Heat. Mass. Transfer. 2017, 108, 32.

[87] L. Zhang , T. Wang , S. Kim , Y. Jiang , Int. J. Heat. Mass. Transfer. 2020, 146, 118820.

[88] K.‐H. Chu , Y. Soo Joung , R. Enright , C. R. Buie , E. N. Wang , Appl. Phys. Lett. 2013, 102, 151602.

[89] T. Foulkes , J. Oh , R. Pilawa‐Podgurski , N. Miljkovic , Int. J. Heat. Mass. Transfer. 2019, 133, 1154.

[90] M. F. Modest , Radiative Heat Transfer, 3rd ed., Academic Press, Amsterdam 2003.

[91] X. Yin , R. Yang , G. Tan , S. Fan , Science 2020, 370, 786. 33184205 10.1126/science.abb0971

[92] X. Song , H. Gong , H. Li , M. Zhang , L. Jiang , C. Wang , P. Jiang , H. Wang , K. Cao , G. Liu , Q. Zhao , T. Fan , Adv. Funct. Mater. 2024, 35, 2413191.

[93] K.‐T. Lin , J. Han , K. Li , C. Guo , H. Lin , B. Jia , Nano Energy 80, 2021: 105517.

[94] M. M. Hossain , M. Gu , Adv. Sci. 2016, 3, 1500360. 10.1002/advs.201500360 PMC5067572 27812478

[95] K. J. Shayegan , S. Biswas , B. Zhao , S. Fan , H. A. Atwater , Nat. Photonics 17, 2023: 891.

[96] P. M. Robitaille , Prog. Phys. 2009, 2, 3.

[97] Z. Chen , S. Yu , C. Yuan , X. Luo , R. Hu , Int. J. Heat. Mass. Transfer. 2024, 222, 125202.

[98] Y. Park , Z. Omair , S. Fan , ACS Photonics 2022, 9, 3943.

[99] N. Nikita , C. Michele , A. Andrea , Nanophotonics 2023, 12, 589.39635396

[100] S. Yang , M. Liu , C. Zhao , S. Fan , C.‐W. Qiu , Nat. Photonics 2024, 18, 412.

[101] Z. Zhang , L. Zhu , Phys. Rev. Appl. 2022, 18, 027001.

[102] Y. Li , J. Li , M. Qi , C.‐W. Qiu , H. Chen , Phys. Rev. B 2021, 103, 014307.

[103] J. Yang , M. Liu , T. Wang , G. Meng , Z. Wang , C. Guo , K. T. Lin , H. Lin , B. Jia , Small 2024, 20, 2402575. 10.1002/smll.202402575 38860359

[104] J. W. Palko , H. Lee , C. Zhang , T. J. Dusseault , T. Maitra , Y. Won , D. D. Agonafer , J. Moss , F. Houshmand , G. Rong , J. D. Wilbur , D. Rockosi , I. Mykyta , D. Resler , D. Altman , M. Asheghi , J. G. Santiago , K. E. Goodson , Adv. Funct. Mater. 2017, 27, 1703265.

[105] Z. Cheng , Z. Huang , J. Sun , J. Wang , T. Feng , K. Ohnishi , J. Liang , H. Amano , R. Huang , Appl. Phys. Rev. 2024, 11, 041324.

[106] W. Delmas , A. Jarzembski , M. Bahr , A. McDonald , W. Hodges , P. Lu , J. Deitz , E. Ziade , Z. T. Piontkowski , L. Yates , ACS Appl. Mater. Interfaces 2024, 16, 11003. 38373710 10.1021/acsami.3c17778

[107] O. Moutanabbir , Y. J. Chabal , M. Chicoine , S. Christiansen , R. Krause‐Rehberg , F. Schiettekatte , R. Scholz , O. Seitz , S. Senz , F. Süßkraut , U. Gösele , Nucl. Instrum. Methods Phys. Res., Sect. B 2009, 267, 1264.

[108] F. Mu , B. Xu , X. Wang , R. Gao , S. Huang , K. Wei , K. Takeuchi , X. Chen , H. Yin , D. Wang , J. Yu , T. Suga , J. Shiomi , X. Liu , J. Alloys Compd. 2022, 905, 164076.

[109] T. Matsumae , Y. Kurashima , H. Takagi , Y. Shirayanagi , S. Hiza , K. Nishimura , E. Higurashi , Scr. Mater. 2022, 215, 114725.

[110] A. Kobayashi , H. Tomiyama , Y. Ohno , Y. Shimizu , Y. Nagai , N. Shigekawa , J. Liang , Funct. Diamond 2022, 2, 142.

[111] H. Takagi , K. Kikuchi , R. Maeda , T. R. Chung , T. Suga , Appl. Phys. Lett. 1996, 68, 2222.

[112] G. Ma , X. Xiao , B. Meng , Y. Ma , X. Xing , X. Wang , F. Mu , C. Yuan , ACS Appl. Mater. Interfaces 2024, 16, 20826. 38598525 10.1021/acsami.4c02161

[113] W. Xu , T. You , Y. Wang , Z. Shen , K. Liu , L. Zhang , Sun , R. Qian , Z. An , F. Mu , T. Suga , G. Han , X. Ou , Y. Hao , X. Wang , Fundam. Res. 2021, 1, 691.

[114] H. Shi , K. Huang , F. Mu , T. You , Q. Ren , J. Lin , W. Xu , T. Jin , H. Huang , A. Yi , S. Zhang , Z. Li , M. Zhou , J. Wang , K. Xu , X. Ou , Semicond. Sci. Technol. 2020, 35, 125004.

[115] W. Xu , T. Zhao , L. Zhang , K. Liu , H. Sun , Z. Qu , T. You , A. Yi , K. Huang , G. Han , F. Mu , T. Suga , X. Ou , Y. Hao , ACS Appl. Electron. Mater. 2024, 6, 1710.

[116] Z. Shen , W. Xu , Y. Chen , J. Lin , Y. Xie , K. Huang , T. You , G. Han , X. Ou , Sci. China Mater. 2022, 66, 756.

[117] T. Matsumae , S. Okita , S. Fukumoto , M. Hayase , Y. Kurashima , H. Takagi , ACS Appl. Nano Mater. 2023, 6, 14076.

[118] T. Matsumae , Y. Kurashima , H. Takagi , H. Umezawa , E. Higurashi , J. Appl. Phys. 2021, 130, 085303.

[119] T. Matsumae , Y. Kurashima , H. Umezawa , K. Tanaka , T. Ito , H. Watanabe , H. Takagi , Appl. Phys. Lett. 2020, 116, 141602.

[120] T. Nieminen , T. Koskinen , V. Kornienko , G. Ross , M. Paulasto‐Kröckel , ACS Appl. Electron. Mater. 2024, 6, 2413. 38680727 10.1021/acsaelm.4c00068 PMC11044587

[121] S. Fukumoto , T. Matsumae , Y. Kurashima , H. Takagi , H. Umezawa , M. Hayase , E. Higurashi , Appl. Phys. Lett. 2020, 117, 201601.

[122] F. Shoya , M. Takashi , K. Yuichi , T. Hideki , U. Hamao , H. Masanori , H. Eiji , in 2021 International Conference on Electronics Packaging (ICEP 2021) , Tokyo, Japan, 2021.

[123] Y. Song , D. Shoemaker , J. H. Leach , C. McGray , H.‐L. Huang , A. Bhattacharyya , Y. Zhang , C. U. Gonzalez‐Valle , T. Hess , S. Zhukovsky , K. Ferri , R. M. Lavelle , C. Perez , D. W. Snyder , J.‐P. Maria , B. Ramos‐Alvarado , X. Wang , S. Krishnamoorthy , J. Hwang , B. M. Foley , S. Choi , ACS Appl. Mater. Interfaces 2021, 13, 40817. 34470105 10.1021/acsami.1c09736

[124] T. Matsumae , Y. Kurashima , H. Takagi , H. Umezawa , E. Higurashi , Scr. Mater. 2021, 191, 52.

[125] D. B. Ingerly , S. Amin , L. Aryasomayajula , A. Balankutty , D. Borst , A. Chandra , K. Cheemalapati , C. S. Cook , R. Criss , K. Enamul , W. Gomes , D. Jones , K. C. Kolluru , A. Kandas , G.‐S. Kim , H. Ma , D. Pantuso , C. F. Petersburg , M. Phen‐givoni , A. M. Pillai , A. Sairam , P. Shekhar , P. Sinha , P. Stover , A. Telang , Z. Zell , in 2019 IEEE International Electron Devices Meeting (IEDM) , San Francisco, CA, 2019.

[126] OpenAI. , GPT‐4o System Card, (accessed: February 2024);

[127] Product Brief. Intel. (accessed: December 2023).

[128] Intel^®^ Core™ i9‐13900K Processor (accessed: August 2024).

[129] H. Huang , C. Wang , T. Wu , Z. Wu , J. Zheng , Surf. Interf. 2024, 55, 105406.

[130] C. Liu , J. Yang , Y. Li , J. Fu , W. Yu , H. Xie , Surf. Interf. 2024, 47, 104204.

[131] W. Tang , X. Huang , Z. Chen , K. Sheng , Z. Wu , IEEE J. Emerging Sel. Top. Power Electron. 2024, 13, 3524214.

[132] N. Yu , J. Ballato , M. Digonnet , P. D. Dragic , Curr. Opt. Photonics 2022, 6, 521.

[133] D. Lu , Y. Ye , R. Liu , M. Wu , X. Du , L. Yu , J. Qiao , Z. Liu , Y. Kong , B. Jiao , X. Ma , Y. Hao , IEEE Trans. Electron Devices 2024, 71, 502.

[134] S. C. Maroo , A. Zou , M. Gupta , Passive nano‐heat pipes for cooling and thermal management of electronics and power conversion devices, Syracuse University, Syracuse, NY 2018.

[135] Y. Kim , Thermal management function featured enclosure for power conversion system, has blocking film formed to be inclined at angle in direction to prevent water or dust along with external air, blower that includes centrifugal blower, Datsu Energy Co Ltd(Dats‐N), Seoul, South Korea 2022.

[136] A. Habibi Khalaj , S. K. Halgamuge , Appl. Energy 2017, 205, 1165.

[137] J. Siriwardana , S. K. Halgamuge , T. Scherer , W. Schott , Energy Build. 2012, 50, 81.

[138] R. R. Schmidt , E. E. Cruz , M. Iyengar , IBM J. Res. Dev. 2005, 49, 709.

[139] W. Feng , K. Kikuchi , M. Hidaka , H. Yamamori , Y. Araga , K. Makise , S. Kawabata , Appl. Phys. Lett. 2021, 118, 174004.

[140] M. J. Martin , C. Hughes , G. Moreno , E. B. Jones , D. Sickinger , S. Narumanchi , R. Grout , IEEE Trans. Sustain. Comput. 2022, 7, 864.

[141] D. Q. Adams , et al., Prog.Part. Nucl. Phys. 2022, 122, 103902.

[142] T. Poole , T. Marsh , A. J. Matthews , Cryogenics 2022, 126, 103538.

[143] S. Krinner , S. Storz , P. Kurpiers , P. Magnard , J. Heinsoo , R. Keller , J. Lütolf , C. Eichler , A. Wallraff , EPJ Quantum Technol. 2019, 6, 2.

[144] A. L. Moore , L. Shi , Mater. Today 2014, 17, 163.

[145] Y. Fan , H. Chen , X. Liu , Y. Zhao , Y. Huang , J. Liu , C. Wang , Adv. Mater. 2025, 14, 06795. 10.1002/adma.202506795 40827823

[146] J. B. Pendry , D. Schurig , D. R. Smith , Science 2006, 312, 1780. 16728597 10.1126/science.1125907

[147] B. Li , L. Wang , G. Casati , Phys. Rev. Lett. 2004, 93, 184301. 15525165 10.1103/PhysRevLett.93.184301

[148] S. Sklan , B. Li , Sci. Rep. 2018, 8, 4436. 29535315 10.1038/s41598-018-22215-x PMC5849733

[149] X. Bai , Q. Hu , X. Zeng , R. Sun , J. Xu , in 2021 22nd International Conference on Electronic Packaging Technology (ICEPT) , Xianmen, China, 2021, pp. 1–4.

[150] C. Z. Fan , Y. Gao , J. P. Huang , Appl. Phys. Lett. 2008, 92, 251907.

[151] Y. Li , W. Li , T. Han , X. Zheng , J. Li , B. Li , S. Fan , C.‐W. Qiu , Nat. Rev. Mater. 2021, 6, 488.

[152] J.‐H. You , K. Park , Addit. Manuf. 2021, 41, 101947.

[153] Y. Li , A. Wei , D. Datta , Carbon 2017, 113, 274.

[154] M. Shavikloo , S. Kimiagar , Comput. Mater. Sci. 2017, 139, 330.

[155] J. Li , Y. Li , P.‐C. Cao , T. Yang , X.‐F. Zhu , W. Wang , C.‐W. Qiu , Adv. Mater. 2020, 32, 2003823. 10.1002/adma.202003823 32902007

[156] U. Leonhardt , Nature 2013, 498, 440. 23803838 10.1038/498440a

[157] T. Han , X. Bai , D. Gao , J. T. L. Thong , B. Li , C. Qiu , Phys. Rev. Lett. 2014, 112, 054302. 24580600 10.1103/PhysRevLett.112.054302

[158] H. Xu , X. Shi , F. Gao , H. Sun , B. Zhang , Phys. Rev. Lett. 2014, 112, 054301. 24580599 10.1103/PhysRevLett.112.054301

[159] L. Xu , S. Yang , J. Huang , Phys. Rev. Appl. 2019, 11, 054071.

[160] L. Xu , J. Liu , P. Jin , G. Xu , J. Li , X. Ouyang , Y. Li , C.‐W. Qiu , J. Huang , Nat. Sci. Rev. 2023, 10, nwac159. 10.1093/nsr/nwac159 PMC10016200 36935932

[161] E. R. Russell , R. C. Assier , W. J. Parnell , Appl. Math. Modell. 2022, 106, 225.

[162] S. Yang , Y. Zhang , Z. Sha , Z. Huang , H. Wang , F. Wang , J. Li , ACS Appl. Mater. Interfaces 2022, 14, 39354. 35984869 10.1021/acsami.2c09602

[163] L. J. Xu , J. P. Huang , EPL (Europhys. Lett.) 2021, 134, 60001.

[164] L. Xu , G. Xu , J. Huang , C. Qiu , Phys. Rev. Lett. 2022, 128, 145901. 35476493 10.1103/PhysRevLett.128.145901

[165] J. Wang , B. Li , Z. Yao , G. Liu , Phys. Rev. B 2005, 71, 5417.

[166] G. Zhang , B. Li , N. Yang , Appl. Phys. Lett. 2008, 93, 243111.

[167] J. Chen , G. Zhang , B. Li , Appl. Phys. Lett. 2009, 95, 073117.

[168] G. Zhang , B. Li , N. Yang , Appl. Phys. Lett. 2009, 95, 033107.

[169] N. Yang , B. Li , N. Li , L. Wang , Phys. Rev. B 2007, 76, 301.

[170] B. Li , G. Wu , Phys. Rev. B 2007, 76, 5424.

[171] Q. Liang , Y. Wei , Physica B 2014, 437, 36.

[172] S. Hu , M. An , N. Yang , B. Li , Small 2016, 13, 1602726.

[173] H. Wang , S. Hu , K. Takahashi , X. Zhang , H. Takamatsu , J. Chen , Nat. Commun. 2017, 8, 15843. 28607493 10.1038/ncomms15843 PMC5474737

[174] L. Kiani , J. Hasanzadeh , F. Yousefi , P. A. Anaraki , Phys. E 2021, 131, 114724.

[175] L. B. Shi , Y. Y. Zhang , X. M. Xiu , H. K. Dong , Carbon 2018, 134, 103.

[176] X.‐K. Chen , Z.‐X. Xie , Y. Zhang , Y.‐X. Deng , T.‐H. Zou , J. Liu , K.‐Q. Chen , Carbon 2019, 148, 532.

[177] B. Yang , D. Li , L. Qi , T. Li , P. Yang , Phys. Rev. A 2019, 383, 1306.

[178] M. Chen , J. Zhang , X. Shen , G. Zhu , B. Li , Device 2024, 2, 100500.

[179] G. Xu , K. Dong , Y. Li , H. Li , K. Liu , L. Li , J. Wu , C. W. Qiu , Nat. Commun. 2020, 11, 6028. 33247120 10.1038/s41467-020-19909-0 PMC7699644

[180] Y. Liu , X. Cao , J. Li , Y. Zhou , Z. Tong , Z. Zhang , Z. Zhang , D. Jiao , Z. Cheng , L. He , Small 2025, 2500469. 10.1002/smtd.202500469 40411866

[181] H. He , W. Peng , H. Le Ferrand , Adv. Mater. 2024, 36, 2307071. 10.1002/adma.202307071 37936342

[182] J. Guo , G. Xu , D. Tian , Z. Qu , C. W. Qiu , Adv. Mater. 2022, 34, 2200329. 10.1002/adma.202201093 35415933

[183] Q. Ji , X. Chen , J. Liang , G. Fang , V. Laude , T. Arepolage , S. Euphrasie , J. A. I. Martinez , S. Guenneau , M. Kadic , Int. J. Heat. Mass. Transfer. 2022, 196, 123149.

[184] T. Chen , C.‐N. Weng , J.‐S. Chen , Appl. Phys. Lett. 2008, 93, 114103.

[185] S. Narayana , Y. Sato , Phys. Rev. Lett. 2012, 108, 214303. 23003263 10.1103/PhysRevLett.108.214303

[186] R. Schittny , M. Kadic , S. Guenneau , M. Wegener , Phys. Rev. Lett. 2013, 110, 195901. 23705719 10.1103/PhysRevLett.110.195901

[187] H. Li , D. Wang , G. Xu , K. Liu , T. Zhang , J. Li , G. Tao , S. Yang , Y. Lu , R. Hu , S. Lin , Y. Li , C.‐W. Qiu , Nat. Commun. 2024, 15, 2169. 38461277 10.1038/s41467-024-46247-2 PMC10924968

[188] X. Shen , C. Jiang , Y. Li , J. Huang , Appl. Phys. Lett. 2016, 109, 201906.

[189] X. Shen , Y. Li , C. Jiang , Y. Ni , J. Huang , Appl. Phys. Lett. 2016, 109, 031907.

[190] A. Hamed , S. Ndao , Int. J. Heat. Mass. Transfer. 2018, 121, 10.

[191] R. Ju , G. Xu , L. Xu , M. Qi , D. Wang , P. C. Cao , R. Xi , Y. Shou , H. Chen , C. W. Qiu , Y. Li , Adv. Mater. 2023, 35, 2209123. 10.1002/adma.202209123 36621882

[192] B. Li , J. Lan , L. Wang , Phys. Rev. Lett. 2005, 95, 104302. 16196932 10.1103/PhysRevLett.95.104302

[193] B. Hu , L. Yang , Y. Zhang , Phys. Rev. Lett. 2006, 97, 124302. 17025972 10.1103/PhysRevLett.97.124302

[194] S. Chen , D. Donadio , G. Benenti , G. Casati , Phys. Rev. E 2018, 97, 030101. 29776123 10.1103/PhysRevE.97.030101

[195] T. J. Alexander , Phys. Rev. E 2020, 101, 062122. 32688508 10.1103/PhysRevE.101.062122

[196] N. Li , J. Ren , L. Wang , G. Zhang , P. Hanggi , B. Li , Rev. Mod. Phys. 2012, 84, 1045.

[197] C. W. Chang , D. Okawa , A. Majumdar , A. Zettl , Science 2006, 314, 1121. 17110571 10.1126/science.1132898

[198] C. W. Chang , D. Okawa , H. Garcia , A. Majumdar , A. Zettl , Phys. Rev. Lett. 2007, 99, 045901. 17678375 10.1103/PhysRevLett.99.045901

[199] D. He , S. Buyukdagli , B. Hu , Phys. Rev. B 2009, 80, 104302.

[200] Z. Shao , L. Yang , H. Chan , B. Hu , Phys. Rev. E 2009, 79, 061119. 10.1103/PhysRevE.79.061119 19658485

[201] L. Wang , B. Li , Phys. Rev. Lett. 2007, 99, 177208. 17995368 10.1103/PhysRevLett.99.177208

[202] A. Hamed , M. Elzouka , S. Ndao , Int. J. Heat. Mass. Transfer. 2019, 134, 359.

[203] L. Wang , B. Li , Phys. Rev. Lett. 2009, 101, 267203. 10.1103/PhysRevLett.101.267203 19437667

[204] G. Marchegiani , A. Braggio , F. Giazotto , Appl. Phys. Lett. 2021, 118, 022602.

[205] D. Goury , R. Sánchez , Appl. Phys. Lett. 2019, 115, 092601.

[206] Y. Z. Liu , Y. Xu , S. Zhang , W. Duan , Phys. Rev. B 2017, 96, 064106.

[207] Y. Wang , X. Guo , X. Yang , J. Dyn. Control. Syst 2024, 22, 37.

[208] X. Fang , J. Wen , L. Cheng , B. Li , Phys. Rev. Appl. 2021, 15, 054022.

[209] Y.‐F. Ding , G.‐M. Zhu , X.‐Y. Shen , X. Bai , B.‐W. Li , Phys. B 2022, 31, 126301.

[210] J. Ordonez‐Miranda , E. S. Younè , K. Joulain , Phys. Rev. E 2017, 95, 022128. 28297864 10.1103/PhysRevE.95.022128

[211] S. Chen , E. Pereira , G. Casati , EPL 2015, 111, 30004.

[212] K. Klinar , M. M. Rojo , Z. Kutnjak , J. Appl. Phys. 2020, 127, 234101.

[213] T. Li , W. Jiang , Y. Zhang , B. Li , L. Wang , D. Niu , H. Liu , L. Yin , Y. Shi , B. Chen , J. Chen , X. Liu , D. Peng , Adv. Funct. Mater. 2022, 32, 2111229.

[214] L. Xu , J. Huang , X. Ouyang , Phys. Rev. E 2021, 103, 032128. 33862724 10.1103/PhysRevE.103.032128

[215] Y. Li , X. Shen , Z. Wu , J. Huang , Y. Chen , Y. Ni , J. Huang , Phys. Rev. Lett. 2015, 115, 195503. 26588397 10.1103/PhysRevLett.115.195503

[216] P. R. Gaddam , S. T. Huxtable , W. A. Ducker , Int. J. Heat. Mass. Transfer. 2017, 106, 741.

[217] I. Kim , M. Kang , S. Kim , Int. J. Thermophys. 2017, 38, 172.

[218] L. Tang , M. Francoeur , Opt. Express 2017, 25, A1043. 29220982 10.1364/OE.25.0A1043

[219] S. Wang , A. L. Cottrill , Y. Kunai , A. R. Toland , P. W. Liu , W. J. Wang , M. S. Strano , Phys. Chem. Chem. Phys. 2017, 19, 13172. 28489089 10.1039/c7cp02445b

[220] R. Xie , C. T. Bui , B. Varghese , Q. Zhang , C. H. Sow , B. Li , J. T. L. Thong , Adv. Funct. Mater. 2011, 21, 1602.

[221] X. Shen , J. Huang , Int. J. Heat. Mass. Transfer. 2014, 78, 1.

[222] H. Song , J. Liu , B. Liu , J. Wu , H. Cheng , F. Kang , Joule 2018, 2, 442.

[223] L. Xu , J. Huang , X. Ouyang , Appl. Phys. Lett. 2021, 118, 221902.

[224] Y. Dong , L. Xiong , D. N. Basov , Nature 2021, 594, 513. 34163054 10.1038/s41586-021-03640-x

[225] W. Zhao , S. Zhao , H. Li , S. Wang , S. Wang , M. I. B. Utama , S. Kahn , Y. Jiang , X. Xiao , S. Yoo , K. Watanabe , T. Taniguchi , A. Zettl , F. Wang , Nature 2021, 594, 517. 34163053 10.1038/s41586-021-03574-4

[226] Y. Li , K.‐J. Zhu , Y.‐G. Peng , W. Li , T. Yang , H.‐X. Xu , H. Chen , X.‐F. Zhu , S. Fan , C.‐W. Qiu , Nat. Mater. 2018, 18, 48. 30510270 10.1038/s41563-018-0239-6

[227] L. Yang , W. Li , J. Lian , H. Zhu , Q. Deng , Y. Zhang , J. Li , X. Yin , L. Wang , Science 2024, 384, 1344. 38900891 10.1126/science.adk4180

[228] S. Feng , P. Zhu , H. Zheng , H. Zhan , C. Chen , J. Li , L. Wang , X. Yao , Y. Liu , Z. Wang , Science 2021, 373, 1344 34529472 10.1126/science.abg7552

[229] H. Chen , J. Cogswell , C. Anagnostopoulos , Lab Chip 2012, 12, 2909. 22699228 10.1039/c2lc20970e

[230] D. Culver , Y. Urzhumov , Phys. Rev. E 2017, 96, 063107. 29347398 10.1103/PhysRevE.96.063107

[231] Y. A. Urzhumov , D. R. Smith , Phys. Rev. E 2012, 86, 056313. 10.1103/PhysRevE.86.056313 23214882

[232] P. T. Bowen , D. R. Smith , Y. A. Urzhumov , Phys. Rev. E 2015, 92, 063030. 10.1103/PhysRevE.92.063030 26764826

[233] J. Park , J. R. Youn , Y. S. Song , Extreme. Mech. Lett. 2021, 42, 101061.

[234] J. Park , J. R. Youn , Y. S. Song , Phys. Rev. Appl. 2019, 12, 061002.

[235] J. W Gaole Dai , Phys. Rev. E 2023, 107, 055108. 37329012 10.1103/PhysRevE.107.055108

[236] S. Balaji , S. Lakshminarayanan , Can. J. Chem. Eng. 2007, 84, 715.

[237] S. Dabiri , M. Hashemi , M. Rahimi , M. Bahiraei , E. Khodabandeh , Energy 2018, 152, 719.

[238] M. Chen , X. Shen , G. Zhu , B. Li , Phys. Fluids 2024, 36, 053611.

[239] P. J , Y. JR , S. YS , Phys. Rev. Lett. 2019, 123, 74502.

[240] M. Chen , X. Shen , L. Xu , Innovation 2022, 3, 100263. 35706453 10.1016/j.xinn.2022.100263 PMC9190057

[241] M. Chen , X. Shen , L. Xu , Droplet 2023, 2, 79.

[242] M. Chen , X. Shen , Z. Chen , J. H. Y. Lo , Y. Liu , X. Xu , Y. Wu , L. Xu , Proc. Natl. Acad. Sci. USA 2022, 119, 9.

[243] O. Boyadjian , E. Boulais , A. T. Gervais , Phys. Rev. Appl. 2022, 17, 014012.

[244] X. Zhou , G. Xu , H. Zhang , Compos. Struct. 2021, 267C, 113866.

[245] L. Sanchis , V. M. García‐Chocano , R. Llopis‐Pontiveros , A. Climente , J. Martínez‐Pastor , F. Cervera , J. Sánchez‐Dehesa , Phys. Rev. Lett. 2013, 110, 124301. 25166808 10.1103/PhysRevLett.110.124301

[246] F. Tay , Y. Zhang , H. Xu , H. Goh , Y. Luo , B. Zhang , Nat. Sci. Rev. 2022, 9, 212. 10.1093/nsr/nwab205 PMC9555300 36248071

[247] W. Li , S. Yang , Y. Chen , C. Li , Z. Wang , Nat. Commun. 2023, 14, 3996. 37414775 10.1038/s41467-023-39289-5 PMC10325955

[248] J. Li , Y. Liu , T. Wu , Z. Xiao , J. Du , H. Liang , C. Zhou , J. Zhou , Nat. Commun. 2024, 15, 5603. 38961073 10.1038/s41467-024-49810-z PMC11222510

[249] S. Wu , S. Sun , J. Ye , L. Wang , Y. Zhang , Adv. Mater. 2025, 2503840. 40320915 10.1002/adma.202503840 PMC12592910

[250] C. Woodcock , X. Yu , J. Plawsky , Y. Peles , Int. J. Heat. Mass. Transfer. 2015, 90, 591.

[251] C. Woodcock , C. Ng'oma , M. Sweet , Y. Wang , Y. Peles , J. Plawsky , Int. J. Heat. Mass. Transfer. 2019, 128, 504.

[252] J. Xu , X. Yu , W. Jin , Int. J. Heat. Mass. Transfer. 2016, 101, 341.

[253] L. X. Zong , G. D. Xia , Y. T. Jia , L. Liu , D. D. Ma , J. Wang , Int. J. Heat. Mass. Transfer. 2020, 146, 118863.

[254] X. Cheng , H. Wu , Int. J. Heat. Mass. Transfer. 2021, 164, 120468.

[255] X. Zhou , C. Zeng , Y. Song , M. Jiao , F. Zhang , M. Liu , Prog. Nucl. Energy 2022, 147, 104190.

[256] W. Duangthongsuk , S. Wongwises , Exp. Thermal Fluid Sci. 2017, 87, 30.

[257] D. Heymann , D. Pence , V. Narayanan , Int. J. Therm. Sci. 2010, 49, 1383.

[258] Y. Chen , P. Cheng , Int. J. Heat. Mass. Transfer. 2002, 45, 2643.

[259] B. Mandelbrot , Science 1967, 156, 636. 17837158 10.1126/science.156.3775.636

[260] K. Horsfield , G. Cumming , Bull. Math. Biophys. 1967, 29, 245. 6051603 10.1007/BF02476898

[261] J. Shi , Z. Chen , M. Shi , Appl. Therm. Eng. 2009, 29, 1792.

[262] K. McCulloh , J. Sperry , F. Adler , Nature 2003, 421, 939. 12607000 10.1038/nature01444

[263] A. Bejan , S. Lorente , Appl. Phys. Rev. 2006, 100.

[264] D. Pence , Microscale Thermophys. Eng. 2003, 6, 319.

[265] H. Tan , L. Wu , M. Wang , Z. Yang , P. Du , Int. J. Heat. Mass. Transfer. 2019, 129, 681.

[266] L. Ghodoossi , Energy Convers. Manage. 2005, 46, 771.

[267] Y. Fan , Z. Wang , T. Fu , H. Wu , Int. J. Heat. Mass. Transfer. 2022, 183, 122143.

[268] S. Gong , P. Cheng , Int. J. Heat. Mass. Transfer. 2015, 80, 206.

[269] Q. Zhang , Y. Chen , Z. Guo , H. Liu , D. Wang , X. Huang , ACS Appl. Mater. Interfaces 2013, 5, 10633. 24080041 10.1021/am403534z

[270] Y. Yang , H. He , Y. Li , J. Qiu , Sci. Rep. 2019, 9, 9961. 31292503 10.1038/s41598-019-46337-y PMC6620340

[271] S.‐H. Park , S. Lee , D. Moreira , P. R. Bandaru , I. Han , D.‐J. Yun , Sci. Rep. 2015, 5, 15430. 26490133 10.1038/srep15430 PMC4651109

[272] D. Zhang , A. Bai , S. Dong , Y. Hu , Phys. Fluids 2024, 36, 062012.

[273] H. E. Jeong , R. Kwak , A. Khademhosseini , K. Y. Suh , Nanoscale 2009, 1, 331. 20648269 10.1039/b9nr00106a

[274] K. Liu , M. Cao , A. Fujishima , L. Jiang , Chem. Rev. 2014, 114, 10044. 24956456 10.1021/cr4006796

[275] Z. Han , X. Feng , Z. Guo , S. Niu , L. Ren , Adv. Mater. 2018, 30, 1704652. 10.1002/adma.201704652 29441617

[276] S. Wang , Z. Yang , G. Gong , J. Wang , J. Wu , S. Yang , L. Jiang , J. Phys. Chem. C 2016, 120, 15923.

[277] S. Yang , J. Ju , Y. Qiu , Y. He , X. Wang , S. Dou , K. Liu , L. Jiang , Small 2013, 10, 294. 23908145 10.1002/smll.201301029

[278] L. Feng , Y. Zhang , J. Xi , Y. Zhu , N. Wang , F. Xia , L. Jiang , Langmuir 2008, 24, 4111. 10.1021/la703821h 18312016

[279] K. Liu , J. Du , J. Wu , L. Jiang , Nanoscale 2012, 4, 768. 22139414 10.1039/c1nr11369k

[280] Z. Han , Z. Mu , B. Li , Z. Wang , J. Zhang , S. Niu , L. Ren , ACS Nano 2016, 10, 8591. 27442422 10.1021/acsnano.6b03884

[281] Y. Yang , G. Zografi , E. Miller , J. Colloid Interface Sci. 1988, 122, 35.

[282] Y. Yang , G. Zografi , E. Miller , J. Colloid Interface Sci. 1988, 122, 24.

[283] M. R. S. Shirazy , S. Blais , L. G. Fréchette , Appl. Surf. Sci. 2012, 258, 6416.

[284] X. Li , L. Zhang , X. Ma , H. Zhang , Surf. Coat. Technol. 2016, 307, 243.

[285] L. Schneider , M. Laustsen , N. Mandsberg , R. Taboryski , Sci. Rep. 2016, 6, 21400. 26892169 10.1038/srep21400 PMC4759530

[286] S. Milles , M. Soldera , B. Voisiat , A. F. Lasagni , Sci. Rep. 2019, 9, 13944. 31558749 10.1038/s41598-019-49615-x PMC6763440

[287] D. Wang , A. Zhao , R. Jiang , D. Li , M. Zhang , Z. Gan , W. Tao , H. Guo , T. Mei , Appl. Surf. Sci. 2012, 259, 93.

[288] C. Luo , T. Tagawa , Int. J. Heat. Mass. Transfer. 2024, 225, 125394.

[289] X. Bai , Q. Yang , Y. Fang , J. Yong , Y. Bai , J. Zhang , X. Hou , F. Chen , Chem. Eng. J. 125930, 2020, 400.

[290] X. Xie , Q. Weng , Z. Luo , J. Long , X. Wei , Int. J. Heat. Mass. Transfer. 2018, 125, 658.

[291] Y. Fu , M. Soldera , W. Wang , S. Milles , K. Deng , B. Voisiat , K. Nielsch , F. A. Lasagni , Sci. Rep. 2020, 10, 22428. 33380738 10.1038/s41598-020-79936-1 PMC7773741

[292] Z. Wang , J. Zhao , A. Bagal , E. C. Dandley , C. J. Oldham , T. Fang , G. N. Parsons , C.‐H. Chang , Langmuir 2016, 32, 8029. 27459627 10.1021/acs.langmuir.6b01864

[293] H. Ems , S. Ndao , Appl. Surf. Sci. 2015, 339, 137.

[294] K. Yin , L. Wang , Q. Deng , Q. Huang , J. Jiang , G. Li , J. He , Nano Micro Lett. 2022, 14, 212. 10.1007/s40820-022-00840-6 PMC8993985 35394233

[295] M. A. Amidu , M. Ali , A. K. Alkaabi , Y. Addad , Sci. Rep. 2023, 13, 7829. 37188733 10.1038/s41598-023-34907-0 PMC10185680

[296] S. L. Tariq , H. M. Ali , M. A. Akram , M. M. Janjua , M. Ahmadlouydarab , Appl. Therm. Eng. 2020, 176, 115305.

[297] M. Ghalambaz , S. A. M. Mehryan , K. A. Ayoubloo , A. Hajjar , M. E. Kadri , O. Younis , M. S. Pour , C. Hulme‐Smith , Molecules 2021, 26, 1491. 33803388 10.3390/molecules26051491 PMC7967206

[298] F. Hassan , A. Hussain , F. Jamil , A. Arshad , H. M. Ali , Energies 2022, 15, 8746.

[299] D. Zou , X. Ma , X. Liu , P. Zheng , Y. Hu , Int. J. Heat. Mass. Transfer. 2018, 120, 33.

[300] Z. Khan , Z. A. Khan , Energy Convers. Manage. 2020, 224, 113349.

[301] Y. Gao , D. Liu , Y. Zhao , D. Yang , L. Zhang , F. Sun , X. Wang , Gels 2025, 11, 516. 40710678 10.3390/gels11070516 PMC12294304

[302] P. T. Saravanakumar , S. P. Arunkumar , B. B. Mansingh , P. M. Kumar , R. Subbiah , V. K. Eswarlal , Mater. Today: Proc. 2022, 50, 1502.

[303] Y. Kang , S.‐G. Jeong , S. Wi , S. Kim , Sol. Energy Mater. Sol. Cells 2015, 143, 430.

[304] M. R. Salem , M. M. Elsayed , A. A. Abd‐Elaziz , K. M. Elshazly , Renewable Energy 2019, 138, 876.

[305] S. P. H. Largani , H. Salimi‐Kenari , S. R. Nabavi , A. A. R. Darzi , J. Energy Storage 2024, 80, 110351.

[306] M. Iasiello , M. Mameli , S. Filippeschi , N. Bianco , Appl. Therm. Eng. 2021, 187, 116572.

[307] X. Hu , X. Gong , F. Zhu , X. Xing , Z. Li , X. Zhang , Renewable Energy 2023, 212C, 227.

[308] V. Safari , B. Kamkari , N. Hewitt , K. Hooman , Therm. Sci. Eng. Prog. 2024, 48, 102401.

[309] R. Aga , L. Davidson , C. Bartsch , E. Heckman , in IEEE 72nd Electronic Components and Technology Conference (ECTC) , San Diego, CA, USA, 2022.

[310] Y. Yao , W. Li , J. Liu , Z. Deng , Adv. Phys.:X 2024, 9, 2324910.

[311] H. Ge , H. Li , S. Mei , J. Liu , Renewable Sustainable Energy Rev. 2013, 21, 331.

[312] H. Li , J. Liu , Front. Energy Power Eng. China 2011, 5, 20.

[313] A. Wee , R. Schneider , S. Aquilino , J. Prosthet. Dent. 1998, 80, 540. 9813803 10.1016/s0022-3913(98)70029-0

[314] F. L. Tan , S. C. Fok , G. Setoh , Int. Commun. Heat Mass Transf. 2010, 37, 1403l.

[315] Y.‐T. Yang , Y.‐H. Wang , Int. J. Therm. Sci. 2012, 51, 155.

[316] M. Jaworski , Appl. Therm. Eng. 2012, 35, 212.

[317] W. Shen , F. L. Tan , S. C. Fok , Int. J. Therm. Sci. 2010, 49, 109.

[318] T. C. Chang , S. Lee , Y. K. Fuh , Y. Peng , Z. Lin , Appl. Therm. Eng. 2017, 112, 1129.

[319] K. Karthikeyan , V. Mariappan , P. Kalidoss , J. M. J. Ganesh , P. V. R. N. Kishore , S. Prathiban , R. Anish , J. Energy Storage 2023, 74, 109442.

[320] A. Arshad , M. Jabbal , F. Hamza , P. Talebizadehsardari , M. A. Bashir , Y. Yan , J. Energy Storage 2022, 48C, 103882.

[321] D. L. Veilleux , E. Goncalves , M. Faghri , Y. Asako , MajidCharmchi , Int. J. Numer. Methods Heat Fluid Flow 2005, 15, 710.

[322] A. Y. Uzan , Y. Kozak , Y. Korin , I. Harary , H. Mehling , G. Ziskind , Int. J. Heat. Mass. Transfer. 2017, 106, 91.

[323] X. Yang , S. Tan , J. Liu , Int. J. Heat. Mass. Transfer. 2016, 100, 899.

[324] H. Ge , J. Liu , Frontiers of Energy and Power Engineering in China 2012, 6, 207.

[325] H. Ge , J. Liu , J. Heat Transfer 2013, 135, 054503.

[326] Z. Xu , X. Li , Z. Zhu , Q. Wang , Y. Chen , T. Ma , Energy Convers. Manage. 2020, 213, 112853.

[327] M. Alipanah , X. Li , Int. J. Heat. Mass. Transfer. 2016, 102, 1159.

[328] Y. Yao , Y. Cui , Z. Deng , Chinese Academy of Sciences 2022, 12, 17217.

[329] Y. Ding , J. J. Klemeš , P. Zhao , M. Zeng , Q. Wang , Energy 2022, 249, 123679.

[330] J.‐X. Wang , H. Lai , M. Zhong , X. Liu , Y. Chen , S. Yao , Small 2023, 7, 2300139. 10.1002/smtd.202300139 37129546

[331] X. Yang , S. Tan , Y. Ding , L. Wang , J. Liu , Y. Zhou , Int. Commun. Heat Mass Transf. 2017, 87, 118.

[332] J.‐X. Wang , J. Qian , N. Wang , H. Zhang , X. Cao , F. Liu , G. Hao , Renewable Energy 2023, 213C, 75.

[333] Y. Yao , S. Chen , J. Ye , Y. Cui , Z. Deng , ACS Appl. Mater. Interfaces 2021, 13, 60660. 34898166 10.1021/acsami.1c18824

[334] C. R. Raj , S. Suresh , V. K. Singh , R. R. Bhavsar , M. Chandrasekar , V. Archita , J. Energy Storage 2021, 35, 102220.

[335] K. Kashiyama , T. Kawaguchi , K. Dong , H. Sakai , N. Sheng , A. Kurniawan , T. Nomura , Energy Storage 2020, 2, e177.

[336] S. Wang , X. Zhao , Z. Wang , Y. Zhang , H. Wang , D. Zou , J. Cleaner Prod. 2023, 417C, 138058.

[337] D. H. Yu , Z. Z. He , Appl. Energy 2019, 247, 503.

[338] J.‐Y. Gao , X.‐D. Zhang , J.‐H. Fu , X.‐H. Yang , J. Liu , Int. J. Heat. Mass. Transfer. 2020, 150, 119366.

[339] J.‐T. Hung , Y.‐K. Chen , H.‐K. Shih , C.‐C. Wang , Int. J. Heat. Mass. Transfer. 2020, 159, 120092.

[340] W. Du , J. Fang , D. Fan , H. Cheng , Int. J. Heat Fluid Flow 2024, 107, 109431.

[341] M. I. Omisanya , Z. Chen , Y. Utaka , Int. J. Heat. Mass. Transfer. 2024, 226, 125471.

[342] H. Wei , X. Sha , L. Chen , Z. Wang , C. Zhang , P. He , W. Q. Tao , Small 2024, 2401393. 10.1002/smll.202401393 38477692

[343] W.‐R. Liao , L.‐H. Chien , M. Ghalambaz , W.‐M. Yan , Int. Commun. Heat Mass Transf. 2019, 108, 104277.

[344] D. Deng , W. Wan , Y. Qin , J. Zhang , X. Chu , Int. J. Heat. Mass. Transfer. 2017, 105, 338.

[345] S. A. Khan , N. Sezer , M. Koç , Appl. Therm. Eng. 2019, 153, 168.

[346] S. A. Khan , N. Sezer , S. Ismail , M. Koç , Energy Convers. Manage. 2019, 195, 1056.

[347] Y. Song , C. D. Díaz‐Marín , L. Zhang , H. Cha , Y. Zhao , E. N. Wang , Adv. Mater. 2022, 34. 10.1002/adma.202200899 35725240

[348] S. Q. Cai , A. Bhunia , Int. J. Heat. Mass. Transfer. 2014, 79, 981.

[349] S. Movaghgharnezhad , J. Darabi , Nanoscale Microscale Thermophys. Eng. 2021, 25, 116.

[350] S. Adera , D. Antao , R. Raj , E. N. Wang , Int. J. Heat. Mass. Transfer. 2016, 101, 280.

[351] J. Fang , H. Cheng , D. Fan , Int. Commun. Heat Mass Transf. 2023, 148, 107018.

[352] D. Fan , J. Fang , W. Tong , W. Du , Q. Li , Cell Rep. Phys. Sci. 2024, 5, 102156.

[353] Y. Zhang , Z. Han , Z. Zhu , Y. Yu , Y. Gao , C. Guo , Case Stud. Therm. Eng. 2023, 52, 103776.

[354] M. Liu , W. Ning , J. Yang , Y. Zhang , Z. Han , G. Meng , C. Guo , H. Lin , B. Jia , Int. Commun. Heat Mass Transf. 2023, 148, 107019.

[355] Y. Zhang , Z. Han , Y. Yu , M. A. Rhamdhani , Y. Gao , C. Guo , Int. J. Heat. Mass. Transfer. 2024, 218.

[356] G. Udaya Kumar , S. Suresh , M. R. Thansekhar , P. Dinesh Babu , Appl. Surf. Sci. 2017, 423, 509.

[357] H. Kim , J. Kim , M. Kim , Int. J. Multiphase Flow 2007, 33, 691.

[358] B. S. Kim , S. Shin , D. Lee , G. Choi , H. Lee , K. M. Kim , H. H. Cho , Int. J. Heat. Mass. Transfer. 2014, 70, 23.

[359] B. S. Kim , H. Lee , S. Shin , G. Choi , H. H. Cho , Appl. Phys. Lett. 2014, 105, 191601.

[360] L. Lou , K. Chen , J. Fan , Materials science and engineering. R: Reports 2021, 146C, 100639. 10.1016/j.mser.2021.100639 PMC8590464 34803231

[361] L. Lou , Z. Kang , H. Zhang , P. Wang , J. Fan , Mater. Today Phys. 2023, 36.

[362] J. Chun , C. Xu , Y. Zhang , Q. Li , R. Wen , X. Ma , ACS Appl. Nano Mater. 2021, 4, 5360.

[363] P.‐X. Jiang , G. Huang , Y. Zhu , Z. Liao , Z. Huang , Int. J. Heat. Mass. Transfer. 2017, 108, 232.

[364] R. Xu , J. Zhou , Z. Liao , X. Li , H. Hu , K. Hu , P. Jiang , Adv. Mater. 2024, 36, 2312765. 10.1002/adma.202312765 38879784

[365] A. P. Raman , E. Rephaeli , S. Fan , M. A. Anoma , L. Zhu , Nature 2014, 515, 540. 25428501 10.1038/nature13883

[366] D. Chae , M. Kim , H. Lim , D. Lee , S. Son , J. Ha , J. Rho , H. Lee , Opt. Mater. 2022, 128, 112273.

[367] H. Ma , K. Yao , S. Dou , M. Xiao , M. Dai , L. Wang , H. Zhao , J. Zhao , Y. Li , Y. Zhan , Sol. Energy Mater. Sol. Cells 2020, 212, 110584.

[368] G. Mabchour , M. Benlattar , K. Saadouni , M. Mazroui , Optik 2020, 214, 164811.

[369] Z. Chen , L. Zhu , A. Raman , S. Fan , Nat. Commun. 2016, 7, 13729. 27959339 10.1038/ncomms13729 PMC5159822

[370] W. Li , Y. Shi , Z. Chen , S. Fan , Nat. Commun. 2018, 9, 4240. 30315155 10.1038/s41467-018-06535-0 PMC6185958

[371] C.‐C. Tsai , R. A. Childers , N. Nan Shi , C. Ren , J. N. Pelaez , G. D. Bernard , N. E. Pierce , N. Yu , Nat. Commun. 2020, 11, 551. 31992708 10.1038/s41467-020-14408-8 PMC6987309

[372] Q. Liu , W. Wu , S. Lin , H. Xu , Y. Lu , W. Song , Opt. Commun. 2019, 450, 246.

[373] F. Xie , W. Jin , J. R. Nolen , H. Pan , N. Yi , Y. An , Z. Zhang , X. Kong , F. Zhu , K. Jiang , S. Tian , T. Liu , X. Sun , L. Li , D. Li , Y.‐F. Xiao , A. Alu , S. Fan , W. Li , Science 2024, 386, 788. 39541474 10.1126/science.adn2524

[374] M. M. Hossain , B. Jia , M. Gu , Adv. Opt. Mater. 2015, 3, 1047.

[375] L. Zhu , A. P. Raman , S. Fan , CLEO: QELS Fundamental Science, San Jose, CA, USA, 2016.

[376] K.‐T. Lin , X. Nian , K. Li , J. Han , N. Zheng , X. Lu , C. Guo , H. Lin , B. Jia , eLight 2023, 3, 22.

[377] Z. Xia , Z. Fang , Z. Zhang , K. Shi , Z. Meng , ACS Appl. Mater. Interfaces 2020, 12, 27241. 32437122 10.1021/acsami.0c05803

[378] P.‐C. Hsu , A. Y. Song , P. B. Catrysse , C. Liu , Y. Peng , J. Xie , S. Fan , Y. Cui , Science 2016, 353, 1019. 27701110 10.1126/science.aaf5471

[379] C. Ziming , W. Fuqiang , G. Dayang , L. Huaxu , S. Yong , Sol. Energy Mater. Sol. Cells 2020, 213, 110563.

[380] Y. Liu , S. Son , D. Chae , P.‐H. Jung , H. Lee , Sol. Energy Mater. Sol. Cells 2020, 213, 110561.

[381] W. Wu , S. Lin , M. Wei , J. Huang , H. Xu , Y. Lu , W. Song , Sol. Energy Mater. Sol. Cells 2020, 210, 110512.

[382] C. Zou , G. Ren , M. M. Hossain , S. Nirantar , W. Withayachumnankul , T. Ahmed , M. Bhaskaran , S. Sriram , M. Gu , C. Fumeaux , Adv. Opt. Mater. 2017, 5, 201700460.

[383] Y. Zhai , Y. Ma , S. N. David , D. Zhao , R. Lou , G. Tan , R. Yang , X. Yin , Science 2017, 355, 1062. 28183998 10.1126/science.aai7899

[384] N. N. Shi , C.‐C. Tsai , M. J. Carter , J. Mandal , A. C. Overvig , M. Y. Sfeir , M. Lu , C. L. Craig , G. D. Bernard , Y. Yang , N. Yu , Light Sci. Appl. 2018, 7, 37. 30839604 10.1038/s41377-018-0033-x PMC6107007

[385] D. Xie , Z. Yang , X. Liu , S. Cui , H. Zhou , T. Fan , Soft Matter 2019, 15, 4294. 31095159 10.1039/c9sm00566h

[386] J. Lee , Y. Jung , M. Lee , J. S. Hwang , J. Guo , W. Shin , J. Min , K. R. Pyun , H. Lee , Y. Lee , J. Shiomi , Y.‐J. Kim , B.‐W. Kim , S. H. Ko , Nanoscale Horiz. 2022, 7, 1054. 35775456 10.1039/d2nh00166g

[387] Z. Cheng , H. Han , F. Wang , Y. Yan , X. Shi , H. Liang , X. Zhang , Y. Shuai , Nano Energy 2021, 89, 106377.

[388] Y. Zhu , D. Wang , C. Fang , P. He , Y.‐H. Ye , Polymers 2019, 11, 1203. 31323830 10.3390/polym11071203 PMC6680741

[389] B. Guha , C. Otey , C. B. Poitras , S. Fan , M. Lipson , Nano Lett. 2012, 12, 4546. 22891815 10.1021/nl301708e

[390] L. Zhu , A. P. Raman , S. Fan , Proc. Natl. Acad. Sci. USA 2015, 112, 12282. 26392542 10.1073/pnas.1509453112 PMC4603484

[391] J. Mandal , Y. Fu , A. C. Overvig , M. Jia , K. Sun , N. N. Shi , H. Zhou , X. Xiao , N. Yu , Y. Yang , Science 2018, 362, 315. 30262632 10.1126/science.aat9513

[392] L. Zhu , A. Raman , K. X. Wang , M. A. Anoma , S. Fan , Optica 2014, 1, 010032.

[393] D. Zhao , A. Aili , Y. Zhai , J. Lu , D. Kidd , G. Tan , X. Yin , R. Yang , Joule 2019, 3, 111.

[394] E. A. Goldstein , A. P. Raman , S. Fan , Nat. Energy 2017, 2, 17143.

[395] S. Atiganyanun , J. B. Plumley , S. J. Han , K. Hsu , J. Cytrynbaum , T. L. Peng , S. M. Han , S. E. Han , ACS Photonics 2018, 5, 1181.

[396] D. Shen , C. Yu , W. Wang , Appl. Therm. Eng. 2020, 176, 115479.

[397] J. K. Tong , X. Huang , S. V. Boriskina , J. Loomis , Y. Xu , G. Chen , ACS Photonics 2015, 2, 769.

[398] W. Wei , Y. Zhu , Q. Li , Z. Cheng , Y. Yao , Q. Zhao , P. Zhang , X. Liu , Z. Chen , F. Xu , Y. Gao , Solar Energy Mater. Solar Cells 2020, 211, 110525.

[399] X. Nian , K.‐T. Lin , K. Li , J. Hei , J. Han , Y. Li , C. Guo , H. Lin , J. Zheng , B. Jia , Engineering 2024, 49, 122.

[400] Y. Xin , C. Li , W. Gao , Y. Chen , Mater. Today 2025, 83, 355.

[401] T. Yu , R. Liu , Z. Yang , S. Yang , Z. Ye , J. Lu , Appl. Energy 2025, 377A, 124436.

[402] T. Wang , Y. Liu , Y. Dong , X. Yin , D. Lei , J.‐G. Dai , Adv. Mater. 2025, 37, 2414300. 40040298 10.1002/adma.202414300 PMC12004913

[403] Y. Park , B. Zhao , S. Fan , Nano Lett. 2021, 22, 448. 34939814 10.1021/acs.nanolett.1c04288

[404] Z. Chen , S. Yu , B. Hu , R. Hu , Int. J. Heat. Mass. Transfer. 2023, 209, 124149.

[405] J. Dong , W. Zhang , L. Liu , Appl. Phys. Lett. 2021, 119, 021104. 10.1063/5.0064020 PMC8432617 34548671

[406] L. Zhu , S. Fan , Phys. Rev. B 2014, 90, 220301(R).

[407] Y. Hu , Y. Sun , Z. Zheng , J. Song , K. Shi , X. Wu , Int. J. Heat. Mass. Transfer. 2022, 189, 122666.

[408] B. Xiao , Z. Zheng , C. Gu , Y. Xuan , J. Quant. Spectrosc. Radiat. Transfer 2023, 303, 108588.

[409] H. Liu , K. Yu , K. Zhang , Q. Ai , M. Xie , X. Wu , Int. J. Heat. Mass. Transfer. 2023, 210, 124206.

[410] J. Zhang , B. Yang , K. Yu , K. Zhang , H. Liu , X. Wu , AIP Adv. 2023, 13, 045315.

[411] M. Luo , X. Pu , Y. Xiao , Int. J. Heat. Mass. Transfer. 2024, 218, 124816.

[412] J. Wu , B. Wu , Z. Wang , X. Wu , Opt. Laser Technol. 2023, 158, 108907.

[413] W.‐X. Zhang , J.‐Y. Sui , J.‐H. Zou , H.‐F. Zhang , Int. Commun. Heat Mass Transf. 2025, 160, 108365.

[414] X. Wu , R. Liu , H. Yu , B. Wu , J. Quant. Spectrosc. Radiat. Transfer 2021, 272, 107794.

[415] J. Fang , J. Zou , T. Liu , Y. Wang , X. Sun , X. Wu , Y. Wu , D. Zhang , Int. J. Heat. Mass. Transfer. 2024, 223, 125229.

[416] X. Wu , ES Energy and Environment 2021, 12, 46.

[417] J. Wu , B. Wu , K. Shi , X. Wu , C. Fu , Int. J. Therm. Sci. 2023, 187, 108172.

[418] H. Zou , B. Wang , J. Wu , Int. J. Heat. Mass. Transfer. 2024, 231, 125819.

[419] J. Fang , M. Wang , T. Liu , J. Yue , X. Sun , Y. Wu , D. Zhang , Int. J. Therm. Sci. 2024, 195, 108602.

[420] J. Fang , J. Zou , T. Liu , M. Wang , X. Sun , Y. Wu , D. Zhang , Appl. Phys. Lett. 2024, 124, 171702.

[421] Q. Sun , G. Zhi , S. Zhou , X. Dong , Q. Shen , R. Tao , J. Qi , Adv. Mater. Technol. 2024, 9, 2400263.

[422] Y. Su , Y. Li , M. Qi , S. Guenneau , H. Li , J. Xiong , Phys. Rev. Appl. 2023, 20, 034013.

[423] S. Yang , J. Wang , G. Dai , F. Yang , J. Huang , Phys. Rep. 2021, 908, 1.

[424] H. Sadique , Q. M., Samsher , Int. J. Heat. Mass. Transfer. 2022, 194, 123063.

[425] Y. Zhang , Z. Han , S. Wu , A. Rhamdhani , C. Guo , G. Brooks , Int. J. Heat. Mass. Transfer. 2022, 185, 122380.

[426] H. Zhao , X. Yang , C. Wang , R. Lu , T. Zhang , H. Chen , X. Zheng , Mater. Today Phys. 2023, 30, 100941.

[427] R.‐H. Yang , S. An , W. Shang , T. Deng , Acta Phys. Sin. 2022, 71, 024401.

[428] T. Luo , C. Zhu , B. Li , X. Shen , G. Zhu , iScience 2025, 28, 111630. 39935458 10.1016/j.isci.2024.111630 PMC11810695

[429] Y. Wang , W. Sha , M. Xiao , C.‐W. Qiu , L. Gao , Adv. Mater. 2023, 35, 2302387. 10.1002/adma.202302387 37394737

[430] S. So , D. Lee , T. Badloe , J. Rho , Opt. Mater. Express 2021, 11, 1863.

[431] J. Luo , J. Lee , J. Appl. Phys. 2024, 135, 244503.

[432] J. Schmidt , M. R. G. Marques , S. Botti , M. A. L. Marques , npj Comput. Mater. 2019, 5, 83.

[433] L. Chen , W. Jin , J. Zhang , S. X.‐D. Tan , IEEE Transactions on Computer‐Aided Design of Integrated Circuits and Systems 2023, 42, 1.

[434] P. Zhang , D.‐W. Wang , W.‐S. Zhao , B. You , J. Liu , C. Qian , H.‐B. Xu , IEEE Trans. Compon., Packag., Manuf. Technol. 2023, 13.

[435] J. Zhang , S. Sadiqbatcha , L. Chen , C. Thi , S. Sachdeva , H. Amrouch , S. X.‐D. Tan , Integration 2023, 89C, 73.

[436] T. Bücher , H. Amrouch , IEEE Access 2022, 10, 21970.

[437] S. Pagani , P. D. S. Manoj , A. Jantsch , IEEE Trans. Comput.‐Aided Des. Integr. Circuits Syst. 2020, 39, 101.

[438] Y. Wang , M. Triki , X. Lin , A. C. Ammari , M. Pedram , 14th International Symposium on Quality Electronic Design (ISQED) , Santa Clara, CA, USA, 2013.

[439] S. A. Khan , C. Eze , K. Dong , A. R. Shahid , M. S. Patil , S. Ahmad , I. Hussain , J. Zhao , Int. Commun. Heat Mass Transf. 2022, 136C, 106209.

[440] Z. Wei , X. Yang , Y. Li , H. He , W. Li , D. U. Sauer , Energy. Storage. Mater. 2023, 56C, 62.

[441] Z. Wei , R. Song , D. Ji , Y. Wang , F. Pan , Appl. Therm. Eng. 2024, 236, 121544.

[442] L. Sun , G. Li , Q. S. Hua , Y. Jin , Renewable Energy 2020, 147, 1642.

[443] G. Şenol , F. Selimefendigil , H. F. Öztop , Int. J. Hydrogen. Energy. 2024, 68, 1178.

[444] K. T. U. W. Hornik , A. Vienna , M. Stinchcombe , H. White , Neural Networks 1989, 2, 359.

[445] T. Zhan , L. Fang , Y. Xu , Sci. Rep. 2017, 7, 7109. 28769034 10.1038/s41598-017-07150-7 PMC5540921

[446] Y. Liu , N. Dinh , Y. Sato , B. Niceno , Appl. Therm. Eng. 2018, 144C, 305.

[447] J. J. García‐Esteban , J. Bravo‐Abad , J. C. Cuevas , Phys. Rev. Appl. 2021, 16, 064006.

[448] C. Zhu , E. A. Bamidele , X. Shen , G. Zhu , B. Li , Chem. Rev. 2024, 124, 4258. 38546632 10.1021/acs.chemrev.3c00708 PMC11009967

[449] C. Zhu , X. Shen , G. Zhu , B. Li , Chin. Phys. Lett. 2023, 40, 81.

[450] D. Stoecklein , C.‐Y. Wu , D. Kim , D. D. Carlo , B. Ganapathysubramanian , Phys. Fluids 2016, 28.

[451] D. Stoecklein , M. Davies , N. Wubshet , J. Le , B. Ganapathysubramanian , J. Fluids Eng. 2017, 139, 031402.

[452] J. Yang , R. Sarathy , J. Lee , Neural Networks 2018, 98, 122.29227961

[453] Z. Yang , Z. Jiang , H. Lin , X. Fan , C. Wu , E. Y. Lam , H. K. H. So , H. C. Shum , Sci. Adv. 2025, 11. 10.1126/sciadv.adx2826 PMC12309690 40737418

[454] C. Yang , X. Liu , X. Song , L. Zhang , Lab Chip 2024, 24, 4514. 39206574 10.1039/d4lc00566j

[455] Y. Cheng , Y. Yu , F. Fu , J. Wang , L. Shang , Z. Gu , Y. Zhao , ACS Appl. Mater. Interf. 2016, 8, 1080. 10.1021/acsami.5b11445 26741731

[456] Y. Zuo , X. He , Y. Yang , D. Wei , J. Sun , M. Zhong , R. Xie , H. Fan , X. Zhang , Acta Biomater. 2016, 38, 153. 27130274 10.1016/j.actbio.2016.04.036

[457] D. W. Martinez , M. T. Espino , H. M. Cascolan , J. L. Crisostomo , J. R. C. Dizon , Key Eng. Mater. 27, 2022, 913.

[458] A. Jhinkwan , S. Kalsi , in Advancements in Civil Engineering: Cosmec‐2021 , Orlando, FL, USA, 2021.

[459] X. Y. Zheng , J. Deotte , M. P. Alonso , G. R. Farquar , T. H. Weisgraber , S. Gemberling , H. Lee , N. Fang , C. M. Spadaccini , Rev. Sci. Instrum. 2013, 84, P019902. 10.1063/1.4769050 23278017

[460] T. Hu , W. Wang , Intell. Build. Int. 2023, 16, 359.

[461] Y. Bao , N. Paunović , J. C. Leroux , Adv. Funct. Mater. 2022, 32, 2109864.

[462] L. Rodríguez‐Pombo , X. Xu , A. Seijo‐Rabina , J. J. Ong , C. Alvarez‐Lorenzo , C. Rial , D. Nieto , S. Gaisford , A. W. Basit , A. Goyanes , Addit. Manuf. 2022, 52, 102673.

[463] J. Gehlen , W. Qiu , G. N. Schadli , R. Muller , X. H. Qin , Acta Biomater. 2023, 156, 49. 35718102 10.1016/j.actbio.2022.06.020

[464] M. Farsari , B. N. Chichkov , Nat. Photonics 2009, 3, 450.

[465] R. Raman , B. Bhaduri , M. Mir , A. Shkumatov , M. K. Lee , G. Popescu , H. Kong , R. Bashir , Adv. Healthcare Mater. 2016, 5, 610. 10.1002/adhm.201500721 26696464

[466] Q. Ge , Z. Li , Z. Wang , K. Kowsari , W. Zhang , X. He , J. Zhou , N. X. Fang , Int. J. Extreme Manuf. 2020, 2, 022004.

[467] B. E. Kelly , I. Bhattacharya , H. Heidari , M. Shusteff , C. M. Spadaccini , H. K. Taylor , Science 2019, 363, 1075. 30705152 10.1126/science.aau7114

[468] M. Regehly , Y. Garmshausen , M. Reuter , N. F. Konig , E. Israel , D. P. Kelly , C. Y. Chou , K. Koch , B. Asfari , S. Hecht , Nature 2020, 588, 620. 33361791 10.1038/s41586-020-3029-7

[469] D. Loterie , P. Delrot , C. Moser , Nat. Commun. 2020, 11, 852. 32051409 10.1038/s41467-020-14630-4 PMC7015946

[470] J. T. Toombs , M. Luitz , C. C. Cook , S. Jenne , C. C. Li , B. E. Rapp , F. Kotz‐Helmer , H. K. Taylor , Science 2022, 376, 308. 35420940 10.1126/science.abm6459

[471] V. Hahn , P. Rietz , F. Hermann , P. Müller , C. Barner‐Kowollik , T. Schlöder , W. Wenzel , E. Blasco , M. Wegener , Nat. Photonics 2022, 16, 784.

[472] Q. Geng , D. Wang , P. Chen , S. C. Chen , Nat. Commun. 2019, 10, 2179. 31097713 10.1038/s41467-019-10249-2 PMC6522551

[473] C. Vidler , K. Crozier , D. Collins , Microsyst. Nanoeng. 2023, 9, 67. 37251709 10.1038/s41378-023-00537-9 PMC10212948

[474] C. Vidler , M. Halwes , K. Kolesnik , P. Segeritz , M. Mail , A. J. Barlow , E. M. Koehl , A. Ramakrishnan , L. M. C. Aguilar , D. R. Nisbet , D. J. Scott , D. E. Heath , K. B. Crozier , D. J. Collins , Nature 2024, 634, 1096. 39478212 10.1038/s41586-024-08077-6 PMC11525192

[475] J. Liu , W. Li , Y. Guo , H. Zhang , Z. Zhang , Composites, Part A 2019, 120, 140.

[476] Y. Liu , Y. Ding , Z. Liu , X. Li , S. Tian , L. Fan , J. Xie , L. Xu , J. Lee , J. Li , L. Yang , PhotoniX 2024, 5, 6.

[477] S. Sharratt , C. Peng , Y. S. Ju , Int. J. Heat. Mass. Transfer. 2012, 55, 6163.

[478] C. Li , C. Yu , D. Hao , L. Wu , Z. Dong , L. Jiang , Adv. Funct. Mater. 2018, 28, 1707490.

[479] Y. Li , X. Jin , W. Li , J. Niu , X. Han , X. Yang , W. Wang , T. Lin , Z. Zhu , Sci. China Mater. 2021, 65, 1057.

[480] D. Žalec , M. Može , M. Zupančič , I. Golobič , Case Stud. Therm. Eng 2024, 57.

[481] C. Zheng , A. Hu , T. Chen , K. D. Oakes , S. Liu , Appl. Phys. A 2015, 121, 163.

[482] D. F. Hanks , Z. Lu , J. Sircar , T. R. Salamon , D. S. Antao , K. R. Bagnall , B. Barabadi , E. N. Wang , Microsyst. Nanoeng. 2018, 4, 1. 31057891 10.1038/s41378-018-0004-7 PMC6220170

[483] X. Wen , Y. Gao , H. Zhang , Y. Yang , Micromachines 2024, 15, 394. 38542641 10.3390/mi15030394 PMC10971910

[484] A. Fernández , A. Francone , L. H. Thamdrup , A. Johansson , B. Bilenberg , T. Nielsen , M. Guttmann , C. M. Sotomayor Torres , N. Kehagias , ACS Appl. Mater. Interfaces 2017, 9, 7701. 28085240 10.1021/acsami.6b13615

[485] J.‐F. Bryche , M. Vega , J. Moreau , P.‐L. Karsenti , P. Bresson , M. Besbes , P. Gogol , D. Morris , P. G. Charette , M. Canva , ACS Photonics 2023, 10, 1177.

[486] S. N. Sanders , T. H. Schloemer , M. K. Gangishetty , D. Anderson , M. Seitz , A. O. Gallegos , R. C. Stokes , D. N. Congreve , Nature 2022, 604, 474. 35444324 10.1038/s41586-022-04485-8

[487] X. Zhang , A. Du , Y. Luo , C. Lv , Y. S. Zhang , S. Yan , Y. Wu , J. Qiu , Y. He , L. Wang , Q. Li , Surf. Interfaces 2022, 33.

[488] Z. Chen , L. Song , Y. Wang , H. Tao , Z. Liu , T. Wang , F. Ye , Y. He , J. Lin , Appl. Surf. Sci. 2024, 655, 159454.

[489] S. Ndao , Y. Peles , M. K. Jensen , Int. J. Heat. Mass. Transfer. 2009, 52, 4317.

[490] T. Brunschwiler , B. Michel , H. Rothuizen , U. Kloter , B. Wunderle , H. Oppermann , H. Reichl , Microsyst. Technol. 2009, 15, 57.

[491] T. Tiwei , H. Oprins , V. Cherman , G. Van Der Plas , D. De Wolf , E. Beyne , M. Baelmans , 63rd IEEE International Electron Devices Meeting, IEDM 2017 , San Francisco, CA, USA, 2018.

[492] J. Ditri , J. Hahn , R. Cadotte , M. McNulty , D. Luppa , ASME 2015 International Technical Conference and Exhibition on Packaging and Integration of Electronic and Photonic Microsystems , InterPACK, San Francisco, CA, USA, 2015.

[493] Y. Han , B. L. Lau , X. Zhang , Y. C. Leong , K. F. Choo , IEEE Trans. Compon., Packag., Manuf. Technol. 2014, 4, 1441.

[494] C. Monachon , L. Weber , C. Dames , Annu. Rev. Mater. Res. 2016, 46, 433.

[495] A. I. Bezuglyj , V. A. Shklovskij , Low Temp. Phys. 2016, 42, 636.

[496] M. M. Waldrop , Nature 2016, 530, 144. 26863965 10.1038/530144a

[497] M. Hao , J. Li , S. Park , S. Moura , C. Dames , Nat. Energy 2018, 3, 899.

[498] Y. Zhu , J. Xie , A. Pei , B. Liu , Y. Wu , D. Lin , J. Li , H. Wang , H. Chen , J. Xu , A. Yang , C.‐L. Wu , H. Wang , W. Chen , Y. Cui , Nat. Commun. 2019, 10, 2067. 31061393 10.1038/s41467-019-09924-1 PMC6502817

[499] X. Feng , D. Ren , X. He , M. Ouyang , Joule 2020, 4, 743.

[500] R. Prasher , Appl. Phys. Lett. 2009, 94, 041905.

[501] B. V. Budaev , D. B. Bogy , Phys. Rev. A 2010, 374, 4774.

[502] B. V. Budaev , D. B. Bogy , J. Phys. A:Math. Theor. 2010, 43, 425201.

[503] R. O. Pohl , E. T. Swartz , Appl. Phys. Lett. 1987, 51, 2200.

[504] P. E. Hopkins , J. L. Kassebaum , P. M. Norris , J. Appl. Phys. 2009, 105, 023710.

[505] P. E. Hopkins , J. C. Duda , C. W. Petz , J. A. Floro , Phys. Rev. B 2011, 84, 035438.

[506] T. Beechem , M. A. Rodriguez , J. C. Duda , J. F. Ihlefeld , P. E. Hopkins , K. Hattar , E. S. Piekos , Phys. Rev. B 2011, 84, 125408. 10.1103/PhysRevLett.109.195901 23215405

[507] M. E. Lumpkin , W. M. Saslow , W. M. Visscher , Phys. Rev. B 1978, 17, 4295.

[508] C. Steinbrüchel , Zeitschrift für Physik B 1976, 24, 293.

[509] J.‐S. Wangt , B. K. Agarwalla , H. Li , J. Thingna , Front. Phys. 2014, 9, 673.

[510] J. Wang , J. Wang , J. Lu , Eur. Phys. J. B 2008, 62, 381.

[511] K. Sääskilahti , J. Oksanen , J. Tulkki , Phys. Rev. B 2014, 90, 134312.

[512] K. Sääskilahti , J. Oksanen , S. G. Volz , J. J. Tulkki , Phys. Rev. B 2015, 91, 115426.

[513] K. Sääskilahti , J. Oksanen , J. Tulkki , S. Volz , Phys. Rev. E 2016, 93, 052141. 27300863 10.1103/PhysRevE.93.052141

[514] X. Ran , Y. Guo , M. Wang , Int. J. Heat. Mass. Transfer. 2018, 123, 616.

[515] M. Jeng , R. Yang , D. Song , G. Chen , J. Heat Transfer 2008, 130, 042410.

[516] R. B. Wilson , B. A. Apgar , L. W. Martin , D. G. Cahill , Opt. Express 2012, 20, 28829. 23263123 10.1364/OE.20.028829

[517] D. Liu , R. Xie , N. Yang , B. Li , J. T. L. Thong , Nano Lett. 2014, 14, 806. 24382310 10.1021/nl4041516

[518] X.‐S. Wu , W.‐T. Tang , X.‐F. Xu , Acta Phys. Sin. 2020, 69.

[519] G. Xiong , J. Wang , D. Ma , L. Zhang , EPL 2019, 128, 54007.

[520] J. Liu , H. Feng , J. Dai , K. Yang , G. Chen , S. Wang , D. Jin , X. Liu , Chem. Eng. J. 2023, 469C, 143963.

[521] B. Wei , W. Luo , J. Du , Y. Ding , Y. Guo , G. Zhu , Y. Zhu , B. Li , SusMat 2024, 4, 239.

[522] Y. Yan , Y. Zhuang , H. Ouyang , J. Hao , X. Han , Int. J. Heat. Mass. Transfer. 2024, 226, 125455.

[523] Z. Zheng , S. Wei , Y. Yang , D. Zhang , D. Yang , W. Li , J. Guo , Adv. Eng. Mater. 2023, 25, 2201817.

[524] W. Luo , B. Wei , T. Luo , B. Li , G. Zhu , Small 2024, 20, 2406574. 10.1002/smll.202406574 39363667

[525] X. Xu , J. Chen , J. Zhou , B. Li , Adv. Mater. 2018, 30, 1705544. 10.1002/adma.201705544 29573283

[526] G. Liu , K. Shang , S. Chen , J. Shen , Compos. Sci. Technol. 2025, 264, 111120.

[527] G. Liu , Y. Zhang , H. Li , J. Shen , J. Energy Storage 2025, 118, 116279.

[528] K. Lu , C. Wang , C. Wang , X. Fan , F. Qi , H. He , Manuf. Rev. 2023, 10.

[529] Z. Maqbool , M. Hanief , M. Parveez , J. Energy Storage 2023, 60C, 106591.

[530] W. Song , Y. Xu , L. Xue , H. Li , C. Guo , Micromachines 2021, 12, 1080. 34577723 10.3390/mi12091080 PMC8465721

[531] Y. Alihosseini , M. Zabetian Targhi , M. M. Heyhat , N. Ghorbani , Appl. Therm. Eng. 2020, 170, 114974.

[532] H. Kwon , Q. Wu , D. Kong , S. Hazra , K. Jiang , S. Narumanchi , H. Lee , J. W. Palko , E. M. Dede , M. Asheghi , K. E. Goodson , Int. Commun. Heat Mass Transf. 2024, 156, 107592.

[533] B. Yu , B. Li , Phys. Rev. E 2006, 73, 6302.

[534] C. Zhu , T. Luo , B. Li , X. Shen , G. Zhu , J. Appl. Phys. 2024, 135, 195103.

[535] X. Li , P. Hua , Q. Sun , Nat. Commun. 2023, 14, 7982. 38042868 10.1038/s41467-023-43611-6 PMC10693641

[536] J. Zhang , Y. Zhu , S. Cheng , S. Yao , Q. Sun , Appl. Energy 2023, 351, 121839.

[537] J. Zhang , M. Chen , W. Luo , B. Wei , T. Luo , X. Shen , B. Li , G. Zhu , Nat. Sustain. 2025, 8, 651.

[538] M. Schmidt , A. Schütze , S. Seelecke , Int. J. Refrig. 2015, 54, 88.

[539] H. Ossmer , C. Chluba , S. Kauffmann‐Weiss , E. Quandt , M. Kohl , APL Mater. 2016, 4, 064102.

[540] J. Xu , F. Bruederlin , L. Bumke , H. Ossmer , E. Quandt , S. Miyazaki , M. Kohl , Shape. Mem, Superlast. 2024, 10, 119.

[541] C. Ludwig , J. Leutner , O. Prucker , J. Rühe , M. Kohl , J. Phys Energy 2024, 6, 015009.

[542] S. Yao , P. Dang , Y. Li , Y. Wang , X. Zhang , Y. Liu , S. Qian , D. Xue , Y.‐L. He , Nat. Commun. 2024, 15, 7203. 39169046 10.1038/s41467-024-51632-y PMC11339461

