# Supplementary material for: Beyond Conventional Cooling: Advanced Micro/Nanostructures for Managing Extreme Heat Flux
Source: Adv Mater. 2025 Nov 17;38(5):e04706. doi: 10.1002/adma.202504706 (PMC12822545; doi:10.1002/adma.202504706)
Supplement: Supplementary file 1 — Supporting Information [file ADMA-38-e04706-s001.docx]

**RASupplementary Information for:**

Beyond Conventional Cooling: Advanced Micro/Nanostructures for Managing Extreme Heat Flux

**Author list**

Yuankun Zhang, Huajie Li, Yuhang Zhou, Jun Ma, Keng-Te Lin, Han Lin*, Chunsheng Guo* and Baohua Jia*

**Affiliations**

Centre for Omniscale Thermal Management and Comprehensive Energy Utilization (OTM-EU), School of Mechanical, Electrical & Information Engineering, Shandong University, Weihai 264209, China

School of Science, Computing and Engineering Technologies, Swinburne University of Technology, Hawthorn, Victoria 3122, Australia

Center for Atomaterials and Nanomanufacturing, School of Science, RMIT University, Melbourne, Victoria 3000, Australia

Shenzhen Research Institute of Shandong University, Shenzhen 518057, China

ARC Training Centre for Surface Engineering for Advanced Materials (SEAM), RMIT University, Melbourne, Victoria 3000, Australia

**Contents**

**Supplementary Notes 1 |** Calculation of heat transfer performance in three modes for typical cases.

**Supplementary Table S1 |** Cooling performance of typical micro/nanostructures (enhanced convection/vaporization).

**Supplementary Table S2 |** Cooling performance of typical micro/nanostructures (radiative cooling).

**Supplementary References**

**Supplementary Notes 1 | Calculation of heat transfer performance in three modes for typical cases.**

**Case 1: Enclosed chip-level electronics (Desktop CPU)**

*Parameters*

Heating chip *T*_s_=358 K, heat spreader surface *T*_hs_=338 K (*ΔT*_TIM_=20 K), case air *T*_air_=298 K (*ΔT*_air_=40 K).

Thermal interface material (TIM): *k*=5 W/(m·K), *δ*=50 μm.

Air flow: *v=*2 m/s. Heat spreader length *L=*0.02 m.

Liquid cold plate: water, *D*=4 mm, *v=*3 m/s, *T*_water_=323 K (*ΔT*_liq_=15 K).

*
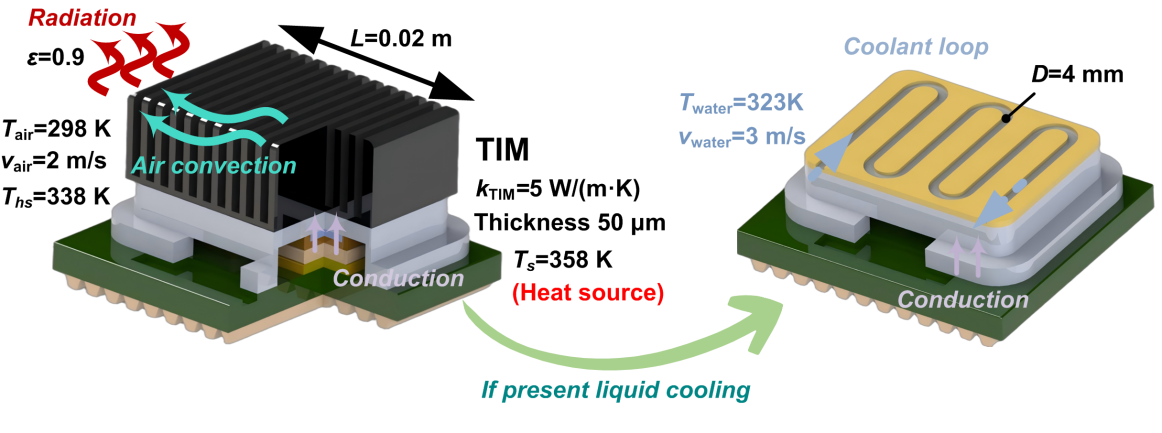
*

*Results*

**Conduction** (TIM): $\text{h}_{\text{cond}}\text{=}\frac{k}{\delta}=\frac{\text{5}}{\text{50×}\text{10}^{\text{-6}}}$=1×10^5^ W/(m^2^·K).

${\text{q}^{\text{''}}}_{\text{cond}}\text{=}\text{h}_{\text{cond}}\text{∙}\text{Δ}\text{T}_{\text{TIM}}$=2×10^6^ W/m^2^.

where ${\text{q}\text{''}}_{\text{cond}}$ and${\text{ }\text{h}}_{\text{cond}}$ are conductive heat flux density (W/m^2^) and heat transfer coefficient (W·m^-2^·K^-1^), *ΔT*_TIM_ is the temperature difference between the heat source and TIM (K). *δ* is the thickness of the TIM (m), and *k* is the thermal conductivity of the TIM (W·m^-1^·K^-1^).

**Air convection**: $\text{Re}$=$\frac{v_{air}L}{\upsilon_{air}}=\frac{\text{2∙0.02}}{\text{1.6×}\text{10}^{\text{-5}}}$=2.5×10^3^

where *Re* represents Reynolds number, *v*_air_ is the air flow rate (m/s), *υ*_air_ is the kinematic viscosity of the air, taken as 1.6×10^-5^ m^2^/s. Since the *Re* number is less than 5×10^5^, the Nusselt number correlation for laminar flow over the flat plate is adopted^[1]^.

*Nu*=$0.664{Re}^{1/2}{Pr}^{1/3}=$29.6

Thereby,

$\text{h}_{\text{conv,air}}\text{=}\frac{Nuk_{air}}{L}=\frac{\text{29.6∙0.026}}{\text{0.02}}\text{=40}$ W/(m^2^·K).

${\text{q}^{\text{''}}}_{\text{conv,air}}\text{=}\text{h}_{\text{conv,air}}\text{∙}\text{Δ}\text{T}_{\text{air}}$=1.4×10^3^ W/m^2^.

where ${\text{q}\text{''}}_{\text{conv,air}}$ and${\text{ }\text{h}}_{\text{conv,air}}$ are heat flux density and heat transfer coefficient of air convection, *Pr* is the Prandtl number of air at ambient temperature, taken as 0.71, *Nu* is the Nusselt number, *k*_air_ is the thermal conductivity of air, taken as 0.026 W/(m·K).

**Liquid convection** (water): $\text{Re}$=$\frac{v_{water}D}{\upsilon_{water}}=\frac{\text{3∙0.004}}{\text{1×}\text{10}^{\text{-6}}}$=1.2×10^4^

where *v*_water_ is the air flow rate (m/s), *υ*_water_ is the kinematic viscosity of the water, taken as 1.0×10^-6^ m^2^/s. Since the *Re* number is higher than 2300, the Nusselt number correlation for turbulence flow within a tube is applied^[2]^.

*Nu*=$0.023{Re}^{0.8}{Pr}^{0.4}$=91.9

where *Pr* is the Prandtl number of water, taken as 7.0. Thereby,

$\text{h}_{\text{conv,liq}}\text{=}\frac{Nuk_{water}}{D}=\frac{\text{91.}\text{9}\text{∙0.6}}{\text{0.004}}\text{=1.38}\text{×}$10^4^ W/(m^2^·K)

${\text{q}^{\text{''}}}_{\text{conv,liq}}\text{=}\text{h}_{\text{conv,liq}}\text{∙}\text{Δ}\text{T}_{\text{liq}}$=2.07×10^5^ W/m^2^

where ${\text{q}\text{''}}_{\text{conv,water}}$ and${\text{ }\text{h}}_{\text{conv,water}}$ are heat flux density and heat transfer coefficient of water flow convection.

**Radiation**: ${\text{q}^{\text{''}}}_{\text{rad}}\text{=}\text{εσ}\left( T_{hs}^{4}-T_{\infty}^{4} \right)\text{=}\text{0.88∙5.67×}\text{10}^{\text{-8}}\text{∙}\text{(338}^{\text{4}}\text{-}\text{298}^{\text{4}}\text{)}\text{≈}\text{258 W/}\text{m}^{\text{2}}$

where ${\text{q}^{\text{''}}}_{\text{rad}}$ is the radiative heat flux density. *ε* is the emissivity of the heat spreader (black anodized aluminum fins), taken as 0.88. *σ* is Stefan-Boltzmann constant, taken as 5.67×10^-8^W/(m^2^·K^4^).

$\text{h}_{\text{rad}}\text{=}\frac{{q''}_{rad}}{\Delta T_{\mathrm{air}}}\text{≈}\text{6.5 }$W/(m^2^·K)，which is the radiative heat transfer coefficient.

**Case 2: Outdoor wearables**

*Parameters*

Heating source *T*_s_=323 K, *T*_shell_=313 K (*ΔT*_shell_=10 K). *T*_air_=298 K (*ΔT*_air_=15 K).

Shell material *k*=0.2 W/(m·K), thickness *δ*=1 mm,

Wind: *v=*1 m/s. characteristic length *L*=0.05 m.

Liquid: not available. (no coolant loop)


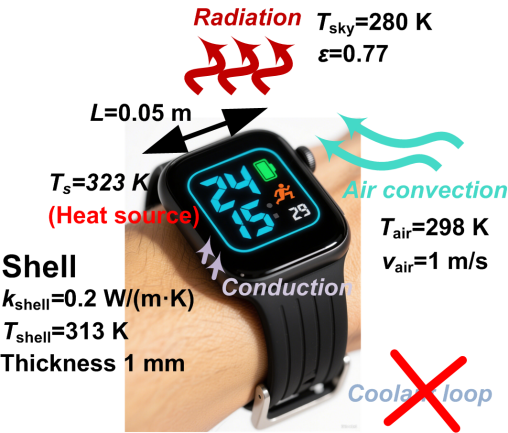


*Results*

**Conduction** (via shell): $\text{h}_{\text{cond}}\text{=}\frac{k}{\delta}\text{=}\frac{\text{0.2}}{\text{1×}\text{10}^{\text{-3}}}$=200 W/(m^2^·K).

${\text{q}^{\text{''}}}_{\text{cond}}\text{=}\text{h}_{\text{cond}}\text{∙}\text{Δ}\text{T}_{\text{shell}}$=2×10^3^ W/m^2^.

where ${\text{q}\text{''}}_{\text{cond}}$ and${\text{ }\text{h}}_{\text{cond}}$ are conductive heat flux density and heat transfer coefficient, *ΔT*_shell_ is the temperature difference between the heat source and the shell. *δ* is the thickness of the shell (m), *k* is the thermal conductivity of the shell.

**Air convection**: $\text{Re}$=$\frac{v_{air}L}{\upsilon_{air}}=\frac{\text{1∙0.05}}{\text{1.6×}\text{10}^{\text{-5}}}=$3.13×10^3^

where *L* is the characteristic length of the device. Since the *Re* number is less than 5×10^5^, the Nusselt number correlation for laminar flow over the flat plate is adopted.

Nu=$0.664{Re}^{1/2}{Pr}^{1/3}=$33.1

Thereby, $\text{h}_{\text{conv,air}}\text{=}\frac{Nuk_{air}}{L}=\frac{\text{33.1∙0.026}}{\text{0.05}}\text{=}\text{17.2}$ W/(m^2^·K).

${\text{q}^{\text{''}}}_{\text{conv,air}}=\text{h}_{\text{conv,air}}\text{∙Δ}\text{T}_{\text{air}}=$2.58×10^2^ W/m^2^.

where ${\text{q}\text{''}}_{\text{conv,air}}$ and${\text{ }\text{h}}_{\text{conv,air}}$ are the heat flux density and heat transfer coefficient of air convection.

**Liquid convection**: not available

**Radiation**: ${\text{q}^{\text{''}}}_{\text{rad}}\text{=}\text{εσ}\left( T_{shell}^{4}-T_{sky}^{4} \right)\text{=}\text{0.77∙5.67×}\text{10}^{\text{-8}}\text{∙}\text{(313}^{\text{4}}\text{-}\text{280}^{\text{4}}\text{)}\text{=}\text{160.4 W/}\text{m}^{\text{2}}$

where ${\text{q}^{\text{''}}}_{\text{rad}}$ is the radiative heat flux density. *ε* is the emissivity of the shell (Anodized aluminum), taken as 0.77^[3]^, *σ* is the Stefan-Boltzmann constant, taken as 5.67×10^-8^ W/(m^2^·K^4^). *T*_shell_ and *T*_sky_ denote shell temperature and effective sky temperature, and the latter is taken as 280 K^[4]^.

$\text{h}_{\text{rad}}\text{=}\frac{{q''}_{rad}}{\left( T_{shell}-T_{sky} \right)}\text{=4.84 }$W/(m^2^·K)

where${\text{ }\text{h}}_{\text{rad}}$ is the radiative heat transfer coefficient.

**Case 3 Solar cell modules**

*
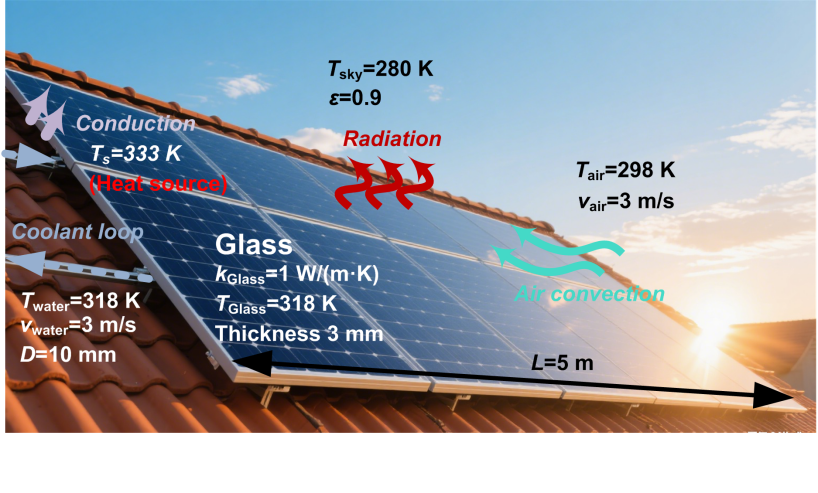
*

*Parameters*

Heating source *T*_s_=333 K, *T*_glass_=318 K (*ΔT*_glass_=15 K). *T*_air_=298 K (*ΔT*_air_=20 K).

Front glass *k*=1.0 W/(m·K), thickness *δ*=3 mm.

Wind: *v=*3 m/s. characteristic length *L*=5 m.

Back liquid-cooling (water): *D*=10 mm, *v=*3 m/s, *ΔT*_liq_=15 K

*Results*

**Conduction** (via glass): $\text{h}_{\text{cond}}\text{=}\frac{k}{\delta}\text{=}\frac{\text{1}}{\text{3×}\text{10}^{\text{-3}}}=$333 W/(m^2^·K).

${\text{q}^{\text{''}}}_{\text{cond}}\text{=}\text{h}_{\text{cond}}\text{∙}\text{Δ}\text{T}_{\text{glass}}=$5×10^3^ W/m^2^.

**Air convection**: $\text{Re}$=$\frac{v_{air}L}{\upsilon_{air}}=\frac{\text{3∙5}}{\text{1.6×}\text{10}^{\text{-5}}}=$9.4×10^5^

where *L* is the characteristic length of the device, (m). Since the *Re* number is within the range between 5×10^5^ and 1×10^7^, the Nusselt number correlation for transition region is adopted.

Nu$=(0.037{Re}^{0.8}{-871)Pr}^{1/3}$=1201

Thereby,

$\text{h}_{\text{conv,air}}\text{=}\frac{Nuk_{air}}{L}=\frac{\text{1201∙0.026}}{\text{5}}\text{=}\text{6.25}$ W/(m^2^·K).

${\text{q}^{\text{''}}}_{\text{conv,air}}=\text{h}_{\text{conv,air}}\text{∙Δ}\text{T}_{\text{air}}=$125 W/m^2^.

(If the boundary layer transitions to turbulence, ℎ_conv_ rises significantly.)

**Liquid convection** (Backwater loop): $\text{Re}$=$\frac{v_{water}D}{\upsilon_{water}}=\frac{\text{3}\text{∙0.01}}{\text{1×}\text{10}^{\text{-6}}}$=3×10^4^

Since the *Re* number is higher than 2300, the Nusselt number correlation for turbulence flow within a tube is applied.

*Nu*=$0.023{Re}^{0.8}{Pr}^{0.4}\text{=}$191.9

Thereby,

$\text{h}_{\text{conv,liq}}\text{=}\frac{Nuk_{water}}{D}=\frac{\text{191.9}\text{∙0.6}}{\text{0.01}}\text{=}\text{1.15}$×10^4^ W/(m^2^·K)

${\text{q}^{\text{''}}}_{\text{conv,liq}}=\text{h}_{\text{conv,liq}}\text{∙Δ}\text{T}_{\text{liq}}=$1.73×10^5^ W/m^2^

where ${\text{q}\text{''}}_{\text{conv,air}}$ and${\text{ }\text{h}}_{\text{conv,air}}$ are heat flux density and heat transfer coefficient of air convection.

**Radiation**: ${\text{q}^{\text{''}}}_{\text{rad}}\text{=}\text{εσ}\left( T_{s}^{4}-T_{sky}^{4} \right)\text{=}\text{0.9∙5.67×}\text{10}^{\text{-8}}\text{∙}\text{(333}^{\text{4}}\text{-}\text{298}^{\text{4}}\text{)}\text{=}\text{296 W/}\text{m}^{\text{2}}$

where ${\text{q}^{\text{''}}}_{\text{rad}}$ is the radiative heat flux density (W/m^2^). *ε* is the emissivity of crystalline silicon solar cells, taken as 0.9^[5]^, *σ* is Stefan-Boltzmann constant, taken as 5.67×10^-8^ W/(m^2^·K^4^).

$\text{h}_{\text{rad}}\text{=}\frac{{\text{q}^{\text{''}}}_{\text{rad}}}{\text{T}_{\text{s}}\text{-}\text{T}_{\text{sky}}}\text{=5.59 }$W/(m^2^·K)，where${\text{ }\text{h}}_{\text{rad}}$ is the radiative heat transfer coefficient.

**Case 4 Satellite electronics (high-power image-processor)**

*
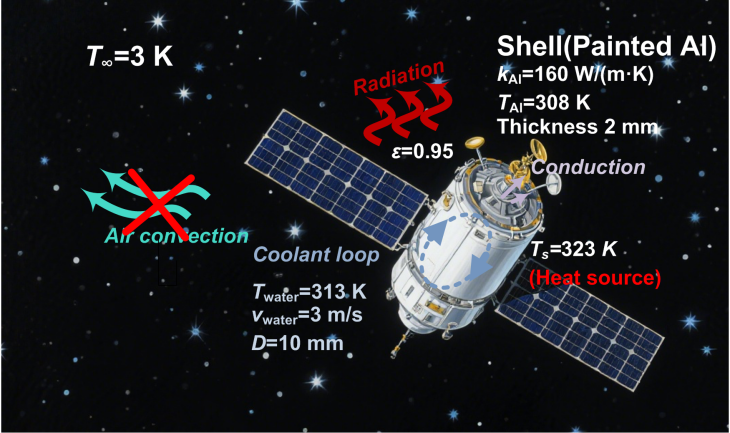
*

*Parameters*

Heating source *T*_s_=323 K, *T*_shell_=308 K (*ΔT*_cond_=15 K), *T*_liq_=313 K (*ΔT*_liq_=10 K).

Deep space *T*_∞_=3 K,

Aluminum shell *k*=160 W/(m·K), effective thickness *δ*=2 mm.

Optional liquid-cooling (water): *D*=10 mm, *v*_liq_*=*3 m/s.

*Results*

**Conduction** (via shell):$\text{h}_{\text{cond}}\text{=}\frac{k}{\delta}\text{=}\frac{\text{160}}{\text{2}\text{×}\text{10}^{\text{-3}}}=$8×10^4^ W/(m^2^·K).

${\text{q}^{\text{''}}}_{\text{cond}}\text{=}\text{h}_{\text{cond}}\text{∙}\text{Δ}\text{T}_{\text{cond}}=$1.2×10^6^ W/m^2^.

**Air convection**: not available

**Liquid convection** (if present): $\text{Re}$=$\frac{v_{water}D}{\upsilon_{water}}=\frac{\text{3}\text{∙0.0}\text{1}}{\text{1×}\text{10}^{\text{-6}}}$=3×10^4^

Since the *Re* number is higher than 2300, the Nusselt number correlation for turbulence flow within a tube is applied^[2]^.

*Nu*=$0.023{Re}^{0.8}{Pr}^{0.4}=$191.9

Thereby,

$\text{h}_{\text{conv,liq}}\text{=}\frac{Nuk_{water}}{D}\text{=}\frac{\text{191.9}\text{∙0.6}}{\text{0.0}\text{1}}\text{=}\text{1.}\text{15}\text{×}\text{10}^{4}$ W/(m^2^·K)

${\text{q}^{\text{''}}}_{\text{conv,liq}}=\text{h}_{\text{conv,liq}}\text{∙Δ}\text{T}_{\text{liq}}=$1.15×10^5^ W/m^2^

where ${\text{q}\text{''}}_{\text{conv,air}}$ and${\text{ }\text{h}}_{\text{conv,air}}$ are heat flux density (W/m^2^) and heat transfer coefficient (W·m^-2^·K^-1^) of air convection.

**Radiation**: ${\text{q}^{\text{''}}}_{\text{rad}}\text{=}\text{εσ}\left( T_{shell}^{4}-T_{\infty}^{4} \right)\text{=}\text{0.95∙5.67×}\text{10}^{\text{-8}}\text{∙}\text{(308}^{\text{4}}\text{-}\text{3}^{\text{4}}\text{)}\text{=}\text{535 W/}\text{m}^{\text{2}}$

where ${\text{q}^{\text{''}}}_{\text{rad}}$ is the radiative heat flux density (W/m^2^). *ε* is the emissivity of painted aluminum, taken as 0.95^[6]^.

$\text{h}_{\text{rad}}\text{=}\frac{{\text{q}^{\text{''}}}_{\text{rad}}}{\text{T}_{\text{s}}\text{-}\text{T}_{\infty}}\text{=}\text{1.69 }$W/(m^2^·K), which is the radiative heat transfer coefficient.

**Case 5 (High-temperature space devices, Parker Solar Probe)**

**
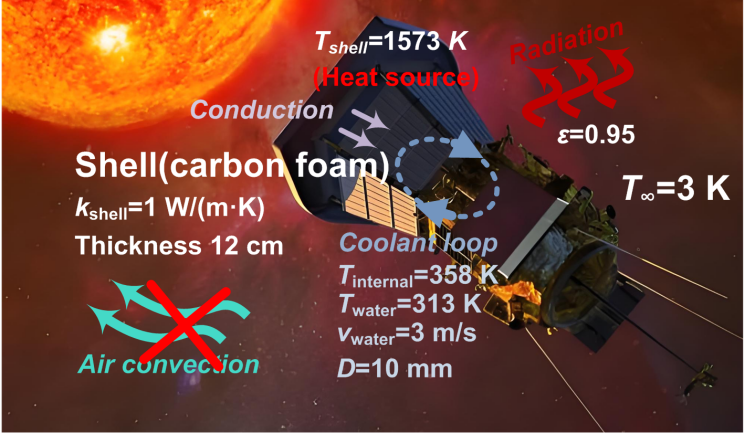
**

*Parameters*

Heating source *T*_shell_=1573 K, *T*_internal_=358 K (*ΔT*_cond_=1215 K), *T*_liq_=313 K (*ΔT*_liq_=45 K).

Deep space *T*_∞_=3 K,

Carbon foam insulation *k*=1 W/(m·K), effective thickness *δ*=120 mm.

Optional liquid-cooling (water): *D*=10 mm, *v*_liq_*=*3 m/s.

*Results*

**Conduction** (via shell):$\text{h}_{\text{cond}}\text{=}\frac{k}{\delta}\text{=}\frac{\text{1}}{\text{1}\text{2}\text{0}\text{×}\text{10}^{\text{-3}}}=$8.33 W/(m^2^·K).

${\text{q}^{\text{''}}}_{\text{cond}}\text{=}\text{h}_{\text{cond}}\text{∙}\text{Δ}\text{T}_{\text{cond}}=$1.0×10^4^ W/m^2^.

**Air convection**: not available

**Liquid convection** (if present): $\text{Re}$=$\frac{v_{water}D}{\upsilon_{water}}=\frac{\text{3∙0.}\text{01}}{\text{1×}\text{10}^{\text{-6}}}$=3×10^4^

Since the *Re* number is higher than 2300, the Nusselt number correlation for turbulence flow within a tube is applied^[2]^.

*Nu*=$0.023{Re}^{0.8}{Pr}^{0.4}=$191.9

Thereby,

$\text{h}_{\text{conv,liq}}\text{=}\frac{Nuk_{water}}{D}\text{=}\frac{\text{1}\text{91.9∙0.6}}{\text{0.0}\text{1}}\text{=}\text{1.}\text{15}$×10^5^ W/(m^2^·K)

${\text{q}^{\text{''}}}_{\text{conv,liq}}=\text{h}_{\text{conv,liq}}\text{∙Δ}\text{T}_{\text{liq}}=$5.18×10^5^ W/m^2^

where ${\text{q}\text{''}}_{\text{conv,air}}$ and${\text{ }\text{h}}_{\text{conv,air}}$ are heat flux density (W/m^2^) and heat transfer coefficient (W·m^-2^·K^-1^) of air convection.

**Radiation**: ${\text{q}^{\text{''}}}_{\text{rad}}\text{=}\text{εσ}\left( T_{shell}^{4}-T_{\infty}^{4} \right)\text{=}\text{0.}\text{6}\text{∙5.67×}\text{10}^{\text{-8}}\text{∙}{\text{(}\text{1573}}^{\text{4}}\text{-}\text{3}^{\text{4}}\text{)}\text{=}\text{2.03}$×10^5^ W/m^2^

where ${\text{q}^{\text{''}}}_{\text{rad}}$ is the radiative heat flux density (W/m^2^). *ε* is the emissivity of alumina coating on the shell surface, taken as 0.6^[6]^.

$\text{h}_{\text{rad}}\text{=}\frac{{\text{q}^{\text{''}}}_{\text{rad}}}{\text{T}_{\text{s}\text{hell}}\text{-}\text{T}_{\infty}}\text{=}\text{133}\text{.}\text{8}\text{ }$W/(m^2^·K), which is the radiative heat transfer coefficient.

The results can be summarized as follows. The effectiveness of the three heat transfer forms strongly depends on the specific application scenarios and thermal management conditions. For instance, convection (10^4^-10^6^ W/m^2^) and conduction (10^3^-10^6^ W/m^2^) generally play more prominent roles than radiation (10^2^ W/m^2^) under terrestrial conditions (enclosed chip-level devices or outdoor rooftop/ground systems), as shown in Cases 1-3. However, for electronic devices in outer space (i.e., a high-vacuum environment), where air convection is ineffective and a coolant loop is not essential, radiative cooling becomes a crucial thermal management method without any extra energy consumption. Combined systems (conduction/liquid cooling and radiation) are highly recommended. As deep space serves as an ideal cold source, radiative cooling is the inevitable pathway by which the heat dissipated from a heat source through conduction and convection is ultimately transferred into deep space. Furthermore, for high-temperature space equipment subjected to intense solar radiation, the extreme temperatures (~1300 ℃) significantly enhance radiative heat transfer (10^5^ W/m^2^). By integrating external radiative cooling with internal conduction and convection, redundant heat can be effectively removed, providing thermal protection to the internal components while simultaneously prolonging the service life of the insulation.

Comparison of heat transfer performance among three modes

| *Case* | Conduction | Convection | | Radiation |
| --- | --- | --- | --- | --- |
|  |  | Air | Water |  |
| **Case 1** (Enclosed chip-level electronics, *Computer CPU, Server, DC*) | | | | |
| Heat flux density*, W/m^2^ | 10^6^ (TIM) | 10^3^ | 10^5^ | 10^2^ |
| Heat transfer coefficient*, W/(m^2^·K) | 10^5^ | 10^2^ | 10^4^ | 10^1^ |
| **Case 2** (Wearable electronic devices, *Smartwatches, hearing aids*) | | | | |
| Heat flux density*, W/m^2^ | 10^3^(Shell) | 10^2^ | n/a | 10^2^ |
| Heat transfer coefficient*, W/(m^2^·K) | 10^2^ | 10^1^ | n/a | 10^1^ |
| **Case 3** (Outdoor rooftop/ground systems, *Building-integrated solar cell*) | | | | |
| Heat flux density*, W/m^2^ | 5×10^3^(Glass) | 10^2^ | 10^5^ | 10^2^ |
| Heat transfer coefficient*, W/(m^2^·K) | 10^2^ | 10^1^ | 10^4^ | 10^1^ |
| **Case 4** (Satellite electronics, *High-resolution image processors*) | | | | |
| Heat flux density*, W/m^2^ | 10^6^ (Al) | n/a | 10^5^ | 5×10^2^ |
| Heat transfer coefficient*, W/(m^2^·K) | 10^4^ | n/a | 10^4^ | 10^1^ |
| **Case 5** (High-temperature space devices, *Parker Solar Probe*) | | | | |
| Heat flux density*, W/m^2^ | 10^4^ | n/a | 5×10^5^ | 10^5^ |
| Heat transfer coefficient*, W/(m^2^·K) | 10^1^ | n/a | 10^4^ | 10^2^ |
| * The values are approximate to show their order of magnitudes. | | | | |

**Supplementary Table S1 | Cooling performance of typical micro/nanostructures (enhanced convection/vaporization).**

| Flow pattern | Type of design | Coolant | Material | Flow rate (cm^3^/s) | Power input (W)* | Critical Heat Flux (W/cm^2^) | Ref. |  |
| --- | --- | --- | --- | --- | --- | --- | --- | --- |
| Pumped flow | Parallel microchannels | Water | Si | 8.6 | n/a | 790 | ^[7]^ |  |
|  | Manifold microchannels | Water | Si | 1.0 | 75 | 1700 | ^[8]^ |  |
|  | Tesla valve channels | Water | Si | 0.1 | n/a | 835 | ^[9]^ |  |
|  | Piranha pin-fin | HFE-7000 | Si | 70.7 | 38.9 | 985 | ^[10, 11]^ |  |
|  | Porous-wall microchannels | Water | Si | 0.4 | 88.7 | 44 | ^[12]^ |  |
|  | High-AR groove-wall microchannels | Water | Si | 0.6 | 472.6 | 139 | ^[13]^ |  |
|  | Microchannels with micro pin fins | Water | Cu | 15.7 | 960.3 | 107 | ^[14]^ |  |
|  | Zigzag flow channel | SiO_2_-water nanofluids | SS 316L | n/a | n/a | n/a | ^[15]^ |  |
|  | Microchannels with cavities and fins | Water | Si | 0.6 | n/a | n/a | ^[16]^ |  |
|  | Spider netted microchannels | HFE-7200 | 6063 Al alloy | 10 | n/a | 150 | ^[17]^ |  |
|  | Finned microchannels | Water | Al | 3.5 | n/a | n/a | ^[18]^ |  |
|  | Microchannels with ribs and grooves | Water | Si | n/a | n/a | 100 | ^[19]^ |  |
|  | Microchannels with manifold slits | R134a | Cu | 14.7 | 424.5 | 188 | ^[20]^ |  |
|  | Multi-microchannels | R236fa | Si | 31.2 | 888 | 222 | ^[21]^ |  |
|  | Multi-microchannels | R134a | Cu | n/a | 1080 | 180 | ^[22]^ | |
| Capillary | Micropillars | Water | Si | n/a | 45.2* | 1130 | ^[23]^ | |
|  | Micro-membrane on microchannels | Water | Cu | n/a | 23.3 | 140 | ^[24]^ | |
|  | Micropillar wicks | Water | Si | n/a | 46* | 46 | ^[25]^ | |
|  | Pin-fins with microchannels | Water | Si | n/a | 45.7* | 733 | ^[26]^ | |
|  | Cylindrical pillar wick | Water | Si | n/a | 21.6* | 135 | ^[27]^ | |
|  | Nanostructured micropost | Water | Cu | n/a | 32* | 800 | ^[28]^ | |
|  | Micropost | Water | Cu | n/a | 5* | 20 | ^[29]^ | |
|  | Inverse opal wicks | Water | Cu | n/a | 182.3 | 225 | ^[30]^ | |
|  | Micropillar array | Ethanol | Si | n/a | 29.8 | 119 | ^[31]^ | |
|  | Nanowired surfaces | Water | Cu | n/a | n/a | n/a | ^[32]^ | |
|  | Nanopillars | Water | Si | n/a | n/a | 214 | ^[33]^ | |
| Pool boiling | Nanoparticle surfaces | Water | Cu & CuO | n/a | 890 | 223 | ^[34, 35]^ | |
|  | TIP Structures | Water | Si | n/a | 1020 | 256 | ^[36]^ | |
|  | Inverse opal surfaces | Water | Cu/Ni | n/a | 488 | 122 | ^[37]^ | |
| Jet impingement | Hydrofoil micro pin fin | R134a | Si | 12.7 | 7 | 175 | ^[38]^ | |
|  | Square micropin fin | FC-72 | Si | n/a | 120 | 120 | ^[39]^ | |
|  | Square micropin fin | HFE-7100 | Cu | 7 | 339.3 | 53 | ^[40]^ | |
| Spray cooling | Microfin surfaces | R134a | Cu | 6.5 | 420 | 210 | ^[41]^ | |
|  | Squared fin surfaces | FC-72 | Cu | 5 | 122 | 85 | ^[42]^ | |
|  | Multi-microchannels | HFE-7100 | Cu | 1.67 | 200 | 100 | ^[43]^ | |
| * The calculated power value derived from heat flux and heat exchange area | | | | | | | | |

**Supplementary Table S2 | Cooling performance of typical micro/nanostructures (radiative cooling).**

| Year | Materials | Transparency | Colorful | Emissivity | Cooling power (W/m^2^) | Temperature reduction (℃) | Ref. |
| --- | --- | --- | --- | --- | --- | --- | --- |
| 2022 | Rod-like particles | Reflective | No | 0.950 | 97.4 | 5.7 | ^[44]^ |
| 2022 | TiO_2_/Y_2_O_3_/PDMS | Reflective | No | 0.949 | 72.5 | 7.7 | ^[45]^ |
| 2022 | Zn/Ag/Glass | Transparent | No | 0.91 | n/a | 12.5 | ^[46]^ |
| 2022 | SiO_2_ | Reflective | Yes | 0.95 | 143.7 | 7.1 | ^[47]^ |
| 2023 | Polymer metasurface structures with Ag coating | Reflective | No | 0.961 | 129.8 | 7 | ^[48, 49]^ |
| 2023 | sodium polyacrylate (PAAS) | Reflective | No | 0.990 | 190 (Evaporation) | 5 | ^[50]^ |
| 2023 | PDMS/SiO_2/_Ag/Glass | Transparent | No | 0.95 | n/a | 7 | ^[51]^ |
| 2023 | PVC/BN nanoplates/  Quinacridone/iron oxide green/Prussian blue | Reflective | Yes | 0.93 | 120 | 7.1 | ^[52]^ |
| 2023 | Cellular ceramic | Reflective | No | 0.965 | ＞130 | 3.8 | ^[53]^ |
| 2024 | Sawtooth grating with a nanoPE film | Reflective | No | 0.978 | 114 (Vertical) | 2.5±0.7 | ^[54]^ |
| 2024 | PDMS/SiO_2_@WA | Reflective | No | 0.902 | 241.66 | 13.5 (Winter)  /20.2 (Summer) | ^[55]^ |
| 2024 | PDMS with diffraction grating and fused SiO_2_/Ti/Ag film | Reflective | No | ~1 | 530 | Maintaining at 25℃ | ^[54]^ |
| 2024 | Modified SiP-C with PVDF Coating | Reflective | No | 0.980 | 81.76 (Daytime)/  118.32 (Night) | 10.5 | ^[56]^ |
| 2024 | PDMS | Transparent | No | 0.98 | 97 | 1.7 | ^[57]^ |
| 2024 | Multilayer film with aluminum-doped zinc oxide nanocrystals | Reflective | No | 0.942 | 67.3 | 2.84 | ^[58]^ |
| 2024 | Cholesteric liquid Crystals/Ag/PDMS | Reflective | Yes | 0.918 | 19.5 | 3.1 | ^[59]^ |
| 2024 | A gradient nanoporous MgO ceramic | Reflective | No | 0.950 | 102.7 (Daytime)/  77.1 (Night) | 2.2 (Daytime)/  5.1 (Night) | ^[60]^ |
| 2024 | c-PI/PMMA-SiO_2_ | Transparent | No | 0.946 | 109 | 6.9 | ^[61]^ |
| 2024 | PDMS elastomer lubricated with silicone oil coating | Reflective | No | n/a | ~200 | n/a | ^[62]^ |
| 2024 | PDMS/TiO_2_/ZnO/ZnS/SiO_2_ | Reflective | Yes | n/a | 11.2-38.2 | 1.7-5.5 | ^[63]^ |
| 2024 | PDMS_3_PG_3/t4_ | Transparent | No | 0.922 | n/a | 4.9 | ^[64]^ |
| 2024 | Nanoffber aerogel polymeric nanocomposite | Transparent | No | 0.91 | n/a | 3.2 | ^[65]^ |
| 2025 | A heteroporous nanocomposite film | Reflective | No | 0.973 | 114 | 11 | ^[66]^ |
| 2025 | PMMA/PVDF foam | Reflective | No | 0.973 | 104 | 15 | ^[67]^ |
| 2025 | ZrO_2_-Al_2_O_3_ nanofiber membrane | Reflective | No | 0.956 | 125 | 6.6 | ^[68]^ |
| 2025 | Cement (Al_2_O_3_, CaCO_3_, CaSO_4_, SiO_2_) | Reflective | No | 0.960 | 96 | 5.4 | ^[69]^ |
| 2025 | PMMA | Reflective | Yes | 0.934 | n/a | 10.2 (night)  /7.2 (day) | ^[70]^ |
| 2025 | A cementitious matrix with a porous calcium silicate hydrate network | Reflective | No | 0.950 | 140 | 4.5 | ^[71]^ |
| 2025 | Thermochromic microcapsule and silica aerogel | Reflective | Yes | 0.922 | n/a | 2.8 | ^[72]^ |
| 2025 | SrAl_2_O_4_ and structured cellulose nanocrystals | Reflective | Yes | 0.90 | n/a | 11.3 | ^[73]^ |
| 2025 | Y_2_O_3_/TiO_2_ | Reflective | Yes | 0.959 | n/a | 3.9 | ^[74]^ |

**Supplementary References**

[1] Burmeister, L. C., Convective heat transfer. **1993**: John Wiley & Sons.

[2] Incropera, F., Fundamentals of heat and mass transfer. **2011**: John Wiley & Sons.

[3] The Engineering ToolBox. Emissivity Coefficients of Common Materials: Data & Reference Guide https://www.engineeringtoolbox.com/emissivity-coefficients-d_447.html [cited Aug 1 **2025**.];

[4] Li, M., Jiang, Y.,Coimbra, C. F. M.,On the determination of atmospheric longwave irradiance under all-sky conditions. *Sol. Energy*, **2017**. *144*: 40-48.

[5] Riverola, A., Mellor, A., Alonso Alvarez, D., Ferre Llin, L., Guarracino, I., Markides, C. N., Paul, D. J., Chemisana, D.,Ekins-Daukes, N.,Mid-infrared emissivity of crystalline silicon solar cells. *Sol. Energy Mater. Sol. Cells*, **2018**. *174*(1): 607-615.

[6] Tuttle, J., Canavan, E., DiPirro, M.,Li, X.,The total hemispheric emissivity of painted aluminum honeycomb at cryogenic temperatures. *AIP Conf. Proc.*, **2014**. *1573*(1): 590-596.

[7] Tuckerman, D. B.,Peas, R. F. W.,High-performance heat sinking for VLSI. *IEEE Electron Device Lett.*, **1981**. *2*(5): 126-129.

[8] van Erp, R., Soleimanzadeh, R., Nela, L., Kampitsis, G.,Matioli, E.,Co-designing electronics with microfluidics for more sustainable cooling. *Nature*, **2020**. *585*(7824): 211-216.

[9] Li, W., Yang, S., Chen, Y., Li, C.,Wang, Z.,Tesla valves and capillary structures-activated thermal regulator. *Nat. Commun.*, **2023**. *14*: 3996.

[10] Woodcock, C., Ng'oma, C., Sweet, M., Wang, Y., Peles, Y.,Plawsky, J.,Ultra-high heat flux dissipation with Piranha Pin Fins. *Int. J. Heat Mass Transfer*, **2019**. *128*: 504-515.

[11] Woodcock, C., Yu, X., Plawsky, J.,Peles, Y.,Piranha Pin Fin (PPF)—Advanced flow boiling microstructures with low surface tension dielectric fluids. *Int. J. Heat Mass Transfer*, **2015**. *90*: 591-604.

[12] Zong, L. X., Xia, G. D., Jia, Y. T., Liu, L., Ma, D. D.,Wang, J.,Flow boiling instability characteristics in microchannels with porous-wall. *Int. J. Heat Mass Transfer*, **2020**. *146*: 118863.

[13] Cheng, X.,Wu, H.,Enhanced flow boiling performance in high-aspect-ratio groove-wall microchannels. *Int. J. Heat Mass Transfer*, **2021**. *164*: 120468.

[14] Deng, D., Wan, W., Qin, Y., Zhang, J.,Chu, X.,Flow boiling enhancement of structured microchannels with micro pin fins. *Int. J. Heat Mass Transfer*, **2017**. *105*: 338-349.

[15] Zhou, X., Zeng, C., Song, Y., Jiao, M., Zhang, F.,Liu, M.,Experimental study on heat transfer and flow resistance performance of a microchannel heat exchanger with zigzag flow channels. *Prog. Nucl. Energy*, **2022**. *147*: 104190.

[16] Li, Y., Wang, Z., Yang, J.,Liu, H.,Thermal and hydraulic characteristics of microchannel heat sinks with cavities and fins based on field synergy and thermodynamic analysis. *Appl. Therm. Eng.*, **2020**. *175*: 115348.

[17] Tan, H., Du, P., Zong, K., Meng, G., Gao, X.,Li, Y.,Investigation on the temperature distribution in the two-phase spider netted microchannel network heat sink with non-uniform heat flux. *Int. J. Therm. Sci.*, **2021**. *169*: 107079.

[18] Zhang, F., Wu, B.,Du, B.,Heat transfer optimization based on finned microchannel heat sink. *Int. J. Therm. Sci.*, **2022**. *172*: 107357.

[19] Wang, G., Niu, D., Xie, F., Wang, Y., Zhao, X.,Ding, G.,Experimental and numerical investigation of a microchannel heat sink (MCHS) with micro-scale ribs and grooves for chip cooling. *Appl. Therm. Eng.*, **2015**. *85*: 61-70.

[20] Madhour, Y., Olivier, J., Costa-Patry, E., Paredes, S., Michel, B.,Thome, J. R.,Flow Boiling of R134a in a multi-microchannel heat sink with hotspot heaters for energy-efficient microelectronic CPU cooling applications. *IEEE Transactions on Components, Packaging and Manufacturing Technology*, **2011**. *1*(6): 873-883.

[21] Agostini, B., Thome, J. R., Fabbri, M., Michel, B., Calmi, D.,Kloter, U.,High heat flux flow boiling in silicon multi-microchannels – Part I: Heat transfer characteristics of refrigerant R236fa. *Int. J. Heat Mass Transfer*, **2008**. *51*(21-22): 5400-5414.

[22] Park, J. E.,Thome, J. R.,Critical heat flux in multi-microchannel copper elements with low pressure refrigerants. *Int. J. Heat Mass Transfer*, **2010**. *53*(1-3): 110-122.

[23] Cai, S. Q.,Bhunia, A.,Geometrical effects of wick structures on the maximum phase change capability. *Int. J. Heat Mass Transfer*, **2014**. *79*: 981-988.

[24] Dai, X., Yang, F., Yang, R., Lee, Y.-C.,Li, C.,Micromembrane-enhanced capillary evaporation. *Int. J. Heat Mass Transfer*, **2013**. *64*: 1101-1108.

[25] Adera, S., Antao, D., Raj, R.,Wang, E. N.,Design of micropillar wicks for thin-film evaporation. *Int. J. Heat Mass Transfer*, **2016**. *101*: 280-294.

[26] Ćoso, D., Srinivasan, V., Lu, M.-C., Chang, J.-Y.,Majumdar, A.,Enhanced heat transfer in biporous wicks in the thin liquid film evaporation and boiling regimes. *J. Heat Transfer*, **2012**. *134*: 101501.

[27] Cai, S. Q.,Bhunia, A.,Characterization of phase change heat and mass transfers in monoporous silicon wick structures. *J. Heat Transfer*, **2014**. *136*: 072001.

[28] Nam, Y., Sharratt, S., Cha, G.,Ju, Y. S.,Characterization and modeling of the heat transfer performance of nanostructured Cu micropost wicks. *J. Heat Transfer*, **2011**. *133*: 101502.

[29] Sharratt, S., Peng, C.,Ju, Y. S.,Micropost evaporator wicks with improved phase change heat transfer performance. *Int. J. Heat Mass Transfer*, **2012**. *55*(21-22): 6163-6169.

[30] Barako, M. T., Sood, A., Zhang, C., Wang, J., Kodama, T., Asheghi, M., Zheng, X., Braun, P. V.,Goodson, K. E.,Quasi-ballistic Electronic Thermal Conduction in Metal Inverse Opals. *Nano Lett.*, **2016**. *16*(4): 2754-2761.

[31] Zhou, D., Chen, Y., Gao, W.,Xin, G.,A novel thermal management scheme of 3D-IC based on loop heat pipe. *Int. J. Therm. Sci.*, **2024**. *199*: 108906.

[32] Wang, Y., Xu, C., Ruan, D., Chun, J.,Ma, X.,Heat transfer characteristics of capillary pumping-replenishment evaporation on nanowire clusters surfaces with V-grooves. *CIESC Journal*, **2024**. *75*(10): 3424-3436.

[33] Kim, B. S., Lee, H., Shin, S., Choi, G.,Cho, H. H.,Interfacial wicking dynamics and its impact on critical heat flux of boiling heat transfer. *Appl. Phys. Lett.*, **2014**. *105*: 191601.

[34] Khan, S. A., Sezer, N., Ismail, S.,Koç, M.,Design, synthesis and nucleate boiling performance assessment of hybrid micro-nano porous surfaces for thermal management of concentrated photovoltaics (CPV). *Energy Convers. Manage.*, **2019**. *195*: 1056-1066.

[35] Khan, S. A., Sezer, N.,Koç, M.,Design, fabrication and nucleate pool-boiling heat transfer performance of hybrid micro-nano scale 2-D modulated porous surfaces. *Appl. Therm. Eng.*, **2019**. *153*: 168-180.

[36] Song, Y., Díaz‐Marín, C. D., Zhang, L., Cha, H., Zhao, Y.,Wang, E. N.,Three‐tier hierarchical structures for extreme pool boiling heat transfer performance. *Adv. Mater.*, **2022**. *34*(2200899).

[37] Fang, J., Cheng, H.,Fan, D.,Enhanced heat transfer using wafer-scale crack-free well-ordered porous structure surface. *Int. Commun. Heat Mass Transf.*, **2023**. *148*: 107018.

[38] Ndao, S., Peles, Y.,Jensen, M. K.,Experimental investigation of flow boiling heat transfer of jet impingement on smooth and micro structured surfaces. *Int. J. Heat Mass Transfer*, **2012**. *55*(19-20): 5093-5101.

[39] Guo, D., Wei, J. J.,Zhang, Y. H.,Enhanced flow boiling heat transfer with jet impingement on micro-pin-finned surfaces. *Appl. Therm. Eng.*, **2011**. *31*(11-12): 2042-2051.

[40] Joshi, S. N.,Dede, E. M.,Effect of sub-cooling on performance of a multi-jet two phase cooler with multi-scale porous surfaces. *Int. J. Therm. Sci.*, **2015**. *87*: 110-120.

[41] Zhou, R., Pan, Y.-H., Zheng, L., Fu, H., Shen, Y., Hao, M., Yang, Y., Yan, J., Chen, H.,Cheng, W.-L.,Study on heat transfer enhancement of spray cooling with bionic vein channel structured surface. *Appl. Therm. Eng.*, **2024**. *246*: 122977.

[42] Chien, L.-H.,Chang, C.-Y.,An experimental study of two-phase multiple jet cooling on finned surfaces using a dielectric fluid. *Appl. Therm. Eng.*, **2011**. *31*(11-12): 1983-1993.

[43] Sung, M. K.,Mudawar, I.,Effects of jet pattern on two-phase performance of hybrid micro-channel/micro-circular-jet-impingement thermal management scheme. *Int. J. Heat Mass Transfer*, **2009**. *52*(13-14): 3364-3372.

[44] Huang, J., Fan, D.,Li, Q.,Structural rod-like particles for highly efficient radiative cooling. *MIIT Key Laboratory of Thermal Control of Electronic Equipment, School of Energy and Power Engineering, Nanjing University of Science and Technology, Jiangsu, Nanjing, 210094, China*, **2022**. *25C*: 100955.

[45] Du, T., Niu, J., Wang, L., Bai, J., Shengxing Wang, Shijie Li,Fan, Y.,Daytime radiative cooling coating based on the Y_2_O_3_/TiO_2_ microparticle-embedded PDMS polymer on energy-saving buildings. *ACS Appl. Mater. Interfaces*, **2022**. *14*(45): 51351-51360.

[46] Yanan Li, Chen, i., Yu, L., Pang, D., Yan, H.,Chen, M.,Janus interface engineering boosting visibly transparent radiative cooling for energy saving. *ACS Appl. Mater. Interfaces*, **2023**. *15*(3): 4122-4131.

[47] Ding, Z., Pattelli, L., Xu, H., Sun, W., Li, X., Pan, L., Zhao, J., Wang, C., Zhang, X., Song, Y., Qiu, J., Li, Y.,Yang, R.,Iridescent daytime radiative cooling with no absorption peaks in the visible range. *Small*, **2022**. *18*(25): e2202400.

[48] Lin, K.-T., Nian, X., Li, K., Han, J., Zheng, N., Lu, X., Guo, C., Lin, H.,Jia, B.,Highly efficient flexible structured metasurface by roll-to-roll printing for diurnal radiative cooling. *eLight*, **2023**. *3*: 22.

[49] Nian, X., Lin, K.-T., Li, K., Hei, J., Han, J., Li, Y., Guo, C., Lin, H., Zheng, J.,Jia, B.,A high-power, flexible, and magnetically attachable radiative cooling film. *Engineering*, **2024**(49): 122-133.

[50] Galib, R. H., Tian, Y., Lei, Y., Dang, S., Li, X., Yudhanto, A., Lubineau, G.,Gan, Q.,Atmospheric-moisture-induced polyacrylate hydrogels for hybrid passive cooling. *Nat. Commun.*, **2023**. *14*(1): 6707.

[51] Jin, Y., Jeong, Y.,Yu, K.,Infrared-reflective transparent hyperbolic metamaterials for use in radiative cooling windows. *Adv. Funct. Mater.*, **2023**. *33*(1): 2207940.

[52] Lin, Y., Qin, C., Fang, L., Wang, J.,Li, D.,Colored polymeric films with a bilayer porous design for efficient subambient radiative cooling. *ACS Applied Polymer Materials*, **2024**. *6*(1): 722-731.

[53] Lin, K., Chen, S., Zeng, Y., Ho, T. C., Zhu, Y., Wang, X., Liu, F., Huang, B., Chao, C. Y.-H., Wang, Z.,Tso, C. Y.,Hierarchically structured passive radiative cooling ceramic with high solar reflectivity. *Science*, **2023**. *382*(6671): 691-697.

[54] Fei, J., Zhang, X., Han, D., Lei, Y., Xie, F., Zhou, K., Koh, S.-W., Ge, J., Zhou, H., Wang, X., Wu, X., Tan, J.-Y., Gu, Y., Long, Y., Koh, Z. H., Wang, S., Du, P., Mi, T., Ng, B.-F., Cai, L., Feng, C., Gan, Q.,Li, H.,Passive cooling paint enabled by rational design of thermal-optical and mass transfer properties. *Science*, **2025**. *388*(6751): 1044-1049.

[55] Yu, Y., Wei, L., Pang, Z., Wu, J., Dong, Y., Pan, X., Hu, J., Qu, J., Li, J., Tian, D.,Cai, Y.,Multifunctional wood composite aerogel with integrated radiant cooling and fog–water harvesting for all-day building energy conservation. *Adv. Funct. Mater.*, **2025**. *35*(5): 2414590.

[56] Wang, T.-Y.,Huang, C.-W.,Harnessing optimized SiO₂ particles for enhanced passive daytime radiative cooling in thin composite coatings. *Sol. Energy Mater. Sol. Cells*, **2024**. *278*: 113146.

[57] Gan Huang, A. R. Y.,  Kishin Matsumori, Prit Patel, Anurag Datla, Karina Trindade, Enkhlen Amarsanaa, Tonghan Zhao, Uwe Köhler, Dmitry Busko, Bryce S Richards,Radiative cooling and indoor light management enabled by a transparent and self-cleaning polymer-based metamaterial. *Nat. Commun.*, **2024**. *15*(1): 3798.

[58] Jia, Y., Liu, D., Chen, D., Jin, Y., Ge, Y., Zhang, W., Chen, C., Cheng, B., Wang, X., Liu, T., Li, M., Zu, M., Wang, Z.,Cheng, H.,Realizing sunlight-induced efficiently dynamic infrared emissivity modulation based on aluminum-doped zinc oxide nanocrystals. *Adv. Sci.*, **2024**. *11*(36): e2405962.

[59] Zhao, J., Nan, F., Zhou, L., Huang, H., Zhou, G., Zhu, Y.-f.,Ou, Q.,Free-standing, colored, polymer film with composite opal photonic crystal structure for efficient passive daytime radiative cooling. *Sol. Energy Mater. Sol. Cells*, **2023**. *251C*: 112136.

[60] Wang, X., Liu, D., Wan, Z., Wang, Z., Yu, J., Cheng, H., Jia, Y.,Shi, T.,A gradient nanoporous radiative cooling ceramic with high spectral selectivity. *Chem. Eng. J.*, **2024**. *Vol.500*(No.0): 157344.

[61] Lee, K. W., Yi, J., Kim, M. K., null,Kim, D. R.,Transparent radiative cooling cover window for flexible and foldable electronic displays. *Nat. Commun.*, **2024**. *15*(1): 4443.

[62] Ahmad, S., Siddiqui, A. R., Yang, K., Zhou, M., Ali, H. M., Hardian, R., Szekely, G., Daniel, D., Yang, S.,Gan, Q.,Lubricated surface in a vertical double-sided architecture for radiative cooling and atmospheric water harvesting. *Adv. Mater.*, **2024**. *36*(51): e2404037.

[63] Keawmuang, H., Badloe, T., Lee, C., Park, J.,Rho, J.,Inverse design of colored daytime radiative coolers using deep neural networks. *Sol. Energy Mater. Sol. Cells*, **2024**. *271*: 112848.

[64] Su, W., Liu, H., Chang, Z., Li, W., Yan, S., Li, J., Geng, F., Yao, X., Ma, M., Wang, K.,Jiang, J.,Sustainable passive radiation cooling transparent film for mobile phone protective screens. *J. Colloid Interface Sci.*, **2025**. *680*(Part A): 859-867.

[65] Lian, M., Liu, S., Ding, W., Wang, Y., Zhu, T., Hu, N., Fan, W., Miao, Y.-E., Zhang, C.,Tianxi Liu 214122, P. C.,A mechanically robust and optically transparent nanofiber-aerogel-reinforcing polymeric nanocomposite for passive cooling window. *Chem. Eng. J.*, **2024**. *498*: 154973.

[66] Tang, W., Zhan, Y., Yang, J., Meng, X., Zhu, X., Li, Y., Lin, T., jiang, L., Zhao, Z.,Wang, S.,Cascaded Heteroporous Nanocomposites for Thermo-Adaptive Passive Radiation Cooling. *Adv. Mater.*, **2024**. *36*(36): 2310923.

[67] Zhong, W., Chen, Y., Hu, D., Sun, J., Jia, X.,Zhao, L.,Unprecedented ultra-high expansion ratio foam for innovative architecture. *Adv. Sci.*, **2025**. *12*(19): e2501188.

[68] Chen, D.-C., Hwang, C.-W., Chang, C. Y., Kuo, C.-L., Chen, H.-L., Lan, P.-H., Tsai, M.-T., Wang, T.-W.,Wan, D.,Superdurable, flexible ceramic nanofibers for sustainable passive radiative cooling. *ACS Nano*, **2025**. *19*(31): 28280-94.

[69] Lu, G., Du, F., Wang, Z., Wu, F., Zuo, W., Xu, X., Wu, Z., Liu, C., Yang, R., Tian, Y., Hu, Z., Zhao, D., Guo, C., Li, T., She, W.,Miao, C.,Scalable metasurface-enhanced supercool cement. *Sci. Adv.*, **2025**. *11*(34): eadv2820.

[70] Hou, X., Zhang, K., Lai, X., Hu, L., Vogelbacher, F., Song, Y., Jiang, L.,Li, M.,Brilliant colorful daytime radiative cooling coating mimicking scarab beetle. *Matter*, **2025**. *8*(1): 101898.

[71] Dong, Y., Sun, L., Guo, Y., Xu, J., Jin, Z., Wang, Z., Zhang, W., Xia, Y., Huang, H., Xia, X.,Zhang, J.,Multifunctional Janus-Coated Metafabric for Personal Thermal Comfort and Energy Efficient Buildings. *ACS Appl. Mater. Interfaces*, **2025**. *17*(9): 14499-14509.

[72] Hu, X., Zhang, Y., Cai, W., Ming, Y., Yu, R., Chen, D., Ho, M.-P. M., Wang, F., Kan, C.-W., Noor, N.,Fei, B.,Colorful and temperature-adaptive radiative coolers for all-season thermal management applications. *Renewable Energy*, **2025**. *242*: 122447.

[73] Zhou, Y., Lu, C.,Xiong, R.,Hierarchical nanocellulose photonic design for synergistic colored radiative cooling. *ACS Nano*, **2025**. *19*(4): 5029-5039.

[74] Wang, T., Xuan, Q., Dong, Y., Lei, D.,Dai, J.-G.,Bilayer fluorescent colored radiative cooling coatings for building energy saving. *Energy*, **2025**. *332*: 137292.
